# Production of Amyloid-β in the Aβ-Protein-Precursor Proteolytic Pathway Is Discontinued or Severely Suppressed in Alzheimer’s Disease-Affected Neurons: Contesting the ‘Obvious’

**DOI:** 10.3390/genes16010046

**Published:** 2025-01-02

**Authors:** Vladimir Volloch, Sophia Rits-Volloch

**Affiliations:** 1 Department of Developmental Biology, Harvard School of Dental Medicine, Boston, MA 02115, USA; 2 Division of Molecular Medicine, Children’s Hospital, Boston, MA 02115, USA; 3 Department of Biological Chemistry and Molecular Pharmacology, Harvard Medical School, Boston, MA 02115, USA

**Keywords:** Amyloid Cascade Hypothesis 2.0 (ACH2.0), conventional and unconventional Alzheimer’s disease (AD), AβPP-independent generation of the C99 fragment, neuronal integrated stress response (ISR), AD as the disease of the neuronal ISR, ISR-mediated suppression of the AβPP proteolytic pathway and dyshomeostasis of AβPP in AD-affected neurons, C99 as the driver of AD, concurrent inhibition of the ISR and activation of BACE1 and BACE2 as composite AD therapy, RNA-dependent amplification of human AβPP mRNA, design of AD models

## Abstract

A notion of the continuous production of amyloid-β (Aβ) via the proteolysis of Aβ-protein-precursor (AβPP) in Alzheimer’s disease (AD)-affected neurons constitutes both a cornerstone and an article of faith in the Alzheimer’s research field. The present Perspective challenges this assumption. It analyses the relevant empirical data and reaches an unexpected conclusion, namely that in AD-afflicted neurons, the production of AβPP-derived Aβ is either discontinued or severely suppressed, a concept that, if proven, would fundamentally change our understanding of the disease. This suppression, effectively self-suppression, occurs in the context of the global inhibition of the cellular cap-dependent protein synthesis as a consequence of the neuronal integrated stress response (ISR) elicited by AβPP-derived intraneuronal Aβ (*i*Aβ; hence self-suppression) upon reaching certain levels. Concurrently with the suppression of the AβPP proteolytic pathway, the neuronal ISR activates in human neurons, but not in mouse neurons, the powerful AD-driving pathway generating the C99 fragment of AβPP independently of AβPP. The present study describes molecular mechanisms potentially involved in these phenomena, propounds novel approaches to generate transgenic animal models of AD, advocates for the utilization of human neuronal cells-based models of the disease, makes verifiable predictions, suggests experiments designed to validate the proposed concept, and considers its potential research and therapeutic implications. Remarkably, it opens up the possibility that the conventional production of AβPP, BACE enzymes, and γ-secretase components is also suppressed under the neuronal ISR conditions in AD-affected neurons, resulting in the dyshomeostasis of AβPP. It follows that whereas conventional AD is triggered by AβPP-derived *i*Aβ accumulated to the ISR-eliciting levels, the disease, in its both conventional and unconventional (triggered by the neuronal ISR-eliciting stressors distinct from *i*Aβ) forms, is driven not (or not only) by *i*Aβ produced in the AβPP-independent pathway, as we proposed previously, but mainly, possibly exclusively, by the C99 fragment generated independently of AβPP and not cleaved at the γ-site due to the neuronal ISR-caused deficiency of γ-secretase (apparently, the AD-driving “substance X” predicted in our previous study), a paradigm consistent with a dictum by George Perry that Aβ is “central but not causative” in AD. The proposed therapeutic strategies would not only deplete the driver of the disease and abrogate the AβPP-independent production of C99 but also reverse the neuronal ISR and ameliorate the AβPP dyshomeostasis, a potentially significant contributor to AD pathology.

## 1. The Problem: Massive Overexpression of Aβ in Transgenic Animal Models Should Translate into the Full Spectrum of Alzheimer’s Pathology, but Does Not

The recently advanced Amyloid Cascade Hypothesis 2.0 (ACH2.0) theory of Alzheimer’s disease (AD) [1,2,3,4,5,6,7,8,9], so named because it retains (albeit partially) the centrality of Aβ postulated in the initial Amyloid Cascade Hypothesis theory of AD [10], provided a complete and satisfactory explanation for the entire phenomenology of the disease and the related empirical data. One class of AD-related observations, however, has remained unexplained, indicative of the necessity for a deeper understanding of the disease. The phenomenon in question is the outcome of an acute overexpression of human AβPP and, consequently, Aβ in the attempted transgenic animal AD models. In these experimental systems, Aβ is massively overproduced from multiple, in some cases nearly a hundred, human AβPP transgenes carrying, in most instances, various combinations of mutations known to cause the early onset familial AD (FAD). In addition, many of these transgenic models also express human presenilins (PSEN; components of the γ-secretase complex) carrying FAD-causing mutations. The adjective “attempted” employed above emphasizes the fact that these experimental systems failed as AD models. They do produce extensive extracellular Aβ deposits but, as argued below, this may have little relevance to the disease. They do exhibit a degree of neurodegeneration and cognitive impairment but, as elaborated in the following sections, both features are attributable not to AD but rather constitute the effects of the neuronal integrated stress response (ISR) elicited by the overproduced Aβ accumulated intraneuronally via the mechanisms described below. Most tellingly, these experimental systems fail to form neurofibrillary tangles (NFTs) consisting of hyper-phosphorylated tau protein and constituting the only major hallmark of AD (“the only” emphasizes the exclusion of extracellular Aβ deposits as a hallmark of AD).

Although the attempted transgenic animal models of AD have been designed on the basis of the presumptions of the ACH, they should, for the reasons stipulated below, be effective, i.e., result in the formation of NFTs and development of AD, also within the framework of the ACH2.0. That this does not occur indicates that the latter needs to be adjusted to accommodate the empirical observations. Such an adjustment is the main goal of the present study. To achieve it, the present Perspective describes the fundamentals of the ACH2.0 theory of AD and applies its principles to the analysis of the failure of the current transgenic animal AD models to develop the disease. This analysis results in an unexpected conclusion, namely that the production of AβPP and/or AβPP-derived Aβ is either discontinued or substantially suppressed not only in these model systems but also in the AD-afflicted human neurons. The present study suggests approaches to construct adequate transgenic animal models of AD, advocates for the advantages of human neuronal cells-based AD models, makes verifiable predictions, indicates that the production of BACE1 and BACE2, and even of γ-secretase, can also be suppressed in the AD-affected neurons, proposes that the disease may be driven by C99 generated independently of AβPP, and discusses the potential therapeutic implication of its conclusions.

## 2. Extracellular Aβ and AD: Amyloid Cascade Hypothesis (ACH) Has Outlived Its Pertinence

The initial Amyloid Cascade Hypothesis theory of AD was formulated in 1992. It postulated, in the words of its authors, that “the extracellular deposition of amyloid β protein, the main component of the plaques, is the causative agent of Alzheimer’s pathology and that the neurofibrillary tangles, cell loss, vascular damage, and dementia follow as the direct result of this deposition” [10]. By the time of its introduction, the purported correlation of the occurrence of AD with the deposition of extracellular plaques containing Aβ was a commonly accepted feature. What prompted the formulation of the ACH was a discovery, made the preceding year, of an Aβ-associated mutation (the first of many to follow) that segregated with the disease [11]. At the time of its formulation, the ACH was consistent with the accumulated body of empirical data, offered verifiable predictions, and was enthusiastically accepted. As the field developed, however, it became increasingly clear that extracellular Aβ neither causes nor drives Alzheimer’s disease. Although its depletion in transgenic animals acutely overexpressing human AβPP yielded highly promising results [12,13,14] (because of the inadequacy of models, as addressed below and elsewhere [7]), a comparable depletion in numerous clinical trials in advanced symptomatic AD conferred no benefits whatsoever to the treated patients; in fact, only highly deleterious effects were observed [15,16] (readers interested in the mechanistic ACH2.0-based interpretation of the marginal effects of lecanemab and donanemab [17,18,19,20,21] and, potentially, of any extracellular Aβ-depleting drug in very early symptomatic AD are referred to [3,6]). In addition, with the accumulation of the empirical data, as relevant PET-scan techniques developed, it transpired that the correlation of the extents of deposition of extracellular Aβ with the incidence of AD is, in fact, not good. In a large fraction of the aging human population levels of deposited extracellular Aβ are comparable with and sometimes even exceed those seen in AD but no symptoms of the disease are observed [22,23,24,25,26,27,28]. The disconnection of extracellular Aβ and AD is further indicated by the observation that some AD patients have normal, i.e., not excessive, levels of extracellular Aβ [29].

## 3. Amyloid-β Is Central in Conventional AD but It Is Intraneuronal Rather than Extracellular Aβ

The main point of the preceding section is that extracellular Aβ has little relevance to Alzheimer’s disease. On the other hand, however, the centrality of Aβ in conventional AD remains irrefutable. This notion is based on the observations that ALL detected mutations that either cause conventional AD or confer protection from the disease have an effect only on various aspects of Aβ such as its structure, dynamics, and metabolism. As was mentioned above, the first such mutation was detected in 1991 [11]. In the intervening thirty plus years, numerous additional mutations within both AβPP and PSENs were detected [30]. Most of these mutations cause AD [30]. At least one confers on its carriers protection from both conventional AD and aging-associated cognitive decline, AACD [31,32]. All of them affect only the dynamics, structure, and metabolism of Aβ. The notion of the centrality of Aβ in Alzheimer’s disease and the assertion of the irrelevance of extracellular Aβ in AD, ostensibly irreconcilable, are nevertheless not incompatible. What is excluded from the position of centrality is extracellular Aβ. Importantly, there is another physiologically occurring population of Aβ, namely intraneuronal Aβ (*i*Aβ). It follows that it is this class of Aβ, *i*Aβ, which assumes the position of centrality in AD, a notion strongly supported by the observations that there is a good correlation between the levels of *i*Aβ and the occurrence of the disease [33,34,35,36,37,38,39,40,41,42,43,44,45].

## 4. Intraneuronal Aβ and AD: Amyloid Cascade Hypothesis 2.0 (ACH2.0)

The preceding sections contend that the ACH has outlived its usefulness and has to be replaced. They also impose certain restrictions: any new theory of conventional Alzheimer’s disease must retain the centrality of Aβ, more precisely of *i*Aβ. The ACH2.0 incorporated this restriction as one of its principal tenets [1,2,3,4,5,6,7,8,9]. Moreover, it posited that the driver of the disease is Aβ generated not by the proteolysis of AβPP but in the AβPP-independent pathway. Furthermore, it asserted that the bulk or the entire output of Aβ produced independently of AβPP is not secreted but is retained intraneuronally as *i*Aβ. These two key principles of the ACH2.0 are based on the empirical data obtained in clinical trials of potential AD drugs [15,16]. They are discussed in detail elsewhere [1,4] and can be briefly summarized as follows. First, the substantial depletion of extracellular Aβ did not impede in any way the progression of AD at its symptomatic stages. It follows that AD is driven by *i*Aβ. Second, the suppression of the proteolytic cleavage of AβPP by BACE1 and, consequently, a substantial inhibition of the production of Aβ had no beneficial effect on the progression of the disease, implying that AD pathology-driving *i*Aβ is produced in the AβPP-independent pathway [1,2,3,4,5,6,7,8,9].

The principles of the ACH2.0 formulated above make it apparent that the AβPP-independent pathway that generates *i*Aβ is the essence and the active core of Alzheimer’s disease. As discussed in more detail in the following sections below, this pathway is activated by the neuronal integrated stress response (ISR), which supplies the essential components not present under the regular (non-ISR) conditions; upon its initiation, the AβPP-independent *i*Aβ production pathway becomes self-sustainable: its product propagates the ISR conditions and thus perpetuates the operation of the pathway and the progression of the disease (on the alternative possibility that the neuronal ISR is propagated and disease is driven not by *i*Aβ but rather by C99 produced independently from AβPP, see the end-note in Section 15 and the follow-up discussion). Alzheimer’s disease could be of two types, designated “conventional” and “unconventional”. These types are defined by the manner in which the neuronal ISR is elicited [8,9]. In conventional Alzheimer’s disease, the neuronal ISR is triggered by AβPP-derived *i*Aβ at the levels exceeding the “critical threshold” (further discussed below). In contrast, in unconventional Alzheimer’s disease, the neuronal ISR is triggered by any competent stressor other than AβPP-derived *i*Aβ [8,9]. The separation of Alzheimer’s disease into two types is important not only for its categorization and systematization but also practically. This is because the potential therapeutic strategies operate in distinctly different AD type-specific manners. These strategies and their application in conventional and unconventional AD are discussed below.

## 5. In Conventional AD, *i*Aβ Is the Triggering Agent of the Disease: Two Physiologically Operating Sources of Intraneuronal AβPP-Derived Aβ

As discussed in the preceding section, the elicitation of the neuronal ISR in conventional AD is mediated, and, consequently, the disease is initiated by AβPP-derived *i*Aβ at the levels exceeding the critical threshold. In this sense, AβPP-derived *i*Aβ is the triggering agent of conventional AD. It should be emphasized, however, that the accumulation of AβPP-derived *i*Aβ is a normal physiological process that occurs in both “normal” individuals (i.e., those who don’t develop AD within their lifetimes) and future AD sufferers. What distinguishes the two are parameters defining the timing of the crossing of the critical threshold by AβPP-derived *i*Aβ (further discussed below).

The accumulation of AβPP-derived *i*Aβ occurs via two physiologically operating mechanisms. One is the intraneuronal retention of a fraction of the output of Aβ generated by the proteolysis of AβPP. Proteolytic generation of Aβ requires two cleavages of its precursor, AβPP. The first cleavage is effected by β-site AβPP cleaving enzyme (BACE, also known as β-secretase)). The product of interest generated by this cleavage is the 99-amino-acid-residues-long C-terminal fragment (CTF) of AβPP. This fragment is designated as C99; it starts with the Asp672, which constitutes the N-terminus of both C99 and Aβ. γ-secretase (a PSENs-containing complex) performs the second cleavage within a narrow internal segment of C99; this generates Aβ, which is typically 40 or 42 amino acid residues long. Both β- and γ-secretase cleavages occur on cellular membranes. The latter usually takes place on the plasma membrane and is followed by secretion of newly generated Aβ. However, a fraction of γ-secretase cleavages occurs on intracellular membranes associated with various organelles [46,47,48,49,50,51,52,53,54]. Aβ generated in such a manner is not secreted to the extracellular space but is retained intraneuronally. As discussed below, certain FAD-causing mutations of AβPP or PSEN exert their effects by increasing the frequency of γ-secretase cleavages on the intraneuronal membranes.

Another physiologically operating mechanism underlying the accumulation of AβPP-derived *i*Aβ is the cellular uptake, i.e., the internalization of secreted Aβ. This mechanism converts, de facto, a fraction of Aβ (i.e., secreted Aβ) into *i*Aβ [55,56,57,58,59,60]. The internalization of secreted Aβ was shown to require the aggregation of the latter via its oligomerization [59,60]. Aβ42 isoform is taken up twice as efficiently as another major Aβ isoform, Aβ40. The reason for this is the demonstrated propensity of Aβ42 to aggregate [60]. This feature of Aβ42 explains its significantly increased cellular toxicity (in comparison with other Aβ isoforms): not only are its intracellular aggregates toxic, but its enhanced capacity to internalize significantly (and disproportionally) also increases its intracellular concentration. It should be mentioned that not only neurons but also other cell types are capable of internalizing extracellular Aβ; this cellular uptake is mediated by a variety of cellular receptors [61,62,63,64,65,66,67,68,69].

## 6. AβPP-Derived *i*Aβ Is Incapable of Reaching AD Pathology-Causing Levels 

**Figure 1:**
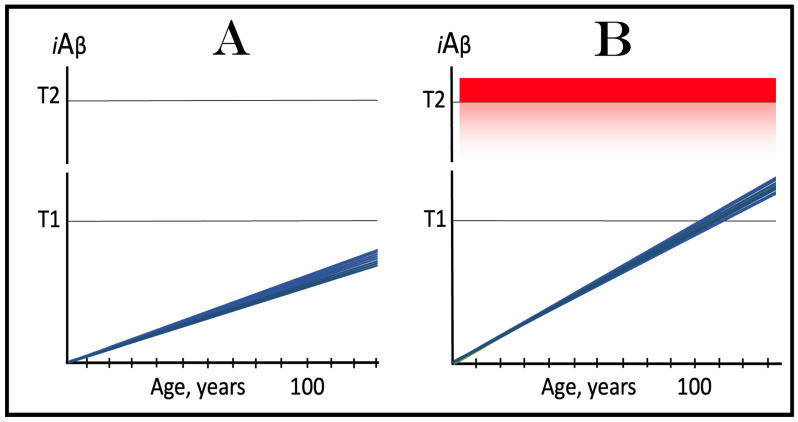
**AβPP-derived *i*Aβ cannot reach the AD pathology-causing range in human neurons**. *i*Aβ: intraneuronal Aβ. *Blue lines*: Levels of *i*Aβ in individual neurons. ***T*1** *threshold*: Cellular concentration of *i*Aβ that triggers the elicitation of the neuronal integrated stress response. ***T*2** *threshold*: Levels of *i*Aβ that trigger apoptosis or necroptosis of neuronal cells. *Gradient-pink box*: Range of concentrations of *i*Aβ that cause and propel AD pathology. *Red box*: Apoptotic zone of *i*Aβ concentrations; within this zone the neurons have either undergone apoptosis or are dead. (**A**) The accumulation of AβPP-derived *i*Aβ is an exceedingly slow process; in the majority of humans, it neither reaches nor crosses the T1 threshold within their lifetime. (**B**) Provided the accumulation of *i*Aβ continues at the constant linear rate following the crossing of the T1 threshold, it cannot possibly reach the concentrations that cause and drive AD pathology and propel the progression of the disease.

Within the framework of the ACH2.0, AD cannot occur in the absence of the operational AβPP-independent *i*Aβ generation pathway [4,8]. This is because the rate of accumulation of *i*Aβ produced independently of AβPP is apparently orders of magnitude greater than that of *i*Aβ generated by the proteolysis of AβPP (further discussed below). AβPP-derived *i*Aβ is simply incapable of reaching AD pathology-causing levels. This point is illustrated in Figure 1. In this figure, the T1 threshold designates “critical” neuronal ISR-eliciting levels of *i*Aβ in individual neurons. The AD pathology-causing range of *i*Aβ concentrations starts way above the T1 threshold and is represented by a gradient-pink box. The T2 threshold denotes *i*Aβ levels triggering the entrance into the neuronal death program, and the red box depicts the “apoptotic zone” of *i*Aβ concentrations. As discussed below, in the majority of humans, AβPP-derived *i*Aβ neither reaches nor crosses the T1 threshold within their lifetime (panel A of Figure 1). This implies that the accumulation of AβPP-derived *i*Aβ is an exceedingly slow process and even if it crosses the T1 threshold within the lifetime of an individual (panel B of Figure 1), were its accumulation to continue at the same rate following the T1 crossing as prior to it, it cannot possibly reach the concentrations that cause and drive AD pathology and propel the progression of the disease.

## 7. If and When Accumulated over the Critical Threshold, AβPP-Derived *i*Aβ Triggers the Elicitation of the Neuronal Integrated Stress Response

As was mentioned above, the accumulation of AβPP-derived *i*Aβ occurs physiologically in every individual. In the majority of the human population, it does not reach the T1 threshold within the lifetime of an individual. However, if and when the T1 is crossed by AβPP-derived *i*Aβ, it triggers a chain of events culminating in the elicitation of the neuronal integrated stress response. In any chain of events leading to the elicitation of the ISR, the penultimate event immediately preceding and triggering the ISR state is the site-specific phosphorylation of the eukaryotic translation initiation factor 2α, eIF2α, at its Ser51 residue. This phosphorylation can be executed by any member of the family of eIF2α kinases, which comprises protein kinase double-stranded RNA-dependent (PKR), PKR-like endoplasmic reticulum (ER) kinase (PERK), general control non-derepressible-2 kinase (GCN2), and heme-regulated inhibitor kinase (HRI). In conventional AD-affected neurons, eIF2α is phosphorylated by PKR and/or HRI. The activation of both kinases in conventional AD is mediated by AβPP-derived *i*Aβ accumulating over the critical threshold (designated the T1 threshold). Thus, the activation of PKR was detected in cell models that overexpress Aβ [70,71,72]. Moreover, neurons of AD patients were shown to contain activated PKR along with phosphorylated eIF2α [73,74]. In the activation of PKR triggered by the over-T1 levels of *i*Aβ, the kinase is apparently phosphorylated and activated by the PKR activator (designated PACT), as suggested by the co-localization of PACT and activated PKR in the neurons of AD patients [75]. An alternative pathway of *i*Aβ-triggered activation of PKR involves TNFα, as was observed in mouse models overexpressing Aβ [76].

As for the HRI kinase, it is activated in AD as a result of mitochondrial dysfunction. *iAβ*-triggered mitochondrial distress is one of the earliest symptoms of Alzheimer’s disease and was investigated extensively [77,78,79,80,81,82,83,84,85,86,87,88,89,90,91,92,93,94]. These studies yielded the understanding of the pathway leading from *i*Aβ to the activated HRI kinase via mitochondrial distress. One of the early effects of mitochondrial dysfunction is the activation of mitochondrial protease OMA1 [95,96]. Among the targets of the activated OMA1 protease is another mitochondrial protein, DELE1, which is consequently cleaved in a site-specific fashion [95,96]. One of the DELE1 fragments resulting from the OMA1 cleavage migrates to the cytosol. There, it binds to HRI, thus activating it [95,96]. This pathway was shown to operate in a variety of cell types including neurons [95]. Thus, *i*Aβ, accumulated to over-T1 levels, triggers the activation of PKR or HRI, or of both kinases. Consequently, eIF2α is phosphorylated at its Ser51 residue. This, in turn, elicits the neuronal ISR.

## 8. AD Commences with the Activation of the AβPP-Independent *i*Aβ Production Pathway: Pivotal Role of the Neuronal ISR 

As discussed above, the rate of generation of *i*Aβ in the AβPP proteolytic pathway is insufficient to support its accumulation to the AD pathology-causing levels. This occurs only when the AβPP-independent *i*Aβ production pathway is activated [1,4]. Therefore, AD commences concurrently with and as a consequence of the activation of the AβPP-independent *i*Aβ generation pathway, which is enacted by the neuronal integrated stress response. The cellular ISR is a crucial signaling pathway and a survival mechanism that is preserved evolutionarily and elicited by a multitude of stressors, pathological as well as environmental. Thus, the ISR-eliciting stressors stem from protein aggregation and/or its incorrect folding. They result from inflammation and both viral and bacterial infections [97,98,99,100,101,102,103,104,105,106]. Their occurrence follows imbalances in protein homeostasis and nutritional deficiencies. The definition “integrated” in “ISR” conveys the fact that, regardless of the nature and origin of various stressors, their effects converge on the same singular event, the phosphorylation of eIF2α at its residue Ser51, which is enacted by any member of a limited set of eIF2α kinases consisting of only four enzymes: PKR, PERK, GCN2, and HRI. This event commences the integrated stress response. Because a principal attribute (active eIF2α) of the fundamental cellular process (translation) is affected, the manifestations of the cellular ISR are drastic. Transcriptional and translational landscapes are profoundly modified and both processes are radically reprogrammed. A defined set of transcription factors are activated and the cellular gene expression pattern is altered. The global cap-dependent cellular protein synthesis is severely suppressed. Crucially, the cap-independent production of a small and selected subset of protein species is activated.

The ACH2.0 asserts that this subset of proteins translated in the cap-independent manner under the neuronal ISR conditions and not occurring in the non-ISR cellular state contains critical components that are necessary and sufficient for the operation of the AβPP-independent *i*Aβ generation pathway [1,4]. Several defined molecular mechanisms potentially enabling the production of *i*Aβ independently of AβPP are described below and elsewhere [4,7]. Here, it suffices to state that in all of these mechanisms, the bulk or, possibly, the entire output of the resulting Aβ is retained intraneuronally as *i*Aβ. For this and other reasons (further discussed below), the rate of accumulation of *i*Aβ produced in the AβPP-independent pathway is orders of magnitude greater than that derived by the AβPP proteolysis. As a result, with the AβPP-independent pathway being operational, its *i*Aβ product rapidly accumulates, reaches the AD pathology-causing range, and drives the disease. It follows from the above discourse that whereas the AβPP-independent *i*Aβ production pathway is the driver of Alzheimer’s disease, the neuronal ISR is the essence, the enabler of AD, pivotal in the development of the disease. In this context, AD IS A DISEASE OF THE NEURONAL ISR. Furthermore, it also follows that Alzheimer’s disease can be triggered through the sustainable elicitation of the neuronal ISR by any stressor. The elicitation of the neuronal ISR by AβPP-derived *i*Aβ is only one special (albeit possibly predominant) case of AD that we refer to as “conventional AD” [8,9]. However, AD can also be triggered by the neuronal ISR elicited by various stressors distinct from AβPP-derived *i*Aβ. These cases are collectively referred to by us as “unconventional AD” [8]. The parameters of their development and progression may differ from those of conventional AD cases; moreover, these parameters could be specific for a type of stressor that elicits the neuronal ISR. Nevertheless, these cases are triggered by the neuronal ISR and driven by the AβPP-derived *i*Aβ generation pathway; they are therefore, unmistakably, cases of Alzheimer’s disease. We propose that the following operational definition of AD is adopted: ALZHEIMER’S IS A DISEASE TRIGGERED BY THE NEURONAL INTEGRATED STRESS RESPONSE ELICITED BY THE AβPP-DERIVED *i*Aβ OR OTHERWISE AND DRIVEN BY *i*Aβ (possibly by C99; see below) PRODUCED IN THE AβPP-INDEPENDENT PATHWAY.

## 9. *i*Aβ Produced Independently of AβPP Drives Alzheimer’s Disease, Propagates the Neuronal ISR State and Perpetuates Its Own Generation

As discussed in the preceding section, the neuronal integrated stress response enables the operation of the AβPP-independent *i*Aβ generation pathway. In conventional AD, the elicitation of the neuronal ISR is triggered by the accumulation of AβPP-derived *i*Aβ over the critical T1 threshold. At these levels, AβPP-derived *i*Aβ mediates, either through PACT or TNFα, the activation of the PKR kinase, or, via the mitochondrial dysfunction and OMA1 to DELE1 signaling pathway, the phosphorylation and thus activation of the HRI kinase. When activated, either PKR or HRI or both phosphorylate eIF2α at its Ser51 residue, and the neuronal ISR ensues. The neuronal ISR enables the activity of the AβPP-independent *i*Aβ production pathway by providing its essential components that are not present in cells under the regular, non-ISR, conditions. The entire output of Aβ produced in this pathway is retained intraneuronally as *i*Aβ. Consequently, it rapidly accumulates to the AD pathology-driving levels, triggers the formation of neurofibrillary tangles [107,108,109,110], and eventually causes neuronal loss either through apoptosis or necroptosis [111]. It follows that the AβPP-independent *i*Aβ generation pathway is the active core of Alzheimer’s disease. Since, as discussed above, AβPP-derived *i*Aβ cannot attain AD pathology-causing levels, the disease cannot occur unless this pathway is operational.

**Figure 2:**
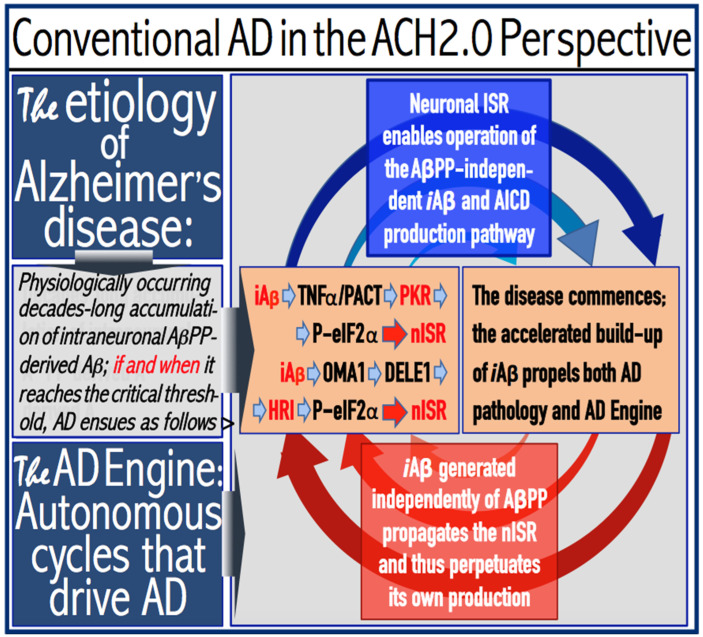
**Conventional AD in the ACH2.0 perspective**. *i*Aβ: Intraneuronal Aβ. *eIF2α:* Eukaryotic translation initiation factor 2*α*. *PKR and HRI:* Kinases capable, when activated, of phosphorylating eIF2α. *TNFα*: Tumor necrosis factor α; potentially capable of activating PKR. *PACT*: PKR activator. *OMA1:* Mitochondrial distress-activated mitochondrial protease. *DELE1:* Substrate of OMA1; its cleavage leads to the activation of HRI. *nISR:* Neuronal integrated stress response; it is elicited by phosphorylation of eIF2α and enables the generation of components needed for the operation of the AβPP-independent pathway of *i*Aβ production. *AICD:* AβPP intracellular domain; results from the processing of C99. AβPP-derived *i*Aβ accrues physiologically in a life-long process. In most individuals, it does not reach the T1 threshold and no AD occurs. When the T1 threshold is crossed, PKR and/or HRI kinases are activated, eIF2α is phosphorylated, the ISR is elicited, the operation of the AβPP-independent *i*Aβ generation pathway is initiated, and the disease commences. The bulk if not the entire Aβ output of the AβPP-independent *i*Aβ generation pathway are retained as *i*Aβ. Its rapid accumulation both drives AD pathology and, by sustaining the activity of PKR and/or HRI, propagates the neuronal ISR and, consequently, perpetuates its own AβPP-independent generation. This cycle, denoted by the arched red and blue arrows, constitutes the driver of AD, the “AD Engine”.

Commencing and driving the progression of Alzheimer’s disease is one of the two principal functions of *i*Aβ produced in the AβPP-independent pathway. Its second principal function in AD is the sustained propagation of the neuronal integrated stress response and, consequently, the perpetuation of the operation of the AβPP-independent *i*Aβ generation pathway. The continuous activity of this pathway requires uninterrupted maintenance of the neuronal ISR conditions. The cessation of the ISR state would stop the supply of the essential components of the AβPP-independent *i*Aβ production pathway and thus disable it. In conventional AD, the neuronal ISR and the activity of the AβPP-independent *i*Aβ production pathway are triggered by AβPP-derived *i*Aβ at the above-T1 levels. The production of *i*Aβ independently of AβPP and its consequent rapid accumulation ensure that its levels are sustainably maintained well above the T1 threshold. As a result, *i*Aβ generated independently of AβPP continuously propagates the neuronal ISR state and thus ensures its own sustained production; the AβPP-independent production pathway is rendered self-sustainable and its activity is perpetuated. The self-sustainability aspect of the AβPP-independent production of *i*Aβ, which de facto constitutes the “AD Engine”, is illustrated in Figure 2. Note that this figure adequately depicts conventional AD. The dynamics of *i*Aβ produced independently of AβPP in unconventional AD is different and, as described in Section 44, Section 45 and Section 46 below, necessitates a lag period for the AβPP-independent *i*Aβ generation pathway to attain self-sustainability.

## 10. Dynamics of Accumulation of AβPP-Derived *i*Aβ Plays a Decisive Role in the Occurrence and Timing of Conventional AD

**Figure 3:**
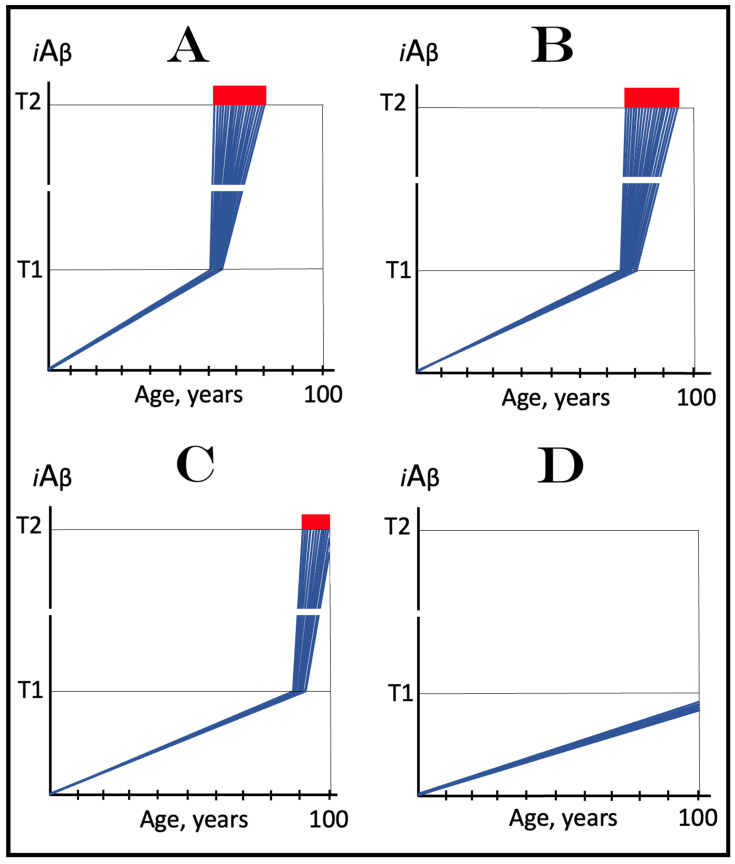
**Dynamics of AβPP-derived *i*Aβ accumulation determines the occurrence and timing of conventional AD**. *i*Aβ: Intraneuronal Aβ. *Blue lines*: Levels of *i*Aβ in individual neurons. ***T*1** *threshold*: Cellular concentration of *i*Aβ that triggers the elicitation of the neuronal integrated stress response. ***T*2** *threshold*: Levels of *i*Aβ that trigger apoptosis or necroptosis of neuronal cells. *Red box*: Apoptotic zone; within this zone, the neurons have either undergone apoptosis or are dead. With the exception of the rate of accumulation of AβPP-derived *i*Aβ, all parameters are considered constant. In these terms, the occurrence of AD is a function of and its timing is inversely proportional to the rate of accumulation of AβPP-derived *i*Aβ. (**A**) The rate of accumulation of AβPP-derived *i*Aβ is such that the T1 threshold is crossed and AD commences in the patient’s mid-sixties, statistically the age of onset of sporadic AD. In (**B**), the rate of accumulation of AβPP-derived *i*Aβ decreases and the age of the T1 crossing and, consequently, of the onset of AD increases to the patient’s eighties. In (**C**), the rate of accumulation decreases further and the T1 threshold is crossed and the disease occurs in the patient’s nineties. (**D**) The rate of accumulation of AβPP-derived *i*Aβ is such that the T1 threshold is not reached within the lifetime of the individual; neither is the T1 threshold crossed nor does conventional AD occur.

As discussed above, within the framework of the ACH2.0, conventional AD commences only following the crossing of the T1 threshold by AβPP-derived *i*Aβ. This is what triggers the activation of PKR and/or HRI and initiates the chain of cellular events culminating in the activation of the AβPP-independent iAβ generation pathway. It follows that the occurrence of conventional AD is a function of the rate of accumulation of AβPP-derived *i*Aβ. If the rate of the accumulation of AβPP-derived *i*Aβ is such that it does not reach the T1 threshold within the lifetime of the individual, no conventional AD would occur. In cases where AβPP-derived *i*Aβ reaches and crosses the T1 threshold, conventional AD is initiated. In these cases, the timing of the commencement of the disease is also a function of the rate of accumulation of AβPP-derived *i*Aβ. The timing of the occurrence of conventional AD is, in fact, inversely proportional to the rate of accumulation of AβPP-derived *i*Aβ. The greater the rate of AβPP-derived *i*Aβ accumulation, the shorter the timing of the commencement of conventional AD. The relationships between the occurrence and timing of conventional AD and the rate of accumulation of AβPP-derived *i*Aβ are illustrated in Figure 3. In this figure, the only variable is the rate of accumulation of AβPP-derived *i*Aβ. In panel A, this rate is such that the T1 threshold is crossed by AβPP-derived *i*Aβ when the individual is in their mid-sixties, which is statistically the age of onset of sporadic AD. With the decrease in the rate of accumulation, the timing of the crossing of the T1 threshold by AβPP-derived *i*Aβ increases. In panel B, it occurs in the patient’s eighties, and in panel C, where the rate of accumulation decreases further, the T1 crossing occurs in the patient’s nineties. Finally, panel D in Figure 3 reflects the situation observed in the majority of individuals: the rate of accumulation of AβPP-derived *i*Aβ is such that the T1 threshold is not reached within the lifetime of the individual. Consequently, neither is the T1 threshold crossed nor does conventional AD occur. The T1 threshold is, thus, literally a thin line separating health and AD.

## 11. Validation of the Concept: All Conventional AD-Related Mutations Act via Altering the Rate of Accumulation of AβPP-Derived *i*Aβ in a Predicted Manner

The concept connecting the rate of accumulation of AβPP-derived *i*Aβ with the occurrence of conventional AD and with the timing of the commencement of the disease allows us to make two well-defined predictions. One is that the augmentation of the rate of accumulation of AβPP-derived *i*Aβ would trigger AD or at least hasten its onset. Another, complementary prediction is that the reduction in the rate of accumulation of AβPP-derived *i*Aβ would delay or prevent the occurrence of conventional AD. Both predictions are verifiable, and the simplest approach to evaluate them is to analyze the mode of operation of the known mutations and factors that either cause or predispose to AD or protect from the disease. The results of such evaluations are extraordinary. Without a single exception, all known mutations causing the early onset of conventional AD and factors that predispose to the conventional form of the disease augment the rate of accumulation of AβPP-derived *i*Aβ. On the other hand, the mutation that confers on its carriers considerable protection from conventional AD and AACD (aging-associated cognitive decline) reduces the rate of accumulation of AβPP-derived *i*Aβ.

A typical example is the mode of operation of ApoE4, which predisposes its carriers to AD in a dose-dependent manner, i.e., carriers of two copies of the ApoE4 gene are much more likely to develop AD than individuals carrying only one copy. Whereas all variants of ApoE facilitate cellular uptake of secreted Aβ, the efficiency of ApoE4 in the importation of extracellular Aβ is significantly higher than that of other ApoE isoforms [38]. The increased efficiency of ApoE4 in the cellular uptake of secreted Aβ translates into the increased rate of influx of AβPP-derived *i*Aβ. This, in turn, increases the rate of accumulation of the latter, decreases the timing of the T1 crossing, and thus accelerates the occurrence of AD.

In another example, a number of AβPP and PSEN FAD-causing mutations facilitate γ-cleavage at position 42 of Aβ [57]. This results in the augmented production and secretion of the Aβ42 isoform. This isoform of Aβ is repatriated, i.e., imported inside the cell from the extracellular space at the rate, which is two times greater (due to its propensity to aggregate) than the rate of importation of Aβ40, another major Aβ isoform [56,57,58]. Each of these mutations substantially increases the rate of the influx of AβPP-derived *i*Aβ, augments the rate of its accumulation, reduces the timing of the crossing of the T1 threshold, and thus causes the early onset of AD [56,57,58].

Yet another example is a category of FAD mutations that augment the fraction of γ-cleavages, which take place on intracellular membranes associated with cellular organelles rather than on plasma membranes. This category consists of various PSEN mutations [112] and also includes the Swedish Aβ mutation [113]. Aβ generated by γ-cleavages that occur on the intraneuronal membranes is not secreted but remains within neuronal cells. Therefore, the increase in the proportion of γ-cleavages occurring on intracellular membranes augments the rate of the influx of AβPP-derived *i*Aβ, which, in turn, elevates the rate of its accumulation, accelerates the crossing of the T1 threshold, and thus triggers FAD [112,113]. 

The intraneuronal processing of AβPP, C99, and *i*Aβ includes physiologically occurring cleavages within *i*Aβ as well as within Aβ segments of AβPP and C99. The activities that carry out these cleavages (discussed in more detail below) are associated with BACE1 and BACE2 and apparently regulate the intraneuronal levels of Aβ. The activity associated with BACE2 cleaves *i*Aβ or the Aβ segment of its precursor molecules at two Aβ sites, Aβ19 and Aβ20. The Flemish FAD mutation, which occurs at the position Aβ21, significantly reduces the rate of these intra-Aβ cleavages [114]. By inhibiting cleavages within the Aβ segment of its precursor molecules, this mutation increases the production of Aβ and the influx of *i*Aβ, and by inhibiting cleavages within *i*Aβ, the mutation decreases its efflux. Cumulatively, these events augment the rate of accumulation of AβPP-derived *i*Aβ, accelerate the crossing of the T1 threshold, and trigger the early onset of AD [114]. 

In similarity to the Flemish Aβ mutation, the Icelandic Aβ mutation also affects the intra-Aβ cleavage, in this case by the physiologically operating activity associated with BACE1. This activity cleaves at the position Aβ10 within either *i*Aβ or the Aβ segment of its precursor molecules (AβPP and C99) [31,32]. The Icelandic mutation increases the rate of the intra-Aβ cleavage by the BACE1-associated activity. By increasing the rate of intra-Aβ cleavage within the Aβ segment of its precursors, this mutation decreases the rate of the production of Aβ, and, correspondingly, of its cellular re-entry as *i*Aβ, and by increasing the efficiency of intra-Aβ cleavage within *i*Aβ, it augments its efflux. Cumulatively, these processes decrease the rate of accumulation of the AβPP-derived *i*Aβ. The extent of the increase in the rate of intra-Aβ cleavage at the Aβ10 site (known as β’-site), associated with BACE1, is only about 30% [31]. The resulting reduction in the rate of accumulation of AβPP-derived *i*Aβ, however, is apparently sufficient for the protection from AD and AACD observed in the carriers of the Icelandic mutation. 

Taken together, the above observations and considerations constitute a compelling validation of the notion that both the occurrence and the timing of the commencement of conventional Alzheimer’s disease are a function of the rate of accumulation of AβPP-derived *i*Aβ. 

## 12. Conventional AD Is a Two-Stage Disease, but Stage One Becomes Such Only Retroactively

As depicted in Figure 3, panel D, the kinetics of the accumulation of AβPP-derived *i*Aβ in healthy individuals is single-phased. AβPP-derived *i*Aβ is accrued throughout the individual’s life but does not reach the T1 threshold. In contrast, the kinetics of the accrual of *i*Aβ in conventional Alzheimer’s disease is evidently double-phased. The disease occurs in its entirety, from the commencement until the end stage, within the second phase of *i*Aβ accumulation. However, without the completion of the first phase, i.e., the crossing of the T1 threshold, the second phase is not possible. Therefore, the first phase of the accumulation of AβPP-derived *i*Aβ is the integral component of the disease. Accordingly, the two phases of the accrual of *i*Aβ correspond to the two stages of conventional AD. In the first stage of the disease, *i*Aβ is produced in the AβPP proteolytic pathway and accrues through the importation (cellular re-entry) of a portion of secreted extracellular Aβ and via the retention of *i*Aβ generated by γ-cleavages of C99 on the intracellular membranes. When AβPP-derived *i*Aβ reaches the T1 threshold, it mediates the activation of the PKR and/or HRI kinases. Consequently, eIF2α is phosphorylated at its Ser51 residue, the neuronal ISR is elicited, and the AβPP-independent *i*Aβ generation pathway is initiated. Only at this point does AD commence, and its progression is driven by *i*Aβ produced independently of AβPP (or, as discussed below, by C99 generated independently of AβPP).

Despite the above considerations, naming the first phase of *i*Aβ accrual as the first stage of AD is paradoxical. This is because the accrual of AβPP-derived *i*Aβ is a physiological life-long process. AD does not occur during this phase, and in most individuals, AβPP-derived *i*Aβ never reaches the T1 threshold during their lifetimes. Thus, the designation of the first phase of the accrual of *i*Aβ as the first stage of AD is based solely on its potential to reach the T1 threshold and trigger the disease; it is, in other words, a potential enabler of the disease. It follows, therefore, that the first phase of *i*Aβ accumulation becomes the first stage of AD only retrospectively, i.e., only if and when the disease actually occurs. Short of this, the life-long accrual of AβPP-derived *i*Aβ is just another normal physiological occurrence.

## 13. Prevalence of Conventional AD Is a Function of Longevity: Eventual Inevitability of the Disease Unless Treated Preventively

In the preceding sections, we have established that within a defined lifespan, the occurrence of conventional AD is a function of the rate of accumulation of AβPP-derived *i*Aβ. The key words in the previous sentence are “a defined lifespan”. Indeed, consider again Figure 3. In this figure, the lifespan is set at 100 years. As depicted in panel D, AβPP-derived *i*Aβ does not reach the T1 threshold within this time period and, consequently, no AD occurs; however, both the T1 crossing and AD would occur if the lifespan were set at 120 years. In panels A-C, AβPP-derived *i*Aβ crosses the T1 threshold with the timing proportional to the rate of its accumulation and AD is triggered. However, if the lifespan were set at 80 years, no AD would occur in panel C. If the lifespan were set at 70 years, no disease would occur in panel B. If, finally, the lifespan were defined as 50 years, no AD would be triggered in panel A of Figure 3. Fifty years was actually the life expectancy in Germany at the time of Dr. Alois Alzheimer, after whom the disease is named. In fact, Dr. Alzheimer died at age 51 in 1915. When he described AD in 1906, this disease was exceedingly rare because of the lifespan limitation. The human lifespan has been steadily increasing ever since, and so has the prevalence of AD. Currently, this disease is not a rarity but rather a common occurrence. The incidence of AD is around 5% at 65, 15% at 75, and 35% at 85. As an extrapolation, it can be stated with a substantial certainty that, given a sufficient lifespan, Alzheimer’s disease is inevitable. Provided that the life expectancy continues to increase, the occurrence of AD is bound to become an eventuality. This tendency could be counteracted in two ways, limiting the lifespan or developing effective preventive and curative AD drugs. Needless to say, the latter option is preferable; it is addressed below and elsewhere [6,9].

## 14. The Occurrence and Timing of AD and the Incidence of AACD Are Directly Proportional to the Extent of the Neuronal ISR-Triggering T1 Threshold

**Figure 4:**
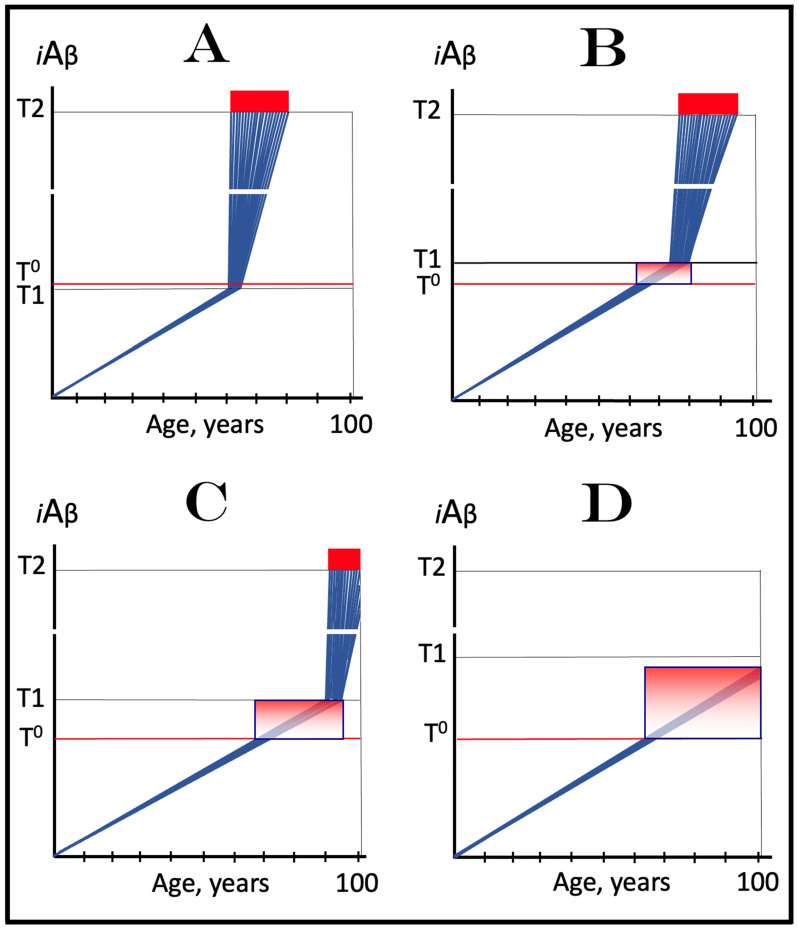
**The occurrence and timing of AD and the incidence of AACD are directly proportional to the extent of the T1 threshold**. *i*Aβ: Intraneuronal Aβ. *Blue lines*: Levels of *i*Aβ in individual neurons. ***T*^0^** *threshold*: The level of AβPP-derived *i*Aβ above which the neuronal damage manifesting as AACD commences. ***T*1** *threshold*: Cellular concentration of *i*Aβ that triggers the elicitation of the neuronal integrated stress response. ***T*2** *threshold*: Levels of *i*Aβ that trigger apoptosis or necroptosis of neuronal cells. *Red box*: Apoptotic zone; within this zone, the neurons have either undergone apoptosis or are dead. *Gradient-pink boxes*: The range of AβPP-derived *i*Aβ concentrations between the T^0^ and T1 thresholds (provided T^0^ < T1), a zone of the occurrence of AACD. All parameters (including the rate of accumulation of AβPP-derived *i*Aβ and the extent of the T^0^ threshold), with the exception of the extent of the T1 threshold, are considered constant. (**A**) The extent of the T1 threshold is smaller than that of the T^0^ threshold, such that the T1 crossing occurs and AD commences at the age of sixty; no AACD occurs. (**B**) The extent of the T1 threshold increases and is greater than that of the T^0^ threshold. AACD commences when the T^0^ is reached and morphs into AD with the T1 crossing at about 75 years. (**C**) The T1 threshold is elevated further. AACD commences at the same time as in panel B but the T1 crossing and the commencement of AD occur later, at about ninety years. (**D**) The extent of the T1 threshold is elevated further; neither is it crossed nor does AD occur. AACD commences at precisely the same time as in (**B**,**C**) and persists for the remaining lifespan.

The rate of accumulation of AβPP-derived *i*Aβ is only one of the two parameters that determine the occurrence and the timing of the commencement of AD. The second parameter is the extent of the neuronal ISR-triggering T1 threshold. Conceptually, the variability of the extent of the T1 threshold is instrumental not only in understanding the development of AD but also in defining the nature and requirements of aging-associated cognitive decline, AACD. It is apparent that at any given rate of the accrual of AβPP-derived *i*Aβ, the timing of the crossing of the T1 threshold would be directly proportional to the extent of the latter: the greater the extent of the T1 threshold, the longer it would take for AβPP-derived *i*Aβ to cross it. This dynamic is illustrated in Figure 4. In this figure, with one exception, all parameters, including the rate of accumulation of AβPP-derived *i*Aβ, are constant. The only variable is the extent of the neuronal ISR-triggering, AβPP-independent *i*Aβ production pathway-activating T1 threshold. In panel A of Figure 4, the extent of the T1 threshold is set at the level below the range of neuronal damage-causing concentrations of *i*Aβ. At the shown setting, AβPP-derived *i*Aβ reaches the T1 threshold and thus triggers the elicitation of the neuronal ISR and the activation of the AβPP-independent *i*Aβ production pathway at the age of 60. As the extent of the T1 threshold is elevated, the timing of its crossing increases accordingly. In Panel B, the T1 threshold is crossed, neuronal ISR is elicited, the AβPP-independent *i*Aβ generation pathway is activated, and AD commences at 75. In panel C, with an even greater extent of T1, all this occurs at the age of 90. When the extent of the T1 threshold increases further, as shown in panel D of Figure 4, neither is it crossed nor does AD occur within the lifetime of the individual.

It is apparent from the above considerations that elevated extents of the T1 threshold confer significant benefits in that they delay or even prevent the T1 crossing, consequently delaying or preventing AD. These benefits are not without a price: increased extents of the T1 threshold enable the occurrence of AACD. As the extent of the T1 threshold increases, it eventually and inevitably reaches the range of neuronal damage-causing concentrations of *i*Aβ. ACH2.0 defines AACD as the manifestation of the neuronal damage caused by AβPP-derived *i*Aβ at the levels below the T1 threshold [4]. Moreover, it defines the lower boundary of the range of neuronal damage-causing concentrations of AβPP-derived *i*Aβ as the T^0^ threshold. In these terms, AACD is a condition occurring at the range of AβPP-derived *i*Aβ concentrations between the T^0^ and T1 thresholds. For AACD to occur, the extent of the T^0^ has to be lower than the extent of the T1 threshold; following the T1 crossing, the elicitation of the neuronal ISR, and the activation of the AβPP-independent *i*Aβ production pathway, AACD morphs into AD [4]. In panel A of Figure 4, the extent of the T^0^ threshold is higher than that of the T1. No AACD occurs in this scenario. In panel B of Figure 4, the AACD stage commences when the T^0^ threshold is crossed and evolves into AD with the T1 crossing. In panel C of Figure 4, with an even greater extent of the T1 threshold, the duration of AACD increases accordingly. In panel D of Figure 4, the T1 threshold is not reached during the lifetime of the individual. Consequently, no AD occurs but the AACD condition commences and persists for the remaining portion of the lifespan.

The age of the onset of AACD is statistically greater than that of late-onset (sporadic) AD [115,116]. On the other hand, AACD occurs in a much larger portion of the population than sporadic AD. This indicates that in the majority of the population, the extents of both the T^0^ and T1 thresholds are greater than the extent of the T1 threshold in sub-populations susceptible to sporadic AD. In other words, individuals susceptible to sporadic AD constitute sub-populations defined by a low extent of the T1 threshold, and it is the latter which predisposes them to the disease. Taking into account the dependency of the T1 crossing and the occurrence of AD on the rate of accumulation of AβPP-derived *i*Aβ discussed above, it can be posited that sub-populations susceptible to AD are not random but rather consist of individuals exhibiting a lower than average extent of the T1 threshold and a higher than average rate of accumulation of AβPP-derived *i*Aβ.

## 15. Molecular Mechanisms Capable of Enacting the AβPP-Independent Production of C99 

### 15.1. The Singularity of the AUG Codon Encoding Met671 of Human AβPP

The present section briefly describes four molecular mechanisms potentially underlying the operation of the AβPP-independent C99 generation pathway (for more detailed descriptions, see [1,4]). They have one common feature: in all four mechanisms, the translation of human AβPP mRNA initiates from a position deep within its coding region. In all four mechanisms, this position is uniform, namely the AUG codon encoding Met671 of AβPP. The central role of the AUG encoding Met671 of human AβPP stems from its singular location within AβPP mRNA. Human AβPP cDNA was cloned and sequenced by three laboratories in 1987 [117,118,119]. Soon afterwards, two researchers, Breimer and Denny, discerned that the ATG encoding Met671 precedes immediately and in-frame the portion of the gene encoding the C99 fragment of AβPP [120]. The singularity of this position within the human AβPP gene (and AβPP mRNA) was indicated by the following two observations. One was that the AUG encoding Met671 of human AβPP is embedded within the optimal translation initiation nucleotide context, known as the Kozak consensus sequence [120]. Another observation was that of the twenty methionine-encoding codons within human AβPP mRNA, the AUG codon encoding Met671 is the only one that is situated within the optimal translation initiation context. Tellingly, not even the AUG codon encoding the translation-initiating Met of human AβPP (i.e., initiating its conventional translation) is situated within the optimal translation initiation context [120]. On the basis of the above observations, Breimer and Denny posited that such localization of the AUG codon encoding Met671 of human AβPP is not random, but is rather a reflection of its second (in addition to encoding Met671) physiological function, namely the initiation of translation of AβPP mRNA resulting in the AβPP-independent production of C99 and, potentially, of Aβ in Alzheimer’s disease-affected neurons [120].

### 15.2. Unconventional Translation of the Intact Human AβPP mRNA Initiating with Met671: A Viable Option Despite Being Ruled Out

More specifically, Breimer and Denny suggested that in Alzheimer’s disease, C99 and, subsequently, Aβ are produced via partial translation of the intact human AβPP mRNA initiating within its coding region at the AUG codon normally encoding Met671 (i.e., via unconventional internal initiation of translation of the intact AβPP mRNA). This proposition was eventually tested, albeit inconclusively, as described below, by two laboratories [121,122]. In both attempts, the rationale was identical. If translation of AβPP does indeed initiates within its coding region utilizing the AUG encoding Met671 as the initiation codon, alteration within the AβPP gene (and AβPP mRNA) within its coding region but upstream from the AUG encoding 671 would prevent the production of AβPP but would not interfere with the AβPP-independent generation of C99 and, subsequently, Aβ. On the other hand, if such alteration were to stop the production of Aβ, this would be indicative of the lack of the initiation of translation at the AUG codon encoding Met671 [121,122]. One laboratory inserted or removed one or two nucleotides and thus caused a frame-shift upstream from the AUG encoding Met67 [121]. Another laboratory introduced a translational stop codon upstream from the AUG in question [122]. The validity of the concept was assessed by transfecting altered cDNAs, within appropriate constructs, into cultured cells and analyzing their transient expression. No production of Aβ from transfected constructs was detected in either study and both concluded that the concept of the internal initiation of translation of intact AβPP mRNA from the AUG in question is invalid and can be ruled out [121,122]. However, the validity of the concept remains to be proven or disproven. What is certainly invalid is the conclusion of the above studies [121,122]. Breimer and Denny proposed that the internal initiation of translation of AβPP mRNA starting at the AUG encoding Met 671 might occur in human neurons under the AD conditions [120]. Cells employed in the above studies [121,122] were neither human nor neurons, and the conditions were not those of AD. Therefore, unconventional translation of human AβPP mRNA initiating at the AUG encoding Met671 is still a potentially viable option that should be reassessed in an appropriate experimental model (see [7] for more details).

### 15.3. Conventional Translation of 5′-Truncated Human AβPP mRNA Encoding Only the C100 Fragment: Additional Viable Options

Translation of AβPP mRNA initiating from the AUG encoding Met671 can occur in two ways. One is via unconventional translation, i.e., via the internal initiation at the AUG codon in question while the AβPP mRNA molecules remain intact, as was proposed by Breimer and Denny [120]. The other possibility is to truncate the 5′ portion of AβPP mRNA in such a way that in its remaining 3′ portion, the AUG codon in question is the first, 5′-most functional translation initiation codon. In this case, the translation of 5′-truncated AβPP mRNA would occur conventionally, with the AUG in question encoding the translation-initiating Met. Conceptually, therefore, all mechanisms capable of generating 5′-truncated AβPP mRNA where the AUG in question is the first translation initiation codon can be grouped into one category.

Such a category consists, conceivably, of three mechanisms. One is the internal initiation of transcription within the coding region of the human AβPP gene upstream from and in the vicinity of the AUG encoding Met671 so that this codon is the first in-frame AUG capable of serving as the translation initiation codon. This mechanism would require the induced production, presumably following the elicitation of the neuronal integrated stress response, of a transcription factor competent of guiding the internal initiation of transcription at the appropriate position within the AβPP gene. The second potential mechanism is the site-specific cleavage of the intact AβPP mRNA at the appropriate position upstream from the AUG encoding Met 671. The requirements of this mechanism are the same as in the previous scenario: the AUG in question should be the first in-frame translation initiation codon. In fact, 5′-truncated AβPP mRNA products of these two mechanisms would be, with one difference, conceptually identical. The difference is that the internal initiation of transcription would produce the capped 5′-truncated AβPP mRNA, whereas the site-specific cleavage would result in the uncapped 5′-truncated AβPP mRNA. The occurrence of such a mechanism would necessitate the, presumably, neuronal ISR-induced production of a specialized nuclease. The third, apparently highly plausible neuronal ISR-induced mechanism is the RNA-dependent asymmetric amplification of human AβPP mRNA. This is the only mechanism strongly supported by the empirical data. In view of its potential considerable significance, this process is analyzed in some detail in Section 16, Section 17 and Section 18.

### 15.4. Assaying the AβPP-Independent Generation of C99 and Aβ: C100 and Met-Aβ Generated Independently of AβPP Are Distinguishable from C99 and Aβ Produced by AβPP Proteolysis

The simplest way to assay the operation of a certain pathway is to detect its product. When Breimer and Denny proposed the occurrence of the AD-specific initiation of translation from the AUG codon encoding Met671 of human AβPP [120], such detection was considered impossible because, in a view held at that time, the products of the internal initiation of translation (presumably C99 and, subsequently, Aβ) would be indistinguishable from the corresponding products of the AβPP proteolytic pathway. In the proteolysis of AβPP, C99 is produced by the cleavage between Met671 and Asp672, Consequently, Asp is the N-terminal residue in both C99 and Ab. If, on the other hand, translation initiates from the AUG 671, the first N-terminal residue would be Met. At the time Breimer and Denny made their proposal, it was understood that this Met, encoded by the AUG in question, would be removed co-translationally by N-terminal methionine aminopeptidase (MAP) and that the resulting primary translation product would be C99 and, subsequently, Aβ, containing Asp at the N-terminus and indeed indistinguishable from C99 and Aβ produced in the AβPP proteolytic pathway. 

Subsequent investigations of the processing of the N-terminal methionine, however, established that the above view is incorrect, or, more precisely, not always correct [123,124,125,126,127,128] for the following reason. Prior to the occurrence of MAP-mediated cleavage, the translation-initiating Met has first to be accommodated in the enzyme’s active site together with the next amino acid residue. This requires that the geometry and the combined dimension of both residues are compatible with the geometry and dimensions of the MAP’s active site. Investigations showed that the N-terminal Met can be accommodated in the active site of MAP in combination with only the seven smallest amino acid residues, namely Gly, Ser, Cys, Thr, Pro, and Val [123,124,125,126,127,128]. In any other combination, MAP is incapable of removing the N-terminal Met and it is retained in the primary translation product [123,124,125,126,127,128].

As was mentioned above, the amino acid residue that follows Met 671 of AβPP is aspartate. Therefore, if translation were initiated with Met671 (irrespective of the molecular mechanism that enables such translation), the Met/Asp combination would not be accommodated within the MAP’s active site, and Met would not be cleaved-off co-translationally but would be retained in the primary translation product. This primary translation product would be, accordingly, not C99 but rather C100 (N-terminal Met-C99) and its cleavage by γ-secretase (provided it occurs; discussed further below) would produce not Aβ but rather Met-Aβ. However, neither C100 nor Met-Aβ would be the final product, rather only the intermediates. This is because the N-terminal Met, untouched by MAP, would be eventually removed by one of many aminopeptidases with broad specificity [128], thus converting C100 and Met-Aβ into C99 and Aβ, respectively. Significantly, such a conversion can happen only post-translationally, and, therefore, pools of C100 and Met-Aβ should occur in AD-afflicted neurons or in the appropriate model system [7]. These pools would be absent in postmortem tissue because in dying cells, the termination of protein synthesis precedes that of proteolysis, and in the absence of the influx of C100 and Met-Aβ, the conversion into C99 and Aβ would be complete.

The bottom line of the present subsection is that the AβPP-independent generation of Aβ can potentially be assayed by the identification of its products: the detection of either C100 or Met-Aβ in the appropriate experimental system could report on the occurrence of translation initiating at the AUG encoding Met671 of human AβPP irrespective of the molecular mechanism that enacts such translation. Note that the past, the present, and a few following sections assume that C100/C99 generated independently of AβPP are processed into Met-Aβ/Aβ. However, as discussed in Section 34, there is a distinct possibility that in AD, under the neuronal ISR conditions, the production of γ-secretase and the AβPP-independent production of Aβ are suppressed, and the neuronal ISR is propagated and the disease is driven by C100/C99 generated independently of AβPP. In such a case, the detection of C100 would report on its generation independently of AβPP.

## 16. Mammalian RNA-Dependent Synthesis of mRNA: An Analog of Massive Gene Amplification

The present and several following sections consider a possibility that 5′-truncated human AβPP mRNA with the AUG encoding Met671 as its first translation initiation codon is generated, and, consequently, the AβPP-independent production of *i*Aβ is enacted by the RNA-dependent asymmetric amplification of human AβPP mRNA. However, prior to getting to the subject of our discussion, we should briefly describe the general tenets of mammalian RNA-dependent mRNA synthesis. Mammalian RNA-dependent mRNA synthesis occurs, apparently physiologically, in situations requiring augmented production of specific polypeptides [129,130,131,132,133,134,135,136,137,138,139]. Known examples of this process include the erythroid differentiation-associated massive production of both α and β globins [129,130,131] as well as the immense generation of matrix proteins associated with their extracellular deposition [132]. Mammalian RNA-dependent mRNA synthesis constitutes a process of massive amplification of genome-encoded protein coding information, where every conventional (i.e., gene-transcribed) mRNA is utilized repeatedly as a template in the generation of new mRNA molecules. Abundant mRNA species can occur in the hundreds, and even thousands of gene-transcribed molecules per cell. Since each of these molecules can serve as a template for the production of new mRNA, mammalian RNA-dependent mRNA synthesis is analogous to a massive, multiple orders of magnitude gene amplification.

**Figure 5:**
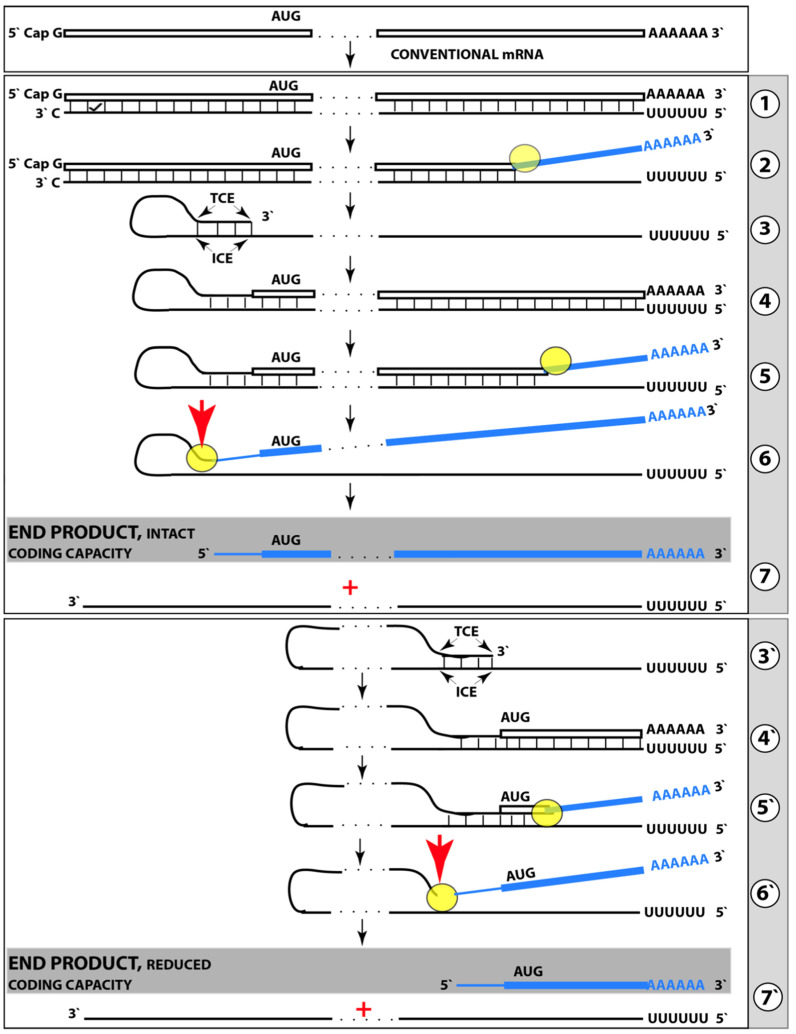
**Principal stages of the chimeric pathway of mammalian RNA-dependent mRNA amplification**. *RdRp*: RNA-dependent RNA polymerase. *Single line*: Antisense RNA. *Boxed line*: Sense RNA. *AUG*: Translation initiation codon. *TCE*: 3′ Terminal Complementary Element of the antisense RNA. *ICE*: Internal Complementary Element of the antisense RNA. *Yellow circle*: A complex containing helicase, nucleotide-modifying, and RNA-cleaving activities. *Blue lines*: Separated single-stranded RNA molecules or portions thereof. *Red arrows*: The site of cleavage of the chimeric RNA intermediate. *Top Panel*: A progenitor of the RNA-dependent mammalian mRNA amplification—conventionally genome-transcribed mRNA molecule. *Middle Panel*: Principal stages of the chimeric pathway of the mRNA amplification process. Stage **1**: Antisense RNA is transcribed by RdRp from the progenitor mRNA. Stage **2**: Sense and antisense RNA molecules are separated by the helicase activity. Helicase mounts the 3′ poly(A) segment and moves along the sense orientation molecule modifying, on average, every fifth nucleotide. Stage **3**: TCE/ICE interaction-guided folding of the antisense RNA into self-priming configuration. Stage **4**: RdRp extends the 3′ end of self-primed antisense RNA; the result is a hairpin-like chimeric molecule containing sense and antisense RNA components and referred to as the chimeric RNA intermediate. Stage **5**: The double-stranded portion of the chimeric RNA intermediate is separated by helicase; nucleotide modifications introduced during the separation prevent re-annealing of the RNA strands. Stage **6**: The helicase complex cleaves the chimeric RNA intermediate upon reaching its single-stranded portion. Stage **7**: Chimeric RNA and antisense RNA end products. The antisense RNA is 3′-truncated, and its cleaved-off segment becomes the 5′ terminus of the chimeric RNA end product. Note: In this scenario, the ICE is situated within a portion of the antisense RNA complementary to a segment of the 5′UTR of the progenitor mRNA. Therefore, the chimeric RNA end product retains the entire coding region of the progenitor. *Bottom Panel*: The ICE is located within a portion of the antisense RNA complementary to a segment of the coding region of the progenitor mRNA. The chimeric RNA end product is 5′-truncated within the coding region of the progenitor. The potential outcome of its translation is defined by the position of the first functional translation-initiating codon. If it is located within the retained portion of the coding region and is in frame, the outcome would be the CTF of the progenitor mRNA-encoded protein. Stages **3′**–**7′** of the bottom panel correspond to stages **3**–**7** of the middle panel.

Mammalian RNA-dependent mRNA amplification can occur in two steps. The first step designated “the chimeric amplification pathway” and described below is linear [130,131]. One of its end products, if polyadenylated at the 3′ terminus, can repeatedly serve as the progenitor in the second step of the mRNA amplification process; this step is exponential and constitutes a polymerase chain reaction [131,137]. The latter, however, is not relevant to the subject of the present study, and only the linear chimeric amplification pathway is discussed below. Stages of the chimeric amplification pathway are illustrated in Figure 5, where the top panel shows the initial conventionally generated mRNA molecule, referred to henceforth as “progenitor mRNA”, and the middle panel depicts main stages of RNA-dependent mRNA synthesis. Stage 1 consists of transcription of the progenitor mRNA by RNA-dependent RNA polymerase (RdRp). It starts within the 3′ terminal poly(A) portion of the progenitor molecule and concludes with transcription of the capG. The newly generated antisense RNA is in a double-stranded conformation with the progenitor. Stage 2, therefore, consists of the separation of strands by a helicase. Separation initiates at the 3′ poly(A) of the template; when it is completed, the template, i.e., progenitor mRNA, can be reused. 

The following Stage 3 is dependent upon the ability of the antisense RNA to fold into a self-priming configuration. Such folding requires the presence within antisense RNA of two segments that are sufficiently complementary to form a stable double-stranded structure. One, referred to as the 3′ Terminal Complementary Element (TCE), has to be, by definition, 3′-terminal. The second requisite segment, referred to as the Internal Complementary Element (ICE), can be positioned at any location within the antisense RNA. Importantly, the presence of the TCE and ICE within the antisense RNA could be insufficient, on its own, for the formation of a self-priming structure; the additional requirement for this is the topological compatibility, i.e., the mutual accessibility of the two elements within the folded antisense RNA.

Upon folding of the antisense RNA into self-priming configuration, its 3′ terminus is extended by RdRp into the sense-orientation RNA terminating with the 3′ poly(A); this constitutes Stage 4 of the amplification process. The product of this stage consists of continuous antisense (5′ portion) and sense (3′ portion) RNA in a hairpin configuration and is referred to, accordingly, as the chimeric RNA intermediate. The position where the RNA orientation changes from antisense to sense is referred to as the chimeric junction; it is, in fact, the position of the commencement of the extension of the 3′ end of the self-primed antisense RNA. The occurrence of these features (which also appear in one of the end products, see below) is the reason why this mRNA amplification pathway has been named the chimeric pathway. In Stage 5 of the chimeric mRNA amplification pathway, the double-stranded part of the chimeric RNA intermediate is separated by a helicase invoked earlier (in Stage 2). Helicase mounts the 3′ poly(A) segment and proceeds in the 5′ directions. Upon reaching the single stranded part of the hairpin structure (now separated), the helicase complex cleaves the chimeric RNA intermediate; this may happen at a TCE/ICE mismatch or, if there are no mismatches, at the 3′ end of the hairpin’s loop, as shown in Figure 5. If the former takes place and the folded antisense RNA structure remains in a stable self-priming configuration, it can be extended again and the chimeric junction, i.e., the site of the initiation of extension, would shift due to the shortening of the TCE, a process referred to as the chimeric junction shift (described in more detail in [7]). The separation of strands of the chimeric RNA intermediate and its cleavage constitute Stage 6 of the chimeric mRNA amplification pathway. 

The portion of Figure 5 marked “Stage 7” depicts two RNA molecules that constitute the end products of the chimeric mRNA amplification pathway. One end product is what remains of the antisense RNA. It is truncated in its 3′ portion. It has lost at least a part of or possibly its entire TCE element and can no longer fold into the self-priming configuration. In the chimeric mRNA amplification pathway, it has no more uses and thus is a quintessential end product (albeit it can potentially be extended to the full size on the progenitor mRNA template and enter another round of amplification, but for a number of reasons this is unlikely). On the other hand, if it were polyadenylated at the 3′ terminus in conjunction with the cleavage, it would possess both 3′-terminal poly(A) and 5′-terminal poly(U) (acquired in Stage 1 of the chimeric mRNA amplification pathway). In such a case, for obvious reasons, it would constitute the initial template, a progenitor, in the polymerase chain reaction type of mRNA amplification (discussed in detail in [7]).

Another end product of the chimeric mRNA amplification pathway is chimeric mRNA. It differs from the progenitor mRNA in two ways. One, it is chimeric. It contains as its 5′-terminal portion a segment of the antisense RNA, in fact the same segment of the antisense portion of the chimeric RNA intermediate that was cleaved off at Stage 6; depending on the position of cleavage, this segment consists of a part or the entire TCF element. Another difference is that the 5′ portion of the chimeric end product RNA is truncated. The nature and the consequences of such truncation are discussed in the following section and elsewhere. The chimeric end product RNA cannot be utilized as a progenitor in another round of amplification because the corresponding antisense RNA would be lacking the TCE and ICE components. It may, however, be translated and give rise to a polypeptide (further discussed below). It would be cap-less but, since it is apparently generated under the ISR conditions [131,137], the lack of the 5′ cap would not be an impediment for its translation. 

## 17. Asymmetric mRNA Amplification Produces a C-Terminal Fragment (CTF) of the Gene-Encoded Polypeptide

The protein-coding potential of the chimeric end product RNA is determined by two factors. One is the position of the cleavage of the chimeric RNA intermediate. This position, in turn, is determined by the location of the ICE component of the antisense RNA. The ICE component determines the structure of the self-primed antisense RNA as well as of the self-primed extension-generated chimeric RNA intermediate by defining, i.e., “fixing” the position of the TCE component. The cleavage occurs either within (at a TCE/ICE mismatch) or at the 5′ end of the TCE component but the position of the latter is dictated by the location of its ICE counterpart. The TCE component of the antisense RNA is defined as “terminal” (C-terminal element) because this is its requisite location; it would be nonfunctional if located elsewhere. The location of the ICE component of the antisense RNA is variable; potentially, it can be located anywhere within the antisense RNA molecule. The middle panel of Figure 5 depicts a scenario where the ICE component of the antisense RNA is positioned in its segment corresponding to (i.e., transcribed from by RdRp) the 5′ untranslated region (5′UTR) of the progenitor mRNA molecule. In this scenario, the cleavage occurs upstream from the AUG translation initiation codon of the progenitor, and the chimeric RNA end product contains the complete protein coding information of the progenitor mRNA; its translation would yield a polypeptide indistinguishable from and identical to the polypeptide translated from the progenitor mRNA. Potentially, however, the ICE component of the antisense RNA can be positioned within its segment corresponding to the coding region and even to the 3′ untranslated region (3′UTR) of the progenitor mRNA (discussed in detail in [131,137]).

The second factor that determines the coding potential of the chimeric RNA end product and the translational outcome of the chimeric mRNA amplification pathway is the position of the first functional translation initiation codon within the chimeric end product RNA (in the category of cases discussed in the preceding section the ICE component of the antisense RNA is positioned within its segment corresponding to the 5′UTR of the progenitor mRNA; in such cases the translation-initiating AUG of the progenitor RNA is retained and utilized in the chimeric end product RNA, reviewed in [130,131]). As discussed elsewhere [131,137], translational outcomes of the chimeric mRNA amplification pathway can vary from a polypeptide unrelated to the one encoded in the progenitor mRNA to a chimeric polypeptide composed of segments encoded in two distinct genomic locations [137].

The present section describes a scenario depicted in the bottom panel of Figure 5. In this scenario, the ICE component of the antisense RNA is localized within its segment corresponding to the coding region of the progenitor mRNA molecule. Stages 3′ through 7′ of this panel are the equivalents of Stages 3 to 7 of the middle panel. The only distinction is that in Stages 3 to 7, the ICE component of the antisense RNA is located within its segment corresponding to the 5′UTR of the progenitor mRNA, whereas in Stages 3′ to 7′ the location of the ICE component is within the antisense segment corresponding to the coding region of the progenitor mRNA molecule (Stage 3′). Since the position of the TCE component is defined by that of the ICE, the extension of the self-primed antisense RNA would yield only the 3′ portion of the coding region of the progenitor mRNA followed by its 3′UTR and 3′-terminal poly(A) (Stage 4′). Following separation of sense and antisense strands of the chimeric RNA intermediate (Stage 5′), the cleavage would occur either within or at the 5′ end of the TCE component (Stage 6′). Thus, the resulting chimeric RNA end product would be truncated within the coding region of the progenitor mRNA (Stage 7′). The protein coding potential of this chimeric end product RNA would be established and the translational outcome of the chimeric mRNA amplification process would be decided by the position of the first functional translation initiation codon. Provided it is situated within the remaining portion of the coding region and is within its coding frame, translation of the chimeric end product RNA would produce a CTF of the protein encoded by the progenitor mRNA. This variant of the RNA-dependent synthesis of mRNA has been designated the “asymmetric chimeric mRNA amplification pathway” [130,131]. It is “asymmetric” because only the 3′ portion of the progenitor mRNA is amplified and only a C-terminal fragment of the progenitor mRNA-encoded polypeptide is generated upon translation of the chimeric end product RNA.

## 18. Human AβPP mRNA Is an Eligible Template of Asymmetric Amplification—The Resulting mRNA Encodes the C100 Fragment of AβPP

The asymmetric chimeric mRNA amplification pathway described in the preceding section provides potential mechanistic means for generating the mRNA defined in Section 15.3 above, namely the 5′-truncated human AβPP mRNA, where the first, 5′-most translation initiation codon is the AUG encoding Met671 of AβPP. However, to determine the applicability of the asymmetric mRNA amplification to the human AβPP mRNA, two principal questions need to be answered. The first is whether human AβPP mRNA is an eligible template for the RNA-dependent mRNA amplification process. If, and only if, the answer is affirmative, the second question is whether the RNA-dependent amplification of human AβPP mRNA can produce the chimeric RNA end product where the AUG encoding Met671 is the first, 5′-most translation codon. Considering that the 5′ terminus of AβPP mRNA (potentially encoding the TCE element of its antisense counterpart) is separated by over 2000 nucleotides from the AUG encoding Met671, answering the above questions appears demanding. Fortunately, however, the answer to whether AβPP mRNA or, actually, any eukaryotic mRNA encodes, when transcribed into the antisense RNA, the TCE and ICE elements and whether these elements are mutually accessible can be obtained experimentally. Moreover, if successful, the experiment would also define the identities of the TCE and ICE components and determine the precise position of the ICE.

Such an experiment imitates the RNA-dependent mRNA amplification process but substitutes RdRp with RdDp (RNA-dependent DNA polymerase; reverse transcriptase). An mRNA of interest is reverse-transcribed starting at its 3′-terminal poly(A). Following the completion of the antisense strand (cDNA), the mRNA template is degraded by RNase H, which is typically present in genetically unmodified preparations of RdDp. At this point, the situation is comparable to that depicted in the initial stages of Figure 5. As in Figure 5, the antisense strand has been generated and removed from its template. Moreover, as in Figure 5, the enzyme (RdDp) capable of extending the 3′ terminus of the antisense strand, provided it forms a self-priming structure, is present in the mixture. Therefore, if the antisense strand contains the TCE and ICE components and if these components were mutually accessible in the folded configuration, the antisense strand would form a self-priming structure, and its 3′ end would be extended by RdDp into the sense-orientation strand. The occurrence of the extension would be indicated by a simple size assessment of the resulting cDNA, and its nucleotide sequence would determine the nature of the TCE and ICE components and define their position.

Such an experiment was, in fact, carried out, albeit unintentionally, with human AβPP mRNA. As mentioned above, in 1987, several laboratories succeeded in cloning and sequencing human AβPP cDNA [117,118,119]. Shortly afterwards, another laboratory cloned and sequenced human AβPP cDNA that was significantly larger than previously determined; it was identical to previously sequenced human AβPP cDNAs but contained an additional segment in its 3′ portion [140]. The authors of this study initially suggested that the detected 3′-extended AβPP cDNA was produced from the appropriately 5′-extended AβPP mRNA, which was presumably transcribed from a site (transcription start site, TSS) upstream from that determined in the previous studies [117,118,119]. At the time of publication of [140], the genomic sequence upstream of the human AβPP gene was not yet known, but soon afterwards it was determined [141] and it became clear that the 3′ extension seen in human AβPP cDNA does not match the genomic nucleotide sequence upstream from the human AβPP gene. Therefore, the authors concluded that their observation was an artifact and corrected their paper accordingly [140].

However, the subsequent analysis of the 3′ extension of human AβPP cDNA seen in [140] determined that it is actually a fragment of human AβPP DNA (gene) in sense-orientation [142,143,144]. The simplest and, probably, the only explanation of its origin is that the extension occurred precisely as described in the present section above, namely through the formation by cDNA, upon its completion and separation from its mRNA template, of a self-priming structure. The reason why other groups did not see the extension is because they used, apparently, more advanced genome-manipulated cloned preparations of RdDp where the RNase H activity has been removed; consequently, AβPP cDNA, in complex with its mRNA template, was unable to form a self-priming structure. The analysis also identified the position of initiation of the extension of self-primed human AβPP cDNA and defined both the TCE and ICE components of the antisense strand (cDNA), which are indeed separated by over 2000 nucleotides. 

These findings answer the questions posited at the beginning of the present section. They demonstrate that: (1) human AβPP mRNA is the eligible template for the chimeric RNA-dependent mRNA amplification process; (2) amplification of human AβPP mRNA in the chimeric mRNA amplification pathway would occur asymmetrically; (3) in the asymmetrically amplified 5′-truncated human AβPP mRNA, the first translation initiation codon is the AUG encoding Met 671. When translated, it would produce the C100 fragment of AβPP.

**Figure 6:**
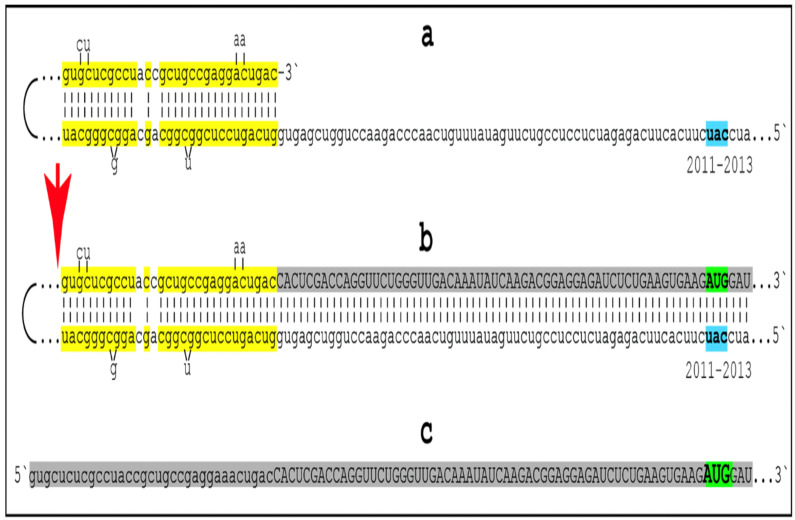
**Human AβPP mRNA is an eligible template of asymmetric amplification; the resulting mRNA encodes the C100 fragment of AβPP**. *Lowercase letters*: nucleotide sequences of the portions of the human antisense AβPP RNA capable of forming the self-priming structure. *Uppercase letters*: nucleotide sequence of a segment of human AβPP mRNA generated by the extension of self-primed human antisense AβPP RNA. *Highlighted in yellow*: the TCE (3′-terminal segment) and the ICE (internal segment) elements of the antisense RNA. *2011–2013*: Positions of nucleotides counted from the 3′ terminus of the antisense RNA and forming the “UAC” (*highlighted in blue*) complementary to the AUG codon (*highlighted in green*) encoding Met671 of human AβPP. (**a**–**c**) parallel stages 3′ to 7′ of Figure 5. (**a**) TCE/ICE interaction-guided folding of human antisense AβPP RNA into self-priming configuration. (**b**) RdRp-mediated extension of the 3′ terminus of self-primed human antisense AβPP RNA; the resulting segment of human AβPP mRNA is *highlighted in gray*. When, upon separating the RNA strands, helicase reaches the single-stranded portion of the chimeric RNA intermediate, it cleaves either at its 3′ end (*red arrow*) or at a mismatch within the TCE/ICE structure. (**c**) The chimeric RNA end product (*highlighted in gray*) consists of AβPP mRNA 5′-truncated within its coding region and containing a portion of the antisense RNA appended to its 5′ end. Its first, 5′-most translation initiation codon is the AUG encoding methionine 671 of human AβPP.

The anticipated folding of human antisense AβPP RNA into a self-priming configuration and its subsequent extension are depicted in Figure 6. Panels (a) to (c) of Figure 6 correspond to panels 3′ to 7′ of Figure 5. The TCE and ICE components of the antisense RNA, which are separated by about two thousand nucleotides but are nevertheless topologically compatible, are highlighted in yellow. The folded self-priming configuration of human antisense AβPP RNA is depicted diagrammatically in panel (a) of Figure 6. Panel (b) shows the extension of self-primed antisense AβPP RNA into the sense-orientation RNA; it is completed when the 3′-terminal poly(A) segment is formed. Complementary sense and antisense RNA strands of the chimeric RNA intermediate are then separated and the cleavage occurs either at the 5′ end of the TCE component, as shown in panel (b) of Figure 6 (red arrow), or at a mismatch within the TCE/ICE complex. Panel (c) of Figure 6 shows the chimeric RNA end product of the asymmetric amplification of human AβPP mRNA. It is a chimeric molecule. At the 5′ end, it contains a 3′-terminal segment of the antisense RNA (the TCE component or a segment thereof, depending on the site of cleavage of the chimeric RNA intermediate). Its sense-orientation portion consists of a 5′-truncated coding region followed by the 3′UTR and poly(A) of human AβPP mRNA. Importantly. its first functional translation initiation codon is the AUG (highlighted in green) encoding Met 671. Its translation would produce C100/C99 independently of AβPP.

## 19. Modulation of Transcription Start Sites of Human AβPP Gene as a Potential Therapeutic Strategy for Alzheimer’s Disease

The AβPP gene belongs to a large category of genes lacking the “TATA” promoter element. When it is present, the TATA element (known as the “TATA box”) defines the position of TSS; usually, transcription starts 30 nucleotides downstream from the TATA element. Genes lacking the TATA element are characterized by multiple TSSs. In this respect, the AβPP gene is a typical example. It contains five transcription start sites [141] situated in close vicinity to each other. Their nucleotide positions, counted in the 5′ direction from the AUG codon that initiates conventional translation, are (-)150, (-)149, (-)146, (-)144, and (-)143. These positions define 3′ ends of corresponding antisense AβPP RNA. Of those, only one, namely human antisense AβPP RNA terminating at the 3′ position (-)149, can fold into a self-priming configuration, as shown in Figure 6. All other human antisense AβPP RNA species contain 3′-terminal overhang in their folded conformations, and even if the extension can occur, its efficiency and, consequently, the efficiency of the AβPP-independent C100/C99 generation pathway would be greatly reduced (discussed in detail in Section 20 of [7]). This factor introduces heterogeneity into the phenomenology of AD. More specifically, the frequency of the utilization of TSS (-)149 potentially defines the rate of the AβPP-independent production of C100/C99 and, consequently, the rate of the progression of the disease. It also suggests potential AD therapy via regulation of the utilization of TSSs of the human AβPP gene. If the usage of TSS at the position (-)149 was excluded or minimized, the severity of AD could be greatly reduced. Moreover, if transcription of human AβPP mRNA was initiated, by therapeutic means, in such position that its antisense complement would lack the TCE element, the operation of the AβPP-independent C100/C99 generation pathway would be precluded and the occurrence of AD would be prevented.

## 20. Superimposition of Two Singularities: AD Is Human-Specific or at Least Species-Specific; Mouse AβPP mRNA and That of Other Species Are Ineligible for Amplification 

For the reasons propounded above, of the four mechanisms potentially enacting the AβPP-independent production of C100/C99, the asymmetric RNA-dependent amplification of human AβPP mRNA is by far the most plausible. Please reflect on the following. Breimer and Denny considered the propituous positioning of the AUG encoding Met671 of AβPP (see above) a singularity that rationalizes the conclusion that this codon potentially initiates translation of the downstream portion of human AβPP mRNA [120]. By the same logic, the singularity of the presence within human antisense AβPP RNA of the TCE and ICE elements that are mutually accessible, despite being separated by over 2000 nucleotides [142,143,144], is of a degree at least equal if not much greater than that described in [120]; this, arguably, also rationalizes a physiological function, namely the occurrence of the RNA-dependent asymmetric amplification of human AβPP mRNA. Most remarkably, these two singularities converge in a superimposed, complementary, and synergistic manner in one common manifestation: the chimeric RNA end product, structured just so that its first functional translation initiation codon is the AUG normally encoding Met671 of AβPP, is generated via the singular TCE/ICE-guided folding of human antisense AβPP RNA and translated into the C100 fragment of AβPP due to the singularity of the positioning of the AUG codon in question. If one singularity is sufficient to rationalize a function, the convergence and superimposition of two singularities makes a physiologically occurring function enabled by these singularities a near certainty. 

**Figure 7:**
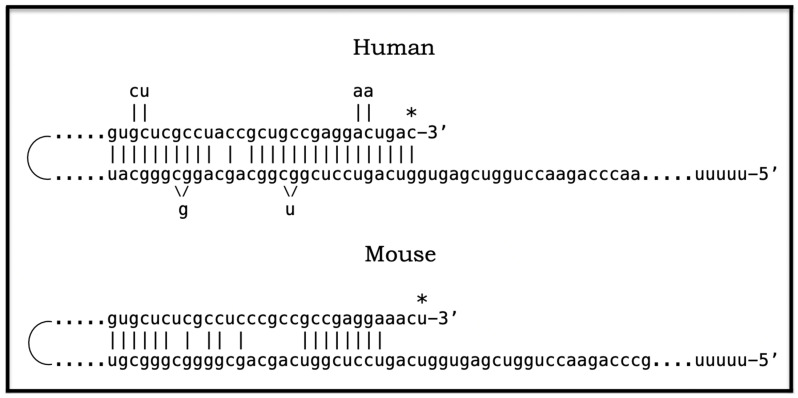
**Mouse AβPP mRNA is ineligible for RNA-dependent mRNA amplification**. (**Top**) Human antisense AβPP RNA folded into self-priming structure. (**Bottom**) The relationship between the portions of the mouse antisense AβPP RNA in positions analogous to those of the human TCE and ICE elements. *Asterisks* denote the nucleotide position corresponding to the transcription start site of either human or mouse AβPP mRNA; in both cases, it is the nucleotide (-)149 upstream from the AUG translation initiation codon. The relationship between segments on mouse antisense AβPP RNA corresponding to the TCE and ICE elements of its human counterpart is, in its strength, no better than random. Moreover, the occurrence of the 3′ overhang in the “folded” mouse antisense AβPP RNA is incompatible with the priming function.

Not only does the asymmetric RNA-dependent amplification of human AβPP mRNA explain the AβPP-independent production of C100/C99 in Alzheimer’s disease, it also explicates why neither mice nor even long-living mammalian species, such as elephants, develop AD, and why the asymmetric amplification of human AβPP mRNA expressed exogenously from human transgenes is inoperative in animal transgenic models. Figure 7 shows a comparison between the interaction of the TCE and ICE components of human antisense AβPP RNA and that of the segments of mouse antisense AβPP RNA corresponding to the human TCE and ICE elements. In human antisense AβPP RNA, the TCE and ICE elements exhibit a substantial degree of complementarity, certainly sufficient for forming a self-priming structure. The formation of such a structure is enabled, as discussed above, by the apparent topographic compatibility, i.e., mutual accessibility of the TCE and ICE within the folded conformation of the RNA molecule.

The folding pattern of the mouse antisense AβPP RNA could be entirely different from that of its human counterpart, and even if the TCE and ICE components were present, they would not necessarily be mutually accessible within the folded conformation of the molecule. This is because, despite human and mouse AβPP being almost identical, the nucleotide sequences of the corresponding mRNAs (and of their antisense counterparts) differ significantly. But this is beside the point because the TCE and ICE components are apparently not present in the mouse antisense AβPP RNA. As illustrated in Figure 7, the degree of complementarity of the mouse antisense AβPP RNA segments corresponding to the TCE and ICE components of the human antisense AβPP RNA is no better than random, with a significant 3′-overhang. Moreover, the blast analysis of the forty 3′-terminal nucleotides segment of the mouse antisense AβPP RNA with the entire molecule shows that the 3′-terminal segment has no sufficient complementarity that would enable (provided the mutual accessibility condition is satisfied) the formation of self-priming structure. Thus, mouse AβPP mRNA is not a legitimate template for the RNA-dependent mRNA amplification. Apparently, and for the same reason, this conclusion applies to most, if not all, mammalian AβPP mRNAs, even those of the long-living species, such as elephants. Alzheimer’s disease can be defined, therefore, as a human-specific, or at least a species-specific condition.

## 21. Transgenic Animal Models Possess RNA-Dependent mRNA Amplification Machinery but mRNA Transcribed from Human AβPP Transgenes Is Ineligible for Amplification

The preceding section explains why mouse AβPP mRNA, and probably, AβPP mRNA in many, if not most or even all (except human) mammalian species is ineligible for RNA-dependent mRNA amplification. The reason is the absence of the TCE and ICE elements of their antisense RNA complements. But then the question is why is there evidently no RNA-dependent mRNA amplification of human AβPP mRNA expressed in mouse model systems exogenously from human transgenes? There is more than a single possible explanation for this. One is that mice do not possess the machinery required for RNA-dependent mRNA amplification. This, however, is patently not the case. It was demonstrated empirically that, upon the elicitation of the integrated stress response by, for example, altered heme levels or overexpressed protein species, mouse cells do amplify, in an RNA-dependent manner, globin and extracellular matrix protein-encoding mRNAs [137]. Then, maybe no integrated stress response is elicited in mouse neurons? Again, no, it is. It was, indeed, shown empirically that the exogenous overexpression of human AβPP triggers the ISR in mouse neurons (for further discussion, see Section 26 below). This leaves the only possibility: for some reason, AβPP mRNA expressed from human transgenes in mouse neurons is not an eligible template for the RNA-dependent mRNA amplification. And, in fact, this explanation is the correct one. In all vectors carrying human AβPP gene (cDNA), the 5′ terminus of the gene has been heavily modified for its insertion into the construct. Since a short 5′-terminal segment (about 35 nucleotides) of human AβPP mRNA encodes the TCE element of the corresponding antisense AβPP RNA, modifications of the 5′-terminal region of the gene inevitably interfere with—or, in fact eliminate—the TCE component of its antisense counterpart (it also potentially interferes with the folding of the resulting antisense AβPP RNA). Therefore, in this respect, the exogenous human transgenes-encoded AβPP mRNA is equipped no better than the endogenous mouse AβPP mRNA to support its RNA-dependent amplification.

## 22. The Influx of AD Pathology-Driving *i*Aβ Produced in the AβPP-Independent Pathway Is Orders of Magnitude Greater than That Derived from AβPP

To better understand the dynamics of Aβ production and *i*Aβ accumulation in transgenic animal models of AD, it is of importance to compare the efficiencies of the influx of AβPP-derived *i*Aβ with that of *i*Aβ produced independently of AβPP. As described above, the influx of AβPP-derived *i*Aβ is composed of two parts. One is the cellular uptake of a fraction of secreted extracellular Aβ and another is the Aβ product of a fraction of AβPP-derived C99 processed not on plasma membranes but rather on intraneuronal membranes within various organelles and consequently retained inside the neurons as *i*Aβ. In both cases, the fractions are rather small. For the purpose of comparison, we can safely estimate that no more than 10% of Aβ produced in the AβPP proteolytic pathway becomes *i*Aβ. In terms of the ACH2.0, on the other hand, Aβ generated in the AβPP-independent pathway is retained in its entirety intraneuronally. This constitutes an order of magnitude difference between the influx of *i*Aβ derived from AβPP and that of *i*Aβ generated independently of AβPP. On top of this, for one molecule of Aβ produced by AβPP proteolysis, a 770-residue-long precursor molecule has to be generated whereas, for one molecule of Aβ produced independently of AβPP, only a 100-amino-acids-long polypeptide precursor has to be synthesized. This adds roughly another order of magnitude difference in the rates of influx. When we take into account the plausibility that the AβPP-independent generation of *i*Aβ is enacted via the RNA-dependent asymmetric amplification of human AβPP mRNA, an equivalent of massive gene amplification, additional orders of magnitude are appended to the difference in the rates of influx of *i*Aβ derived from AβPP and *i*Aβ produced independently of AβPP. This high rate of influx of *i*Aβ is apparently requisite in attaining its levels capable of causing AD pathology and driving the disease; without the operational AβPP-independent *i*Aβ generation pathway, AD cannot occur physiologically. This is why mice and other non-human mammalian species, where this pathway is inoperative, do not develop Alzheimer’s disease.

## 23. Animal Models Expressing Numerous FAD Mutations-Containing Human AβPP Transgenes Should Develop AD but Do Not 

**Figure 8:**
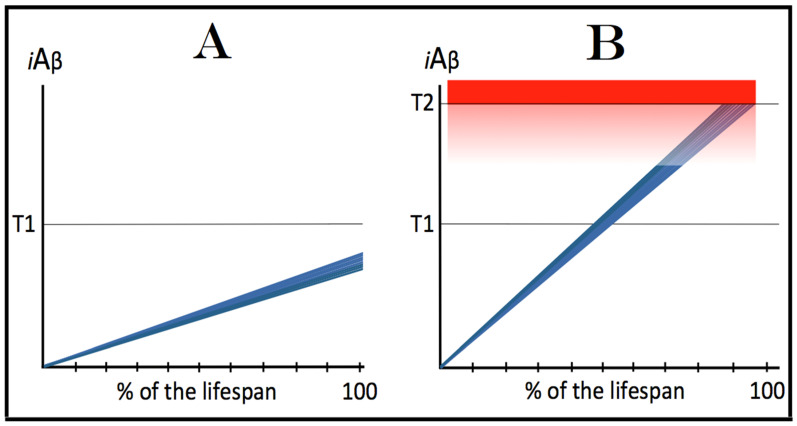
**Projected dynamics of accumulation of AβPP-derived *i*Aβ in transgenic mouse models overexpressing human AβPP. *i*Aβ**: intraneuronal Aβ. *Blue lines*: levels of *i*Aβ in individual neurons. ***T*1** *threshold*: Cellular concentration of *i*Aβ that triggers the elicitation of the neuronal integrated stress response. ***T*2** *threshold*: levels of *i*Aβ that trigger apoptosis or necroptosis of neuronal cells. *Gradient-pink box*: Range of concentrations of *i*Aβ that cause and propel AD pathology. *Red box*: Apoptotic zone; within this zone the neurons have either undergone apoptosis or are dead. (**A**) Accumulation of AβPP-derived *i*Aβ in a wild mouse. The rate of accumulation is exceedingly slow. AβPP-derived *i*Aβ neither reaches nor crosses the T1 threshold within the lifetime of the mouse. (**B**) Projected accumulation of exogenous human AβPP-derived *i*Aβ in transgenic mouse models. For reasons given in the main text, the rate of accumulation of exogenous AβPP-derived *i*Aβ should be on par with that of *i*Aβ produced independently of AβPP in AD patients. It should reach the AD pathology-causing range, cross the T2 threshold, and elicit full symptomatic spectrum of AD. This, apparently, does not happen.

If, however, the rate of influx of *i*Aβ defines the occurrence of AD or the lack thereof, the disease should develop in at least some transgenic mouse models. Indeed, consider the following. The models under discussion express human AβPP mRNA (albeit ineligible for the RNA-dependent mRNA amplification process) from multiple, in some cases over one hundred, transgenes. This creates already about two orders of magnitude difference in rates of influx of endogenously produced AβPP-derived *i*Aβ versus that of *i*Aβ derived exogenously via the expression of the transgenes. But this considerable difference is magnified even further. This is because the human AβPP transgenes contain various combinations of the FAD mutations and, moreover, in some cases, in addition to FAD mutation-containing AβPP transgenes, FAD-causing mutation-containing PSEN genes as well as ApoE4-encoding genes are also introduced into the same model. These mutations ensure the following. (1) Significant augmentation of the production of the Aβ42 isoform. This Aβ variant has greatly increased propensity to aggregate, causes more severe neuronal damage and lowers the neuronal ISR-eliciting T1 threshold [4]. (2) Significant increase in the rate of internalization of extracellular Aβ, i.e., of the rate of influx of AβPP-derived *i*Aβ via its intraneuronal uptake. This is because the Aβ42 variant is taken up by the cell at the rate twice that of other variants of Aβ and because ApoE4 is substantially more efficient in facilitating the internalization of Aβ than other ApoE isoforms. (3) Substantial increase in the fraction of γ cleavages occurring on intraneuronal membranes (rather than on plasma membranes) and yielding Aβ retained intraneuronally as *i*Aβ. All this appends additional orders of magnitude to the difference in the rates of influx of endogenously produced AβPP-derived *i*Aβ versus that generated exogenously from human AβPP transgenes. The difference in the rate of accumulation of *i*Aβ in wild mice and in transgenic models is illustrated in Figure 8. The bottom line is that, in these models, the rate of accumulation of human *i*Aβ is apparently on par with and probably even exceeds that of *i*Aβ in AD. Therefore, these models should develop the disease. They do not. 

## 24. Why Transgenic Mouse Models Do Not Develop AD: Both Endogenous and Exogenous Production of Aβ in the AβPP Proteolytic Pathway Is Either Discontinued or Severely Suppressed Under the Neuronal ISR

Within the framework of the considerations presented above, the most plausible, possibly the only answer to the conundrum discussed in the preceding section is that under the conditions of the neuronal ISR the production of Aβ in the AβPP proteolytic pathway is either discontinued or severely suppressed. This suppression is, in fact, self-inflicted. Massive overproduction in mouse transgenic models of Aβ from multiple human AβPP transgenes harboring various combinations of FAD mutations in the presence of mutated exogenously expressed PSEN and ApoE4 leads, for the reasons elaborated above, to the rapid accumulation of AβPP-derived *i*Aβ. When its levels reach and cross the T1 threshold, the PKR and/or HRI kinases are activated, eIF2α is phosphorylated at its Ser51 residue, and the neuronal integrated stress response is elicited. Under the ISR conditions, the global cellular translation is severely suppressed, and there is no good reason to presume that the production of Aβ in the AβPP proteolytic pathway is exempted from this general phenomenon. Moreover, the suppression of the generation of AβPP-derived Aβ can, potentially, be enacted in several ways: the suppression of translation of AβPP, the suppression of the production of BACE enzymes, the inhibition of translation of the components of γ-secretase, or all of the above (further discussed below). The suppression of the production of AβPP-derived Aβ (and, consequently, *i*Aβ) is self-inflicted, a “self-suppression”, because it is triggered by AβPP-derived *i*Aβ accumulated to sufficient, ISR-eliciting levels. This suppression explains why *i*Aβ cannot reach AD pathology-causing levels and, consequently, why the disease does not occur in mouse transgenic models; for these models, it is, more or less, the end of the story (at least as far as the induction of AD or rather the lack thereof is concerned): no matter how extensive the conventional overproduction of Aβ is, it would not reach the AD-causing level due to the “self-suppression” of its production under the ISR conditions and to the lack of the operational AβPP-independent Aβ production pathway. 

**Figure 9:**
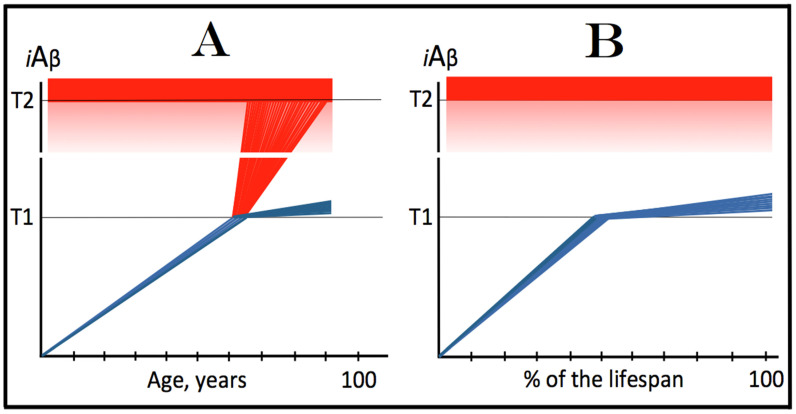
**Production of Aβ in the AβPP proteolytic pathway is either discontinued or severely suppressed in both humans and mouse under the neuronal ISR following the T1 crossing**. *i*Aβ: intraneuronal Aβ. *Blue lines*: levels of AβPP-derived *i*Aβ in individual neurons. *Red lines*: Levels of *i*Aβ generated independently of AβPP in individual neurons. *T*1 *threshold*: Cellular concentration of *i*Aβ that triggers the elicitation of the neuronal integrated stress response. *T*2 *threshold*: levels of *i*Aβ that trigger apoptosis or necroptosis of neuronal cells. *Gradient-pink boxes*: Range of concentrations of *i*Aβ that cause and propel AD pathology. *Red boxes*: Apoptotic zone; within this zone, the neurons have either undergone apoptosis or are dead. (**A**) Dynamics of the accumulation of *i*Aβ in the AD patient. AβPP-derived *i*Aβ accumulates physiologically as described in the main text and crosses the T1 threshold. This triggers the activation of PKR and/or HRI kinases, phosphorylation of eIF2α at its Ser51 residue, and the elicitation of the neuronal ISR. Under the ISR conditions, global cellular translation is reprogrammed and protein production is severely suppressed. It is presumed that the production of Aβ in the AβPP proteolytic pathway is no exception. Consequently, following the crossing of the T1 threshold by the AβPP-derived *i*Aβ, the rate of its accumulation substantially declines. Concurrently, the neuronal ISR activates and sustains the generation of *i*Aβ in the AβPP-independent pathway. It rapidly accumulates, reaches the AD pathology-causing range, and AD symptoms manifest. When it crosses the T2 threshold, the disease enters its end stage. (**B**) Dynamics of the accumulation of AβPP-derived *i*Aβ in transgenic mouse models. Both endogenous and exogenous AβPP-derived *i*Aβ accumulate physiologically via the cellular uptake of a fraction of secreted extracellular Aβ and the retention of *i*Aβ resulting from a fraction of C99 processed on the intraneuronal membranes. When the T1 threshold is crossed, the neuronal ISR is elicited as described above. The suppression of the production of Aβ in the AβPP proteolytic pathway, occurring in the context of the global suppression of the cellular protein synthesis, results in a substantial decline in the rate of the accumulation of AβPP-derived *i*Aβ. Since the AβPP-independent *i*Aβ generation pathway is inoperative in both wild mice and mouse models, the AD pathology-causing range of *i*Aβ concentrations would not be reached and AD symptoms would not occur.

In humans, this suppression of the production of AβPP-derived *i*Aβ presumably occurs as well and for the same reasons. But, in humans, it is the beginning of another story: the same neuronal ISR that suppresses the production of AβPP-derived Aβ (and, by extension, of AβPP-derived *i*Aβ) activates and enables the operation of and sustains the AβPP-independent pathway of the generation of the C100/C99 fragment of AβPP, the pathway, which drives Alzheimer’s disease. Figure 9 illustrates the suppression of the production of AβPP-derived Aβ under the neuronal ISR conditions as well as its consequences in both humans (panel A) and mouse models (panel B). Blue lines designate levels of AβPP-derived *i*Aβ in individual neurons, whereas red lines denote cumulative levels of AβPP-derived *i*Aβ and *i*Aβ generated independently of AβPP; T1 signifies neuronal ISR-eliciting levels of *i*Aβ.

## 25. Suppression of the Production of AβPP-Derived Aβ in Alzheimer’s Disease: An Impossibility, if Not an Apostasy, in Terms of the ACH, but a Triviality in the Framework of the ACH2.0 Theory of AD

Within the framework of the ACH theory of AD, a notion of the suppression, moreover, self-suppression, of the production of AβPP-derived Aβ in Alzheimer’s disease is an oxymoron. This notion is absolutely incompatible with the occurrence and progression of AD and is, therefore, an impossibility: there can be either this or AD, but never both simultaneously. This notion, indeed, contradicts the central tenet of the ACH, i.e., that AD is caused and driven by Aβ produced in the AβPP proteolytic pathway. If the notion of the self-suppression of the production of AβPP-derived Aβ in AD is correct, then the ACH is patently not. In sharp contrast, in the ACH2.0 theory of AD, the notion that the production of AβPP-derived Aβ is suppressed following the elicitation of the neuronal integrated stress response is only an inconsequential triviality. This is because the same neuronal ISR activates, upon its elicitation, the powerful AβPP-independent *i*Aβ production pathway (note: a possibility that this pathway yields not Aβ but rather only its precursor, C100/C99, as well as its consequences are discussed below). If the operation of the AβPP proteolytic pathway were to continue under the neuronal ISR conditions, its contribution would be marginal. Its postulated suppression, therefore, is inconsequential. The marginality of the *i*Aβ output of the AβPP proteolytic pathway in the context of the operational AβPP-independent *i*Aβ production pathway explains the complete inefficiency of drugs depleting extracellular Aβ or suppressing its production by the AβPP proteolysis in the treatment of AD. Indeed, the AβPP proteolytic pathway is important only prior to the commencement of AD: it provides AβPP-derived *i*Aβ that, upon crossing the T1 threshold, elicits the neuronal ISR and thus triggers the disease. This is why Aβ monoclonal antibodies and BACE inhibitors could be effective in the prevention of conventional AD but not in the treatment of the disease [4,6]. To briefly summarize the above, the suppression of the production of Aβ in the AβPP proteolytic pathway is inconsequential and largely irrelevant to the progression of Alzheimer’s disease, which is driven by the AβPP-independent C100/C99 generation pathway operating under the neuronal ISR conditions. 

## 26. Transgenic Mice Overexpressing Human AβPP Model Not AD but Only the Effects of the Neuronal ISR: Neurodegeneration and Cognitive Impairment Seen in Mouse Models Can Be Attributed to the Neuronal ISR Elicited by AβPP-Derived *i*Aβ

As discussed above, transgenic animal models cannot develop AD regardless of the extent of the exogenous overexpression of Aβ from human AβPP transgenes. On the other hand, these models exhibit a degree of both neurodegeneration and cognitive impairment. The question is, what are these effects? Are they relevant to Alzheimer’s disease? The answer is: they are relevant to AD to exactly the same extent that the neuronal ISR is. This is because these effects are a direct consequence of and can be attributed to the neuronal integrated stress response (with possible contribution of AACD or AACD-like condition; see below). The neuronal ISR occurs in both the neurons of transgenic mice overexpressing human AβPP and in the degenerating neurons of AD patients [145,146]. In both cases, mechanisms underlying the elicitation of the neuronal ISR are identical: the cellular uptake of a fraction of secreted extracellular Aβ and the intraneuronal retention of Aβ resulting from γ-cleavage of a fraction C99 support the accumulation of *i*Aβ to ISR-eliciting levels. When it crosses the T1 threshold, PKR and/or HRI are activated, eIF2α is phosphorylated, and the elicitation of the neuronal ISR ensues. Under the ISR conditions, the global cellular protein synthesis is severely suppressed. Over the long term, this inevitably results in cellular damage. That persistent ISR results in cellular degeneration was indeed shown in neuronal cells as well as in other cell types [147,148,149,150,151,152,153,154,155,156,157,158,159,160]. In the absence of AD (due to the inability of AβPP-derived *i*Aβ to reach the AD pathology-causing levels) in transgenic mice overexpressing human AβPP, this is the only plausible explanation for the observed neurodegeneration. Cognitive impairments seen in transgenic mice overexpressing human AβPP can also be attributed to the neuronal ISR, more precisely to the ISR-induced suppression of the total cellular protein synthesis. Indeed, the cognitive impairments observed in transgenic mouse models overexpressing Aβ have been explained in terms of the defects of neuronal plasticity, learning, and memory formation [147,148,149,150,151,152,153,154,155,156,157,158,159,160]. Each of these processes crucially depends on a new protein synthesis in neuronal cells; hence, the suppression of the global protein synthesis under the persistent AβPP-derived *i*Aβ-elicited neuronal ISR causes cognitive impairments [155,156,157,158,159,160]. The prevention of the neuronal ISR in transgenic mouse models overexpressing Aβ prevents cognitive impairment, and the suppression of the neuronal ISR, and thus the restoration of protein synthesis, abrogates cognitive impairments in the cases when they have already occurred [161,162,163,164,165,166,167,168,169].

It can be concluded, therefore, that transgenic mice overexpressing Aβ from human transgenes are not the AD models. No AD occurs in these mice. What they model, and model well, are the effects of the neuronal integrated stress response elicited by AβPP-derived *i*Aβ accumulated to sufficient levels and, possibly, the effects of AACD (or AACD-like condition) triggered by AβPP-derived *i*Aβ following the T^0^ crossing. These models emulate quite faithfully what happens in the run-up to AD in humans but only to a point. In transgenic mouse models, this point—the end stage—is the elicitation of the neuronal ISR; they stop there. In humans, this process goes one step (but a giant step) further by activating the AβPP-independent pathway of generation of C100/C99. This pathway drives the disease in humans but is inoperative in mice.

## 27. Validation of the Concept: (1) The Rate of the Endogenous or Exogenous Production of Aβ in Mouse Neuronal Cell Culture Would Drastically Decrease upon the Elicitation of the ISR

The present and two following sections consider possible approaches for the validation of the proposed concept positing that the production of Aβ in the AβPP proteolytic pathway is suppressed under the neuronal integrated stress response conditions. One approach is simply to measure the rate of either exogenous or endogenous production of AβPP, C99, and Aβ prior and following the elicitation, by different means, of the neuronal integrated stress response. These measurements should be carried out in mouse neuronal cells as an experimental system. In this system, the above measurements take advantage of the absence in mouse neurons of the operational AβPP-independent C100/C99 generation pathway. Indeed, in mouse neurons, Aβ (and C99) are produced solely in the AβPP proteolytic pathway. In contrast, in human neuronal cells, such measurements would be complicated if feasible at all. The reason for this is the activation, following the elicitation of the neuronal ISR of the AβPP-independent pathway that generates, in an ISR-compatible manner, C100/C99. Since C100 and Met-Aβ potentially derived from it are converted to C99 and Aβ (see above), it would be challenging to distinguish C99 and Aβ produced by the AβPP proteolysis from C99 and Aβ generated independently of AβPP. The only reliable determination that can be made in human neuronal cells is the relative rates of synthesis of AβPP (produced exogenously or endogenously) before and after the elicitation of the neuronal ISR. 

As for the means of the elicitation of the neuronal ISR, doing this by the exogenous overexpression of Aβ and the accumulation of *i*Aβ to sufficient levels (i.e., over the T1 threshold) would be a valid but complicated approach (for example, following the elicitation of the neuronal ISR and the suppression of its production, *i*Aβ levels could reverse-cross the T1 threshold; this would result in the abrogation of the ISR state and the resumption of the production of Aβ at the pre-ISR rate). Eventually, to better understand the dynamics of AβPP-derived Aβ prior and following the elicitation of the neuronal ISR, such approach would have to be employed but initially it is sufficient to elicit the neuronal ISR by any means, e.g., triggering mitochondrial dysfunction. Eliciting the neuronal ISR by several stressors would minimize the possibility that the suppression of the rate of production of molecules of interest is a side effect of the stressor rather than the result of the neuronal ISR. 

If the concept positing that the production of Aβ in the AβPP proteolytic pathway is suppressed under the neuronal integrated stress response conditions is valid, it can be anticipated to be reflected in the results of the above-described measurements. It should be emphasized that the ISR-triggered reduction in the rate of production of Aβ could be the effect of the suppression of the synthesis of AβPP but potentially it may also occur independently of it (further discussed below).

## 28. Validation of the Concept: (2) Overproduction of *i*Aβ in Mouse Neuronal Cells in the Presence of ISR Inhibitors Would Result in AD Pathology and the Formation of NFTs

Another approach to potentially validate the concept positing that the production of Aβ in the AβPP proteolytic pathway is suppressed under the neuronal integrated stress response conditions is to massively overexpress Aβ and to arrange for the accumulation of *i*Aβ in mouse neuronal cells in the presence of inhibitors of the ISR. The concept would be validated if this approach results in cellular AD pathology including the formation of NFTs. Potentially, the same approach can be employed with human neuronal cells. This is because with the ISR suppressed by the inhibitors, the AβPP-independent C100/C99 generating pathway would remain inoperative. The utilization of human neuronal cells would require a complete prevention of the ISR and could be technically challenging. The experimental goal of such an approach is to accumulate *i*Aβ to very high levels. This can be achieved in several ways. One is to massively overexpress human AβPP and allow *i*Aβ to accumulate physiologically, via the cellular uptake of a fraction of secreted extracellular Aβ and the intraneuronal retention of Aβ (*i*Aβ) resulting from the γ-cleavage on the intracellular membranes. This would be a slow process. The second approach is to overexpress only the C100/C99 fragment of human AβPP. The accumulation of exogenous *i*Aβ in this case would also be relatively slow because, due to the presence of the internal trans-membrane domain, the bulk of Aβ resulting from the γ-cleavage would be secreted. Third, the most effective approach would be to overexpress only human Aβ42. In this case, the entire production output of Aβ42 would remain intraneuronally and it would rapidly accumulate to high levels. If this approach were successful, i.e., if AD pathology and NFTs were observed, the control would be to reproduce the same experiment but in the absence of inhibitors of the ISR. Following the rate of accumulation of *i*Aβ (expected to be linear in the presence of ISR inhibitors but biphasic in their absence) could also confirm that the effect, if observed, is due to the extent of the accumulation of *i*Aβ.

The above approach might not be feasible because the neuronal ISR state could be requisite for the development of cellular AD pathology. In more precise terms, below (Section 35 and Section 36), we discuss the possibility that under the neuronal ISR conditions, γ-secretase is suppressed (or rather the production of its components is suppressed) and what drives Alzheimer’s disease is not *i*Aβ but rather C100/C99 generated independently of AβPP (a process enabled by the neuronal ISR, as discussed above). These aspects are addressed in the third validation approach described in the following section.

## 29. Validation of the Concept: (3) Overcoming the Neuronal ISR-Mediated Suppression of the Production of Aβ in Mouse Neuronal Cells via Utilization of IRES-Containing mRNAs Encoding C100 or Met-Aβ42

The approach to validate the concept positing that the production of Aβ in the AβPP proteolytic pathway is suppressed under the neuronal ISR conditions, discussed in the present section, considers the possibility that the neuronal integrated stress response state is requisite for the development of cellular AD pathology. The present approach emulates the conditions that prevail in human AD-affected neurons where the ISR enables and sustains the operation of the AβPP-independent pathway generating the C100/C99 fragment that may or may not be processed into *i*Aβ. Accordingly, in this approach, Met-*i*Aβ or the C100/C99 fragment of AβPP are expressed in mouse neuronal cells under the ISR conditions in a manner insensitive to the ISR and resistant to the phosphorylation of eIF2α. This entails the insertion of the IRES (internal ribosome entry site) element into the expression construct in such a way that translation of C100/C99 or of Met-Aβ42 (from a 3′-shortened AβPP construct) initiates from the AUG encoding Met671 of human AβPP. The neuronal ISR, essential in the design of this approach, can be elicited by a variety of stressors. In fact, the overexpression of C100/C99 or of Met-Aβ42 would also eventually trigger the ISR. With the former, it can take too long (since, following the γ-cleavage, rovided γ-secretase is not suppressed by the ISR, the bulk of Aβ would be secreted and the accumulation of *i*Aβ would depend on a slow process of cellular uptake) but, with the latter, it is feasible (because it would be retained intraneuronally and thus rapidly accumulate over the T1 threshold). However, since one of the aims of this approach is to compare effects of C99 and *i*Aβ, the elicitation of the ISR by other stressors would be best suited for the purpose. The same experiments but with expression constructs encoding conventionally translated C100/C99 or Met-Aβ42 would provide experimental controls. It is anticipated that these controls would be negative because conventional translation would be suppressed by the ISR but that the expression of C100/C99 or of Met-Aβ42 (or, possibly, both) would result in cellular AD pathology including the formation of NFTs. If successful results were obtained, the experiments described in the preceding section (at least those where only Aβ42 is expressed; to compare results with the expression of C100/C99 an inhibitor of γ-secretase possibly would have to be employed unless it is suppressed physiologically by the ISR as discussed below) would also complement those delineated in the present section as controls. It should be emphasized that the approach described in the present section is feasible with mouse neurons but not with human neuronal cells. This is because, in human neurons, the elicitation of the integrated stress response would activate the endogenous production of C100/C99 in the AβPP-independent pathway, which is inoperative in mouse neurons (see above). 

## 30. Approaches to Generate Adequate, Physiologically Relevant Transgenic Mouse Models of Alzheimer’s Disease 

### 30.1. Exogenous Generation of Met-Aβ42/Aβ42 and C100/C99 Under the Control of IRES Elements

The validation approach described in Section 28 above, namely, the overexpression of *i*Aβ, with the integrated stress response persistently suppressed, cannot be adopted into a transgenic mouse model of AD even if it is successful. This is because mice are not viable if the ISR is persistently suppressed [170] (further discussed in Section 36.3). However, if successful, this approach can be utilized in mouse neuronal cell-based models. On the other hand, if the overexpression of C100/C99 or *i*Aβ42 from mRNAs containing appropriately localized IRES elements, as described in the preceding section, is successful in simulating AD and driving AD pathology, including the formation of neurofibrillary tangles, such an approach, besides being utilized in mouse neuronal cell-based models, can be adopted in the development of an adequate transgenic mouse model of AD. Such a model would overexpress either human C100 or Met-Aβ42 under the control of IRES elements. In both C100 and Met-Aβ42, the N-terminal Met would eventually be removed post-translationally by one of aminopeptidases with broad specificity [128], thus converting them into C99 and Aβ42, respectively. Exogenous Aβ42 would be retained as *i*Aβ42 and rapidly accumulate and cross the T1 threshold. At this point, following the elicitation of the neuronal ISR, the production of the endogenous Aβ would be suppressed but that of the exogenous iAβ42 would continue unimpeded potentially driving AD pathology including the formation of NFTs. If, as argued below, AD is driven by C99, similar results could be seen in C100/C99-overexpressing mice.

The exogenously produced C99 fragment, on the other hand, would be cleaved by γ-secretase to generate Aβ, the bulk of which would be secreted. Exogenous C99-derived *i*Aβ would slowly accumulate via the cellular uptake of a fraction of secreted Aβ and the retention of *i*Aβ produced by the γ-cleavage on the intraneuronal membranes. Upon reaching and crossing the T1 threshold, it would trigger the elicitation of a neuronal-integrated stress response. At this point, the production of endogenous AβPP and of C99 and Aβ derived from it proteolytically would be potentially suppressed but the exogenous production of C100/C99 would continue, driven by the IRES element. Moreover, it would potentially remain intact due to the neuronal ISR-caused deficiency of γ-secretase. If AD is driven by C99, it can be expected that the accumulation of exogenous C99 would result in AD pathology including the formation of NFTs, provided it is produced in sufficient quantity (a parameter that can be regulated by the number of transgenes).

### 30.2. Expression of Human AβPP mRNA Eligible for Asymmetric Amplification

One or even both approaches described in Section 30.1 above may result in adequate transgenic mouse models developing the full spectrum of AD symptoms, including the formation of neurofibrillary tangles. These AD models, however, would not be entirely relevant physiologically. This is because the AβPP-independent pathway generating C100/C99 in human AD-affected neurons would remain inoperative in the above transgenic mouse models. In such a case, despite similarities in the final outcomes, the pathways leading to them may differ significantly, and such models could be not useful and even misleading in the development of the therapeutic strategies for AD. Therefore, the adequate and physiologically relevant transgenic mouse model of AD would be the one where the AβPP-independent pathway generating C100/C99 is rendered operational. To achieve this, two crucial requirements have to be satisfied. First, the integrated stress response has to be elicited and sustained in neuronal cells. Under the ISR conditions, the essential components of the RNA-dependent mammalian mRNA amplification process would be produced (see Section 21 above). The second requirement is to supply the eligible intact human AβPP mRNA to operate as the progenitor template (see Section 16, Section 17 and Section 18 above). Several approaches to accomplish this are described in detail elsewhere [7]. Arguably, the simplest approach is to modify, through genomic editing, human transgenes in the current transgenic mouse models in such a way as to restore the integrity of the 5′ terminus of transgenes and, accordingly, of exogenously produced human AβPP mRNA (for details, see [7]). With the first requirement, the elicitation of the neuronal integrated stress response, apparently satisfied in current transgenic models via the accumulation of exogenous AβPP-derived *i*Aβ (see above), and the AβPP-independent C100/C99 production pathway enabled by the availability of the amplification-eligible AβPP mRNA template, veritable AD would ensue. 

## 31. Why AβPP-Independent Production of C100/C99 in AD-Affected Human Neurons Is Insensitive to the ISR Conditions?

Under the conditions of the integrated stress response, the global cellular protein synthesis is severely suppressed. Very few protein species are exempted from and escape this suppression. In light of the above discussion, it is highly probable that AβPP is one of the subjects of this suppression. As discussed below, so may also be the activities involved in the proteolytic processing of AβPP. Yet, as posited in the ACH2.0 theory of AD, the production of C100/C99 in the AβPP-independent pathway proceeds unimpeded in AD-affected neurons under the ISR condition. What are the underlying reasons for such exclusion? The AβPP-independent C100/C99 generation is enabled and its operation is sustained by the ISR conditions. Only under such conditions are components essential for the operation of this pathway (and not present under the regular, non-ISR conditions) produced. In three of the four mechanisms potentially enabling the AβPP-independent production of C100, its translation occurs from severely 5′-truncated AβPP mRNA, and in two of the three mechanisms, these truncated mRNAs are cap-less (discussed above). The utilization of uncapped mRNAs is one of the hallmarks of the ISR-induced translation. Moreover, human AβPP mRNA may contain IRES or other elements enabling initiation of translation from the AUG encoding Met671 of AβPP but these elements can be masked and, thus, not functional within the intact mRNA molecule. In this context, truncation of the mRNA molecule unmasks the translation-enabling element. The possible, in fact plausible (discussed above), chimeric asymmetric amplification of human AβPP mRNA opens up additional possibilities. In this mechanism, not only is the end product mRNA 5′-truncated, but it also contains an additional 5′-terminal segment (the 3′-terminal fragment of the antisense AβPP mRNA), which may harbor IRES or other translation-enabling element. Moreover, as described above, the end product of the chimeric amplification of human AβPP mRNA is heavily modified. Ostensibly, these modifications prevent the re-annealing of sense and antisense RNA strands following their separation, but they may also facilitate cap-independent translation, a possibility supported by the observation that a single m6A modification within the 5′UTR of an mRNA can do precisely this [171]. Interestingly, the options discussed above reduce the possibility of the internal initiation of translation of the intact AβPP mRNA from the AUG encoding Met 671. This is because, in this case, neither IRES or other regulatory elements can be unmasked nor can they be added to mRNA molecules. 

## 32. The Plausibility of Human Neuronal Cell-Based AD Models

As discussed in the preceding sections above, the existing current transgenic animal models of AD are patently inadequate, and the proposed adequate and physiologically relevant transgenic mouse models of AD remain to be developed. This situation creates an acute need for an acceptable experimental model of AD. In fact, a prototype of such a model exists and can be significantly improved upon. Moreover, arguably, this can be the best possible model, albeit with some limitations. The model under discussion is based on human neuronal cells. Due to their human origin, these cells possess the mechanism capable of the AβPP-independent production of C100 (this mechanism is present in human neuronal cells by definition, as one of the tenets of the ACH2.0 theory, even if its nature remains for the moment uncertain). Therefore, to generate a human neuronal cell-based AD model, all that remains is to activate the AβPP-independent C100 generating pathway, and cellular AD pathology, including the formation of neurofibrillary tangles, will ensue. The plausibility of this approach was, in fact, inadvertently established experimentally as follows.

Several years ago, a human neuronal cell-based model was designed and constructed by Choi and coworkers [172]. The objective of this model was to maximize the extracellular deposition of secreted Aβ in order to maximize the occurrence and extent of cellular AD pathology. The major innovation of this study was the cultivation and differentiation of human neural progenitor cells in matrigel, a semi-solid medium, with the intention of preventing the diffusion of secreted extracellular Aβ. Besides cultivating cells in matrigel, measures were employed to maximize the production of amyloidogenic variants of Aβ. To this end, human AβPP carrying two FAD-causing mutations, Swedish (K670N/M671L) and London (V717I), was overexpressed exogenously in human neural progenitor cells. Concurrently, and from the same lentivirus polycistronic expression construct, PSEN1 with the ΔE9 FAD mutation was also overexpressed. Following the introduction of the expression construct, cells were differentiated into neurons in matrigel medium. This approach was proven to be very fruitful: for the first time, the formation of neurofibrillary tangles was observed in an experimental model. The authors interpreted their observations in terms of the ACH and concluded [172] that the observed cellular pathology was due to the increased deposition of Aβ, thus affirming (in their perception) the Alzheimer’s disease-causing potential of extracellular Aβ. 

The interpretation of the same observations in terms of the ACH2.0 theory of AD is distinctly different. Both the London and ΔE9 FAD mutations significantly increase the production and, consequently, secretion of the Aβ42 variant. On the other hand, the Swedish mutation substantially elevates the fraction of C99 undergoing the γ-secretase cleavage on the intraneuronal membranes, thus leading to the retention of the resulting Aβ as *i*Aβ. Not only is the Aβ42 variant more toxic within the cell due to its propensity to aggregate; when secreted and retained in the vicinity of the cell due to utilization of matrigel, it is internalized twice as efficiently as other Aβ variants. Thus, this model maximizes the influx of exogenously produced AβPP-derived *i*Aβ by increasing both the retention and cellular uptake of *i*Aβ42. Due to the high rate of its influx, exogenous *i*Aβ42 rapidly accumulates, reaches the T1 threshold and triggers the elicitation of the integrated stress response. The neuronal ISR, in turn, provides the essential components and sustains the operation of the AβPP-independent C100 generation pathway enabled by the availability of the endogenous AβPP mRNA. It is this pathway, rather than extracellular Aβ, that drives (via the production of C99 or *i*Aβ) AD pathology and is responsible for the formation of NFTs.

The human neuronal cell-based model described above can be substantially streamlined and simplified. The essence of such a model is the elicitation of the persistent integrated stress response. This can be achieved in two conceptually distinct ways. One is to accumulate *i*Aβ over the T1 threshold. This is exactly what was done in the above described model (in a convoluted manner), but it can be performed much more simply and efficiently by expressing only *i*Aβ42 (more precisely, Met-Aβ42 that will be processed into Aβ42). Due to the lack of the trans-membrane domain, it would be retained intraneuronally, rapidly accumulate, cross the T1 threshold, and trigger the elicitation of the ISR. In turn, the ISR would activate and sustain the endogenous AβPP-independent production of C100; the occurrence of cellular AD pathology and formation of NFTs would follow. Such a model would faithfully reflect and emulate conventional Alzheimer’s disease.

The second way to generate the human neuronal cell-based model of AD is to elicit the persistent sustainable integrated stress response by utilizing stressors distinct from *i*Aβ. Within the framework of the ACH2.0, the origin of the neuronal ISR is important but irrelevant for the activation of the AβPP-independent C100 generation pathway. The neuronal ISR, sustainably elicited in any manner, would activate the endogenous production of C100 independently of AβPP. When C100 or (if it is processed into Aβ) the *i*Aβ derived from it would reach a certain critical threshold, the T1, it would continuously propagate the ISR, and thus perpetuate its own production in the AβPP-independent pathway. It would also drive cellular AD pathology, including the formation of NFTs. Such a model would faithfully reflect and simulate unconventional Alzheimer’s disease [8,9].

## 33. Assessing the Origins and Consequences of the Neuronal ISR-Mediated Suppression of the Generation of AβPP-Derived Aβ

### 33.1. Assessing the Origins of the Neuronal ISR-Mediated Suppression of the Generation of AβPP-Derived Aβ: (1) Production of AβPP?

Provided that the production of AβPP-derived Aβ is suppressed under the neuronal integrated stress response conditions, what could the reason for this be? The answer, of course, is the suppression of the production of either the precursor, AβPP, or of the enzymes involved in the processing of the precursor and the intermediates, or both. The present subsection considers the consequences of the neuronal ISR-caused suppression of the production of AβPP in the context of the proteolytic processing of AβPP into Aβ remaining fully operational. One possibility is that they would be, in fact, the same as those depicted in Figure 9 (Section 24) for both mouse and human neurons. In both cases, AβPP-derived *i*Aβ would accumulate at a certain constant rate until crossing the T1 threshold and triggering the elicitation of the integrated stress response. As a result, in both mouse and human neurons, the rate of accumulation of AβPP-derived neurons would significantly decrease. In human (but not in mouse) neurons, the ISR will enable and sustain the operation of the AβPP-independent C100 generation pathway, which can drive AD pathology.

**Figure 10:**
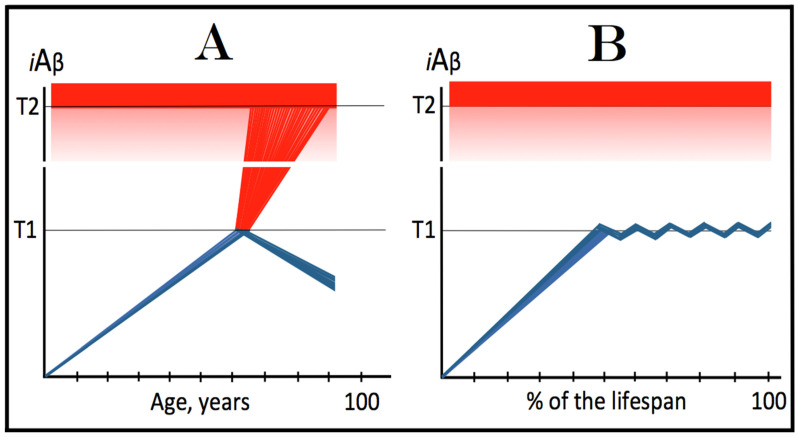
**Inhibition of the translation of AβPP as the potential origin of the neuronal ISR-mediated suppression of the production of AβPP-derived *i*Aβ**. *i*Aβ: intraneuronal Aβ. *Blue lines*: levels of AβPP-derived *i*Aβ in individual neurons. *Red lines*: levels of *i*Aβ generated independently of AβPP in individual neurons. *T*1 *threshold*: cellular concentration of *i*Aβ that triggers the elicitation of the neuronal integrated stress response. *T*2 *threshold*: levels of *i*Aβ that trigger apoptosis or necroptosis of neuronal cells. *Gradient-pink boxes*: range of concentrations of *i*Aβ that cause and propel AD pathology. *Red boxes*: apoptotic zone; within this zone the neurons have either undergone apoptosis or are dead. (**A**) Dynamics of the accumulation of *i*Aβ in AD patients. AβPP-derived *i*Aβ accumulates physiologically (as described in the main text) and crosses the T1 threshold. This triggers the activation of PKR and/or HRI kinases, phosphorylation of eIF2α at its Ser51 residue, and the elicitation of the neuronal ISR. Under the ISR conditions, global cellular translation is reprogrammed and protein production is severely suppressed. It is presumed that the production of AβPP and, consequently, of Aβ in the AβPP proteolytic pathway is no exception. Consequently, following the crossing of the T1 threshold by the AβPP-derived *i*Aβ, the rate of its accumulation is reversed. Concurrently, the neuronal ISR activates and sustains the generation of *i*Aβ in the AβPP-independent pathway. Thus, although the levels of AβPP-derived *i*Aβ decrease below the T1 threshold, the ISR state, maintained now by *i*Aβ produced independently of AβPP, remains in effect. (**B**) Dynamics of the accumulation of AβPP-derived *i*Aβ in transgenic mouse models. Both endogenous and exogenous AβPP-derived *i*Aβ accumulate physiologically via the cellular uptake of a fraction of secreted extracellular Aβ and the retention of *i*Aβ resulting from a fraction of C99 processed on the intraneuronal membranes. When the T1 threshold is crossed, the neuronal ISR is elicited as described above. The suppression of the production of AβPP and, consequently, of Aβ in the AβPP proteolytic pathway, in the context of the global suppression of the cellular protein synthesis, results in reversing the rate of the accumulation of AβPP-derived *i*Aβ. Since the AβPP-independent *i*Aβ generation pathway is inoperative in both wild mice and mouse models, the reverse crossing of the T1 threshold restores normal (i.e., non-ISR) conditions. The production of AβPP and, consequently, the influx of AβPP-derived *i*Aβ resumes. Eventually, the latter crosses the T1 threshold, triggers the re-elicitation of the neuronal ISR, and the oscillating cycle repeats.

Another possibility is that the suppression of the production of AβPP following the elicitation of the neuronal ISR is so substantial that the accumulation of AβPP-derived *i*Aβ would be reversed and its levels would decline. This possibility is illustrated in Figure 10. In both human and mouse neurons, AβPP-derived *i*Aβ reverse-crosses the neuronal ISR-eliciting T1 threshold. As a result, it can no longer support the ISR state. In human neurons, the reverse crossing of the T1 threshold by AβPP-derived *i*Aβ is inconsequential because the ISR is sustained by the operation of the AβPP-independent C100 generation pathway (panel A of Figure 10); consequently, this pathway remains operational and drives AD. In mouse neurons, on the other hand, the reverse crossing of the T1 threshold by AβPP-derived *i*Aβ creates an interesting situation, as shown in panel B of Figure 10. Since the ISR state is reversed and no longer in effect, the “normal” production/uptake of AβPP-derived *i*Aβ commences, and it accumulates at the pre-T1 crossing rate. Eventually, it crosses the T1 threshold and re-elicits the neuronal ISR. This causes the suppression of the production of Aβ and the reversal of the accumulation of AβPP-derived *i*Aβ. The T1 threshold is reverse-crossed again, and the oscillating cycle is repeated. AβPP-derived *i*Aβ would not reach the AD-causing range and the disease would not occur.

### 33.2. Assessing the Origins of the Neuronal ISR-Mediated Suppression of the Generation of AβPP-Derived Aβ: (2) Supply of BACE Enzymes?

This subsection analyses the effect of the neuronal ISR-mediated suppression of the production of BACE enzymes in the context of the “normal”, i.e., ISR-unaffected, generation of AβPP and γ secretase complex. In this scenario AβPP-derived *i*Aβ accumulates via the internalization of a fraction of secreted Aβ and the intraneuronal retention of Aβ (*i*Aβ) resulting from the γ-cleavage of a fraction of the C99 fragment of AβPP on intracellular membranes. When it crosses the T1 threshold, the neuronal ISR is elicited and the production of the β-site cleaving enzymes is suppressed. Due to the deficit of BACE enzymes, the rate of the processing of AβPP and, consequently, that of the production of Aβ and accumulation of AβPP-derived *i*Aβ would decline. The resulting situation would be, in fact, the same as those depicted in Figure 9 and Figure 10 and described above. Whereas the dynamics of *i*Aβ would be significantly affected in mouse neurons, the effect would be largely inconsequential in AD-affected human neurons. This is because the AβPP-independent C100 generation pathway would remain operational. The processing (γ-cleavage) of C100/C99 produced independently of AβPP would produce a steady influx of *i*Aβ (provided the availability of γ-secretase is not affected by the neuronal ISR; see below).

### 33.3. Assessing the Origins of the Neuronal ISR-Mediated Suppression of the Generation of AβPP-Derived Aβ: (3) Availability of γ-Secretase?

The present subsection considers the consequences of the neuronal ISR-triggered suppression of the production of components of the γ-secretase complex, with other elements of the AβPP proteolytic pathway remaining operational. In mouse neuronal cells, the accumulation of AβPP-derived iAβ to the levels exceeding the T1 threshold would result in the activation of the PKR and/or HRI kinases, phosphorylation of eIF2α at its Ser51 residue, and the elicitation of the integrated stress response. In the scenario under discussion, the production of constituents of the γ-secretase complex is suppressed and even if other components of the AβPP proteolytic pathway are operational, the rate of the production of AβPP-derived iAβ and that of its accumulation would decline. The dynamics of iAβ, therefore, would be the same as depicted in Figure 9 (panel B) and Figure 10 (panel B). The neuronal ISR-triggered reduction in the rate of γ-cleavage would result in the accumulation of C99. Since it would be produced only by the AβPP proteolysis and its accumulation, following the elicitation of the ISR, would start at a low baseline, this is likely to be inconsequential.

In human neuronal cells, on the other hand, the situation would be radically different. As in mouse neurons, the accumulation of AβPP-derived iAβ over the T1 threshold would trigger the elicitation of the integrated stress response. As in mouse neurons, the ISR would suppress the rate of the γ-cleavage and lead to a reduction in the rate of accumulation of AβPP-derived iAβ, as shown in Figure 9 (panel A) and Figure 10 (panel A) and to a relatively slow accumulation of C99 produced by the proteolysis of AβPP. But in human neurons the ISR would also activate the powerful AβPP-independent C100/C99 production pathway. Due to the deficit of γ-secretase, C99 produced in this pathway would rapidly accumulate and potentially become the predominant Aβ-containing protein species in AD-affected cells. As discussed in the following section, this would result in the fundamental reassessment of the nature and identity of the driver of Alzheimer’s disease.

## 34. Alzheimer’s Disease Is Possibly Driven by C100/C99 Generated in the AβPP-Independent Pathway

**Figure 11:**
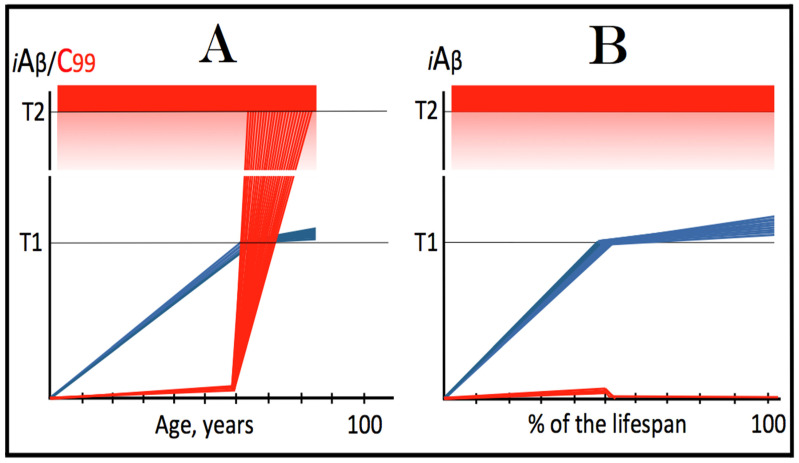
**The neuronal ISR-caused deficiency of γ-secretase as the basis of the suppression of the production of AβPP-derived *i*Aβ**. *i*Aβ: intraneuronal Aβ. *C99*: The fragment of AβPP produced either by the proteolysis of AβPP or independently of AβPP. *Blue lines*: levels of AβPP-derived *i*Aβ in individual neurons. *Red lines*: levels of the C99 fragment of AβPP in individual neurons. ***T*1** *threshold*: cellular concentration of *i*Aβ or of C99 that triggers the elicitation of the neuronal integrated stress response. ***T*2** *threshold*: levels of *i*Aβ or C99 that trigger apoptosis or necroptosis of neuronal cells. *Gradient-pink boxes*: range of concentrations of C99 or *i*Aβ that cause and propel AD pathology. *Red boxes*: apoptotic zone; within this zone, the neurons have either undergone apoptosis or are dead. (**A**) Dynamics of the accumulation of *i*Aβ and C99 in the AD patient. AβPP-derived *i*Aβ accumulates physiologically, as described in the main text, and crosses the T1 threshold. This triggers the activation of PKR and/or HRI kinases, phosphorylation of eIF2α at its Ser51 residue, and the elicitation of the neuronal ISR. Under the ISR conditions, global cellular translation is reprogrammed and protein production is severely suppressed. It is presumed that the production of the components of the AβPP proteolytic pathway in general and that of γ-secretase in particular is no exception. Consequently, following the crossing of the T1 threshold by the AβPP-derived *i*Aβ, the rate of its accumulation declines (as shown) or is reversed. Concurrently, the neuronal ISR activates and sustains the operation of the AβPP-independent pathway, which generates, due to the deficiency of γ-secretase, C99. The latter rapidly accumulates, crosses the T1 threshold, reaches the AD pathology-causing range, and eventually crosses the T2 threshold. It both drives the disease and propagates the neuronal ISR state, thus perpetuating its own production. (**B**) The dynamics of the accumulation of AβPP-derived *i*Aβ in transgenic mouse models. Both endogenous and exogenous AβPP-derived *i*Aβ accumulate physiologically via the cellular uptake of a fraction of secreted extracellular Aβ and the retention of *i*Aβ resulting from a fraction of C99 processed on the intraneuronal membranes. When the T1 threshold is crossed, the neuronal ISR is elicited as described above. The suppression of the production of Aβ in the AβPP proteolytic pathway in the context of the global inhibition of cellular protein synthesis results in a substantial decline in the rate of the accumulation of AβPP-derived *i*Aβ. Since the AβPP-independent C99 generation pathway is inoperative in both wild mice and mouse models, the AD pathology-causing range would be reached neither by *i*Aβ nor by C99, and AD symptoms would not occur. If the rate of AβPP-derived *i*Aβ were reversed under the neuronal ISR conditions, the oscillating pattern of *i*Aβ levels shown in Figure 10B would result.

Due to the global suppressing effect of the neuronal integrated stress response on cellular protein synthesis, it is likely that in the neurons where the ISR has been elicited (by AβPP-derived *i*Aβ at over-T1 levels or otherwise), all components of the AβPP production and proteolysis are suppressed. The outcome of such suppression would be defined by the inhibition of the most consequential component, γ-secretase, more precisely, its availability, and thus the rate of the γ-cleavage of the C99 fragment of AβPP. Consequences of the neuronal ISR-mediated suppression of AβPP production and/or proteolysis are illustrated in Figure 11. Panel A depicts these consequences in human neurons. They are both drastic and far-reaching. AβPP-derived *i*Aβ accumulates over the T1 threshold and triggers the elicitation of the neuronal ISR. Consequently, the production of AβPP and/or its proteolysis are suppressed. The rate of accumulation of AβPP-derived *i*Aβ declines. Even if it were reversed, the reduction would occur in a linear rather than oscillating fashion because the neuronal ISR state would persist due to the activity of the neuronal ISR-enabled AβPP-independent C100 generation pathway. With the activity of γ-secretase suppressed under the neuronal ISR conditions, C100 would be processed into C99 (by one of cellular aminopeptidases with broad specificity, as described above), but its bulk, if not its entirety, would not be converted into *i*Aβ. Therefore, were this the case, Alzheimer’s disease is not driven (or not only driven) by *i*Aβ produced independently of AβPP, as we proposed previously (e.g., Figure 2 above, see also [1,2,3,4,5,6,7,8,9]), but mainly, possibly exclusively, by C100/C99 generated in the AβPP-independent pathway. C99, produced in the powerful AβPP-independent pathway rapidly accumulates (red lines), reaches the AD pathology-causing range (pink gradient box), crosses the T2 threshold, triggers apoptosis or necroptosis, and the disease enters its end stage.

The consequences of the neuronal ISR-caused suppression of the production and/or proteolysis of AβPP in general, and of the γ-cleavage of the C99 fragment in particular, in the neurons of transgenic mouse model are shown in panel B of Figure 11. They are broadly the same as those shown in panel B of Figure 9. AβPP-derived *i*Aβ accumulates via the two mechanisms discussed above, reaches the T1 threshold, and triggers the elicitation of the neuronal ISR. Following the ISR-caused suppression of the production and/or proteolysis of AβPP, the rate of accumulation of AβPP-derived *i*Aβ significantly declines. If it were reversed, the levels of AβPP-derived *i*Aβ would reverse cross the T1 threshold, and the outcome would be similar to that shown in panel B of Figure 10. The crucial difference between human and mouse neurons is that whereas in the former the ISR activates and sustains the operation of the AβPP-independent C100 generation pathway, in the latter this pathway remains inoperative. AβPP-derived *i*Aβ and C99 reach neither the AD pathology-causing range, nor the T2 threshold, and AD does not occur.

The notion that C100/C99 generated independently of AβPP drives AD pathology raises an important question: Is it “qualified” to perform this task? As discussed elsewhere [2], any potential driver of AD pathology, conceivably enabled and sustained by the neuronal ISR, must satisfy two key requirements. One, it should propagate the neuronal ISR and thus to ensure the perpetuation of its own production. Two, it must be sufficiently toxic to cause, upon reaching certain cellular levels, neurodegeneration underlying the progression of AD. Does C100/C99 fulfill these requirements? The answer is, apparently, the affirmative one. C99 was shown to be capable of triggering the ISR in a number of ways. Two of those are the elicitation of the ER (endoplasmic reticulum) stress and the causation of mitochondrial dysfunction [173,174]; both conditions ultimately result, via the phosphorylation of eIF2α, in the integrated stress response. As for the cellular toxicity, increased levels of C99 were demonstrated to interfere, via its interaction with mitochondria-associated ER membranes (MAM), with lipid metabolism, resulting in the loss of lipid homeostasis and mitochondrial distress [173,174,175]. Furthermore, the intraneuronal aggregation of C99 was shown to induce lysosomal-autophagic pathology [176]. Importantly, it was shown that, in Alzheimer’s disease, C99 accumulates selectively in vulnerable neurons (and not in resistant brain areas) and that its levels correlate well with the degree of cognitive impairment of AD patients [177,178]. These considerations are consistent with the notion that γ-secretase and, consequently, γ-cleavage are suppressed under the neuronal ISR conditions. They also strongly suggest that Alzheimer’s disease is driven by C100/C99 generated in the AβPP-independent pathway, a highly substantial alteration of our comprehension of the disease. In this context, the “substance X” proposed previously as the driver of AD pathology [2] can be identified with certainty as C99 generated independently of AβPP.

## 35. C100/C99 as the Driver of AD Pathology: Experimental Assessment

The concept of C100/C99 being the driver of AD pathology can be assessed experimentally in both mouse and human neuronal cells. In either case, it has to be ascertained that human C99 is substantially overexpressed and that it is not being cleaved at the γ-site and thus processed into *i*Aβ. One way to do this is to overexpress conventional mRNA-encoded human C100 in mouse neuronal cells in the presence of both ISR and γ-secretase inhibitors. The latter would prevent the γ-cleavage and ensure the integrity of C99 whereas the former would prevent the elicitation of the neuronal ISR by C99 accumulated to sufficient levels. This is important because, if the ISR were elicited in this experiment, it would suppress translation of C100/C99 from a conventional capped mRNA. The occurrence in such an experiment of AD pathology, especially the formation of neurofibrillary tangles, would confirm the concept.

It is possible, however, that the neuronal ISR plays a certain role in the occurrence of AD pathology, which is unrelated to C99. To account for such a possibility, the assessment of the concept, also utilizing mouse neuronal cells, can be carried out as follows. In this experiment, human C100/C99 is overexpressed by translation from an unconventional IRES element-containing mRNA in mouse neuronal cells, where the ISR has been sustainably elicited by a relevant stressor in the presence of γ-secretase inhibitors. In this approach, the elicitation of the neuronal ISR would provide the appropriate context, the employment of the IRES element-containing C100 encoding would ensure the uninterrupted (by the ISR-mediated suppression) production of C100/C99, and the presence of γ-secretase inhibitors would ascertain that C99 is not processed further into *i*Aβ. 

The conclusive experiment designed to assess the proposed concept would be the one utilizing human neuronal cells. In this experiment, it is not necessary to overexpress C100/C99 exogenously because it would happen physiologically and endogenously upon the elicitation of the integrated stress response. In this approach, the neuronal ISR is sustainably elicited by a relevant stressor in the presence of γ-secretase inhibitors. Consequently, the AβPP-independent C100/C99 generation pathway is activated. The proposed concept assumes that γ-secretase activity is suppressed under the ISR conditions. Nevertheless, to ascertain that this is the case, γ-secretase inhibitors are included in the assay. It is anticipated that C99, accumulated to sufficient levels, would trigger the occurrence and progression of cellular AD pathology including the formation of NFTs. 

It is important to note the following. Sections below discuss potential therapeutic strategies for Alzheimer’s disease and aging-associated cognitive decline. To simplify the discussion and the Figures, we consider cases where the production of AβPP-derived *i*Aβ is not affected by the neuronal ISR. This is because if this were the case, the conclusions of our analysis would not be affected. On the other hand, the potential ISR-mediated inhibition of the production of BACE enzymes would be of fundamental importance for therapeutic approaches. Therefore, the therapeutic strategies discussed below take into consideration and address the possibility that the production of BACE enzymes is suppressed under neuronal integrated stress response conditions. Finally, since the radical possibility that C99 generated independently of AβPP is not cleaved by γ-secretase and remains intact has not yet been proven, the therapeutic strategies under discussion assume that in AD-affected neurons, C100/C99 generated in the AβPP-independent pathway is processed into *i*Aβ; the potential impact (or rather the lack thereof) on therapeutic strategies of C99 remaining unprocessed and actually driving the disease is addressed in Section 52 below.

## 36. Long-Term Administration of the ISR Inhibitors Could Be Beneficial Both in the Prevention and Treatment of AD but Is Not Feasible 

The following sections consider therapeutic applications stemming from the vision of AD presented above. One such application is the suppression of the integrated stress response. The neuronal ISR is presumed to provide components essential for the activity of the AβPP-independent C100/C99 generation pathway, the driver of the disease. Consequently, it can be reasoned that if the former were prevented or suppressed, the later would be precluded or rendered inoperative. 

### 36.1. Long-Term Suppression of the Integrated Stress Response in the Prevention of Conventional Alzheimer’s Disease

**Figure 12:**
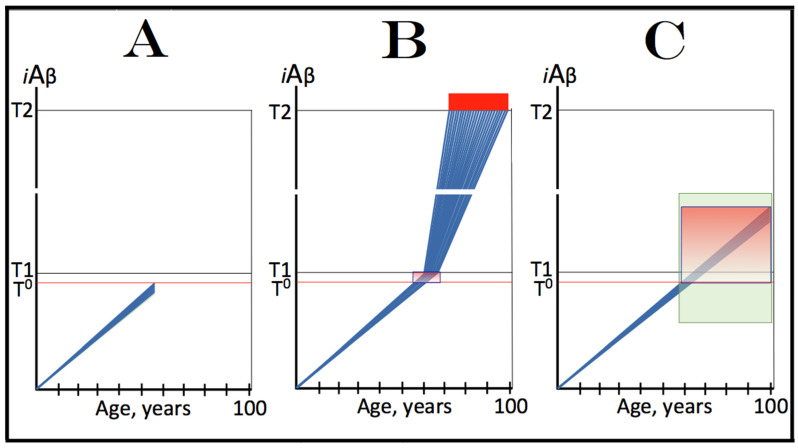
Long-term suppression of the integrated stress response in the prevention of conventional Alzheimer’s disease. *i*Aβ: intraneuronal Aβ. *Blue lines*: levels of *i*Aβ in individual neurons. ***T*^0^** *threshold*: the level of AβPP-derived *i*Aβ above which the neuronal damage manifesting as AACD commences. ***T*1** *threshold*: cellular concentration of *i*Aβ that triggers the elicitation of the neuronal integrated stress response. ***T*2** *threshold*: levels of *i*Aβ that trigger apoptosis or necroptosis of neuronal cells. *Red box*: apoptotic zone; within this zone, the neurons have either undergone apoptosis or are dead. *Green box*: duration of the inhibition of the ISR. *Gradient-pink boxes*: the range of AβPP-derived *i*Aβ concentrations between the T^0^ and T1 thresholds (provided T^0^ < T1), a zone of the occurrence of AACD; note that AACD can persist over the T1 threshold if the commencement of AD is prevented. (**A**) Initial state of AβPP-derived *i*Aβ levels in individual neurons; they are below both T^0^ and T1. (**B**) Evolution of the initial state in the untreated person. AβPP-derived *i*Aβ reaches the T^0^ threshold and AACD commences. With the crossing of the T1 threshold the neuronal ISR is elicited, the AβPP-independent C100/C99 generation pathway activated, and AD commences. At this point AACD morphs into AD. *i*Aβ produced independently of AβPP accumulates, reaches the T2 threshold and the disease enters the end stage. (**C**) Evolution of the initial state in the presence of ISR inhibitors. With the crossing of the T^0^ threshold by AβPP-derived *i*Aβ AACD commences. When the T1 threshold is crossed the neuronal ISR cannot be elicited and the AβPP-independent C100/C99 generation pathway remains inactive. No AD occurs, but AACD persists for the duration of the treatment.

To prevent the AβPP-derived *i*Aβ-triggered elicitation of the neuronal integrated stress response, the administration of its inhibitor should start prior to the T1 crossing. When the T1 is crossed, in the presence of ISR inhibitors, the consequence would be unremarkable. The AβPP-independent C100/C99 generation pathway would remain inoperative due to the lack of the required components, and the accumulation of AβPP-derived *i*Aβ would proceed at the slow pre-T1 crossing rate. Its levels would not reach the AD pathology-driving range, and no AD symptoms would manifest. This therapeutic strategy is illustrated in Figure 12. Panel A depicts the initial state of AβPP-derived *i*Aβ concentrations in the neurons of a healthy individual that would, if left untreated, eventually develop AACD and, subsequently, AD, as shown in panel B. Panel C of Figure 12 depicts the evolution of the initial state in the presence of a drug capable of suppressing the ISR (green box). The neuronal ISR is not elicited, the AβPP-independent C100/C99 generation pathway is not activated, and AD does not occur for the duration of the treatment. Note that the preventive suppression of the neuronal ISR would not affect the progression of AACD. Indeed, if AβPP-derived *i*Aβ were to cross the T^0^ threshold, AACD would be triggered despite the presence of ISR suppressors, as shown in panels B and C of Figure 12. In panel B, AACD morphs into AD with the crossing of the T1 threshold. Since AACD cannot morph into AD in the presence of ISR inhibitors (panel C), it would persist for the rest of the lifespan (provided the drug is administered for the lifetime). 

### 36.2. Long-Term Suppression of the Integrated Stress Response in the Treatment of Conventional Alzheimer’s Disease

**Figure 13:**
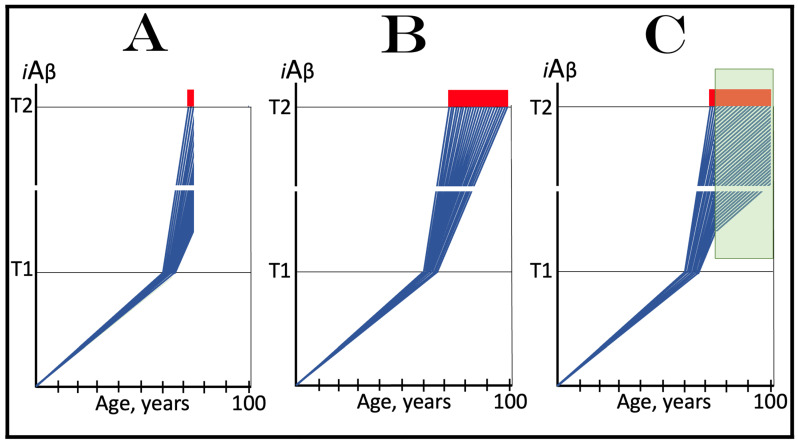
Long-term suppression of the integrated stress response in the treatment of conventional Alzheimer’s disease. *i*Aβ: intraneuronal Aβ. *Blue lines*: levels of *i*Aβ in individual neurons. ***T*1** *threshold*: cellular concentration of *i*Aβ that triggers the elicitation of the neuronal integrated stress response. ***T*2** *threshold*: levels of *i*Aβ that trigger apoptosis or necroptosis of neuronal cells. *Red box*: apoptotic zone; within this zone, the neurons have either undergone apoptosis or are dead. *Green box*: duration of the inhibition of the ISR. (**A**) Initial state of AβPP-derived *i*Aβ levels in individual neurons. AβPP-derived *i*Aβ has already crossed the T1 threshold in all affected neurons of the AD patient. Consequently, the neuronal integrated stress response is elicited, the AβPP-independent C100/C99 generation pathway is activated, and *i*Aβ produced independently of AβPP rapidly accumulates. The apoptosis-triggering T2 threshold is crossed in a fraction of the neurons, but the bulk of the affected neurons remain sub-T2. (**B**) Evolution of the initial state in the untreated individual. The AβPP-independent C100/C99 generation pathway remains operational, the accumulation of *i*Aβ produced independently of AβPP continues uninterrupted, and more neurons cross the T2 threshold, leading to the end stage of the disease. *i*Aβ produced independently of AβPP accumulates, reaches the T2 threshold, and the disease enters the end stage. (**C**) Evolution of the initial state in the presence of ISR inhibitors. With the supply of essential components interrupted, the operation of the AβPP-independent C100/C99 generation pathway ceases. However, due to the influx of AβPP-derived *i*Aβ, the levels of total *i*Aβ continue to increase, although at the slow, pre-T1 crossing rate. Levels of *i*Aβ produced independently of AβPP continue to cross the T2 threshold, and the progression of the disease would persist albeit at a decreased rate for the duration of the treatment.

In this scenario, inhibitors of the integrated stress response are administered when AβPP-derived *i*Aβ has already crossed the T1 threshold in all affected neurons of the AD patient. Consequently, the neuronal integrated stress response is elicited, the AβPP-independent C100/C99 generation pathway is activated, and *i*Aβ produced independently of AβPP rapidly accumulates. The apoptosis-triggering T2 threshold is crossed in a fraction of the neurons, but the bulk of the affected neurons remain sub-T2. The above picture, shown in panel A of Figure 13, constitutes the initial state of *i*Aβ levels in the affected individual neurons of the AD patient. The evolution of this initial state in the untreated patient is depicted in panel B of Figure 13. The AβPP-independent C100/C99 generation pathway remains operational, the accumulation of *i*Aβ produced independently of AβPP continues uninterrupted, and more neurons cross the T2 threshold leading to the end stage of the disease. The evolution of the initial stage in the presence of ISR inhibitors (green box), as shown in panel C of Figure 13, is different. With the supply of essential components interrupted, the operation of the AβPP-independent C100/C99 generation pathway ceases. However, due to the influx of AβPP-derived *i*Aβ, the levels of total *i*Aβ would continue to increase, although at the slow, pre-T1 crossing rate. The neurons would continue to cross the T2 threshold, and the progression of the disease would persist albeit at a decreased rate for the duration of the treatment.

### 36.3. Long-Duration Administration of ISR Inhibitors Is Unfeasible

Both preventive and curative applications of ISR inhibitors described above are beneficial. Both, however, require the long-duration administration of the drug. This is, apparently, not feasible. The ISR is one of the major cell survival tools [97,98,99,100,101,102,103,104,105,106], and its persistent inhibition is highly likely to trigger substantial adverse effects. This point is well illustrated by the fate of the transgenic mouse model where Ser51 of eIF2α was substituted by Ala51 (eIF2α Ser51Ala KI mouse) [170]. This replacement is inconsequential in terms of cellular transcription and translation, but it precludes the elicitation of the ISR. Indeed, the active core of the ISR is the phosphorylation of eIF2α at its amino acid residue 51. Although Ser at this position can be phosphorylated, Ala cannot. Therefore, the integrated stress response cannot be elicited in the eIF2α Ser51Ala KI mouse [170]. Thus, this mouse can serve as a model of the effects of systemic suppression of the ISR. Tellingly, these mice survived for only eighteen hours after their birth [170].

## 37. Transient Administration of the ISR Inhibitors Is Feasible but Inefficient

**Figure 14:**
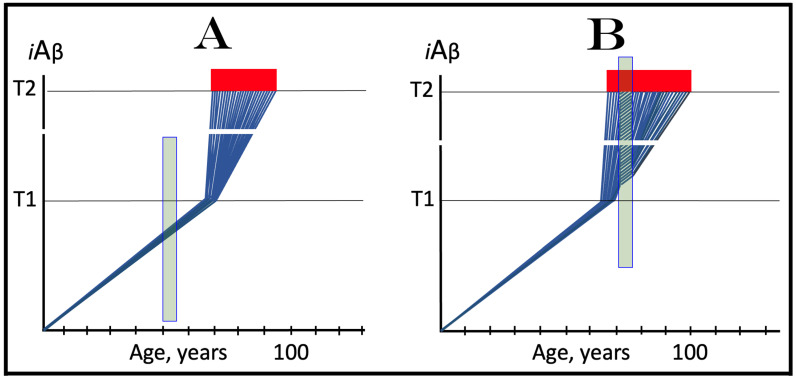
**Transient administration of the ISR inhibitors is feasible but inefficient in both prevention and treatment of conventional AD. *i*Aβ: intraneuronal Aβ**. *Blue lines*: levels of *i*Aβ in individual neurons. ***T*1** *threshold*: cellular concentration of *i*Aβ that triggers the elicitation of the neuronal integrated stress response. ***T*2** *threshold*: levels of *i*Aβ that trigger apoptosis or necroptosis of neuronal cells. *Red box*: apoptotic zone; within this zone the neurons have either undergone apoptosis or are dead. *Green box*: duration of the inhibition of the ISR. (**A**) Transient administration of ISR inhibitors prior to the crossing of the T1 threshold and commencement of AD. At this time, the neuronal ISR has not yet been elicited, and the presence of its inhibitors would have no beneficial effect; the accumulation of AβPP-derived *i*Aβ would continue uninterrupted. (**B**) Transient administration of ISR inhibitors in the treatment of conventional AD. The neuronal ISR has been elicited and the AβPP-independent C100/C99 production pathway rendered operational in all affected neurons. Inhibition of the neuronal ISR interrupts the production of components required for the activity of this pathway and its operation ceases for the duration of the treatment. However, *i*Aβ continues to accumulate at the pre-T1 crossing rate due to the influx of AβPP-derived *i*Aβ. When the transient treatment is concluded, *i*Aβ levels in all affected neurons remain at the over-T1 levels. Consequently, the ISR is re-elicited, the operation of the AβPP-independent C100/C99 generation pathway is restored, and the accumulation of *i*Aβ and progression of AD resumes at the pre-treatment rate; the benefits of the treatment are thus limited to the duration of its administration.

As described in the preceding section, a long-duration administration of ISR inhibitors may prevent the occurrence of AD or slow down the progression of the disease but this strategy is not feasible due to potential severe adverse effects. In contrast, the transient administration of ISR inhibitors was demonstrated to be feasible in experimental mouse models; no significant adverse effects were observed in these studies [161,162,163,164,165,166,167,168,169]. The transient administration of ISR inhibitors will figure prominently in therapeutic strategies discussed below. It is of interest, therefore, to analyze its potential in both the prevention and treatment of AD. The preventive application entails the administration of an ISR inhibitor prior to the crossing of the T1 threshold by AβPP-derived *i*Aβ, as shown in panel A of Figure 14. However, at this time, the neuronal ISR has not yet been elicited, and the presence of its inhibitors would have no beneficial effect; the accumulation of AβPP-derived *i*Aβ would continue uninterrupted. 

Panel B of Figure 14 depicts the effect of the transient administration of ISR inhibitors to the AD patient. At this point, the integrated stress response has been elicited and the AβPP-independent C100/C99 generation pathway activated in all affected neurons. Inhibition of the neuronal ISR at this time would interrupt the production of components required for the activity of this pathway, and its operation would cease for the duration of the treatment. In the absence of the ISR, *i*Aβ would, nevertheless, continue to accumulate at the pre-T1 crossing rate due to the influx of AβPP-derived *i*Aβ. When the transient treatment is concluded, *i*Aβ levels in all affected neurons remain at the over-T1 levels. Consequently, the ISR would be re-elicited, the operation of the AβPP-independent C100/C99 generation pathway restored, and the accumulation of *i*Aβ and progression of AD would resume at the pre-treatment rate; the benefits of the treatment would be limited to the duration of its administration.

## 38. Depleting the Driver of AD: The Removal of *i*Aβ as Therapeutic Strategy

Therapeutic strategies described in the preceding sections target the trigger and sustainer of Alzheimer’s disease, namely the neuronal integrated stress response. The infeasibility of this approach in its long-term application is due not to its conceptual limitations but rather to logistical, operational issues. Another therapeutic strategy, described in the present and following sections, also targets the neuronal integrated stress response but is potentially capable of much more than simply its suppression. Indeed, in the ACH2.0, the elicitation of the neuronal integrated stress response is triggered, via the activation of PKR and/or HRI kinases and phosphorylation of aIF2α, by AβPP-derived *i*Aβ accumulated over the T1 threshold. Therefore, the depletion of *i*Aβ any time prior to the crossing of the T1 threshold would prevent or delay the occurrence of conventional AD. 

The neuronal ISR, when elicited, provides components essential for and thus enables the operation of the AβPP-independent C100/C99 generation pathway. *i*Aβ produced in this pathway accumulates to AD pathology-causing levels and drives the disease. It also propagates the neuronal ISR conditions, and thus perpetuates its own production. This is of special importance in light of our conclusion (see above) that the production of AβPP-derived *i*Aβ is suppressed under the ISR conditions and it can no longer sustain the ISR state. The possibility that C100/C99 generated independently of AβPP is not processed into *i*Aβ (see above) does not change the logic of the present discourse, and in this respect the depletion of *i*Aβ is equivalent to the removal of C99 since the *i*Aβ is the integral part of C99 (further discussed in Section 52 below). The depletion of *i*Aβ in symptomatic AD would remove the driver of the disease. Moreover, if *i*Aβ is depleted to the levels below the ISR-eliciting T1 threshold, the ISR state would be terminated, the production of components enabling the AβPP-independent C100/C99 generation pathway would stop, and the operation of the pathway would cease. 

The accumulation of AβPP-derived *i*Aβ to the over-T1 levels is a very long process, so long, in fact, that in the majority of the human population the lifetime is not sufficient. Even in sporadic AD, it takes over six decades to accumulate AβPP-derived *i*Aβ to the neuronal ISR-eliciting AD-triggering levels. It follows that if the depletion of *i*Aβ is implemented around the mid-life, and is sufficiently deep, i.e., close to the initial baseline, *i*Aβ, produced now solely in the AβPP proteolytic pathway, would not reach the T1 threshold within the lifetime of the treated individual. AD, as well as AACD, would be prevented in this scenario. If the depletion of *i*Aβ were implemented in symptomatic AD patients and its levels were reduced below the T1 threshold, the AβPP-independent C100/C99 generation pathway would be disabled and the progression of AD would stop. If, in this scenario, the *i*Aβ depletion is sufficiently deep, the de novo accumulating AβPP-derived *i*Aβ would not reach the T1 threshold and AD would not resume within the remaining lifetime of the treated AD patient. 

Thus, potentially, a single transient *i*Aβ depletion treatment can confer the life-long protection from conventional AD and from AACD if implemented preventively or arrest the progression of the disease and preclude its recurrence within the remaining lifetime if administered to the symptomatic AD patient (further addressed in Section 41, Section 42, Section 43 and Section 44).

## 39. Modulation of Physiologically Occurring Intra-*i*Aβ Cleavages Can Cause or Prevent AD

The principles of therapeutic strategies for AD formulated in the preceding section are supported by two naturally occurring mutations that modulate physiological cleavages within *i*Aβ (or the Aβ segment of AβPP and C99) and either cause or protect from Alzheimer’s disease. One of these mutations (the Icelandic Aβ mutation) affects the cleavage pattern of BACE1. Another mutation (the Flemish Aβ mutation) modulates intra-*i*Aβ cleavages by BACE2. As follows from their designation, β-site AβPP-cleaving enzymes, BACE1 and BACE2, cleave at the β-site of AβPP (between its amino acid residues 671 and 672). For BACE1, the cleavage at the β-site is its major activity. However, it is also capable of cleaving at the positions Aβ10 (designated β’-site) and Aβ34. BACE2, on the other hand, is also capable of the cleavage at the β-site, but this is its minor activity. The major activity of BACE2 is intra-*i*Aβ cleavages at the positions Aβ19 and Aβ20 [179,180]. The physiological role of the intra-*i*Aβ cleavages by BACE1 and BACE2 is, apparently, the regulation of *i*Aβ levels via the modulation of its influx and efflux.

The Icelandic Aβ mutation increases the rate of the BACE1 cleavage at the β’-site of *i*Aβ (or of Aβ portion of AβPP or C99). This increase in the efficiency of cleavage at the β’-site is only approximately 30% [31]. It is sufficient, nevertheless, to protect carriers of the Icelandic mutation from both AD and AACD. The explanation of this effect, in terms of the ACH2.0, is apparent. The Icelandic Aβ mutation shifts the influx/efflux balance of AβPP-derived *i*Aβ toward the latter. Consequently, the rate of accumulation of AβPP-derived *i*Aβ decreases, and its crossing of both the T^0^ and T1 thresholds is delayed or prevented in the carriers of this mutation. This translates into the delay or prevention of both AD and AACD [4,6].

The Flemish Aβ mutation replaces amino acid residue 21 of Aβ, a position contiguous to the sites of the major cleavage activity of BACE2, namely Aβ19 and Aβ20. This mutation decreases the efficiency of intra-*i*Aβ cleavages at both positions, Aβ19 and Aβ20. It also causes early-onset familial AD with the mean age of commencement at the age 46 and the range of the onset beginning at the age 35 [181,182,183,184,185,186,187]. The interpretation of the effects of the Flemish mutation in terms of the ACH2.0 is no less apparent than that of the effect of the Icelandic mutation. The Flemish mutation shifts the influx/efflux equilibrium of AβPP-derived *i*Aβ toward the former. Consequently, the rate of accumulation of AβPP-derived *i*Aβ increases and the timing of the T1 crossing accelerates. This translates into the accelerated elicitation of the neuronal ISR, activation of the AβPP-independent C100/C99 generation pathway, and early onset of the disease [4,6].

## 40. Activators of BACE1 and BACE2 Are Potential AD Drugs

It follows from the two preceding sections that any agent competent to selectively deplete *i*Aβ would be a possible AD drug. Section 39 lucidly indicates two such potential agents, namely BACE1 and BACE2. They are present and active in neuronal cells and their observed physiological role in both the protection from and causation of AD leaves little doubt regarding their therapeutic potential, provided their activities, or, at least, their intra-*i*Aβ cleaving activities, can be enhanced by suitable activators. Besides their disease-protective (if enhanced) and disease-causing (if suppressed) effects of the intra-*i*Aβ cleaving activities of BACE1 and BACE2, additional findings also indicate their potential therapeutic benefits. For example, the overproduction of BACE1 in neuronal cells of animal models significantly elevated the efficiency of cleavage at the β’-site, increased the ratio of *i*Aβ cleaved at the β’-site to intact *i*Aβ, and reduced extracellular deposition of Aβ [188,189,190,191]. Moreover, when sufficiently overexpressed, BACE1 was shown to cleave *i*Aβ not only at the β’-site but also at the position Aβ34 (the position of cleavage in the physiologically occurring cleavage of *i*Aβ) [192,193,194,195]. This additional cleavage site further increases the therapeutic potential of activated BACE1.

As for BACE2, the observation that its inhibition in experimental models significantly elevated the production of Aβ [180] clearly reflects its potential to substantially reduce the levels of *i*Aβ (or of C99) via its intramolecular cleavages. The concept that the enhancement of the activity of BACE2 is potentially beneficial whereas its reduction is deleterious has been substantiated in experiments with human pluripotent cell-derived brain organoids [196]. In these experiments, BACE2 activity conferred to the organoids protection from the toxicity and neuronal loss caused by Aβ [196]. On the other hand, the deficient activity of BACE2 correlated with AD-characteristic pathology [196]. It could be of interest that the deficient activity of BACE2 also correlated with symptoms of Hirschsprung disease in the brain organoids model [196].

Thus, only a minor (approximately 30%) elevation of the efficiency of BACE1 cleavage at only one position, β’-site, resulted in an extensive protective outcome in the carriers of the Icelandic mutation. Since the intra-*i*Aβ cleavage activity of BACE1, not only at the β’-site but also at the position Aβ34, could potentially be increased by well over 30%, the therapeutic potential of such an enhancement is substantial. The enhancement of the intra-*i*Aβ cleaving activity of BACE2 at the positions Aβ19 and Aβ20 appears even more auspicious since this is the major activity of the enzyme (versus cleavages at the β’-site and the position Aβ34 being the minor activities of BACE1). The enhancement of each, BACE1 and BACE2, appears therapeutically promising, but the concurrent activation of both would synergize and maximize their therapeutic potential due not only to their different target site specificities but also to distinct cellular compartmentalization of both enzymes [197].

## 41. BACE1 and/or BACE2 Activation Therapy Would Be Very Effective in the Prevention of Conventional AD and AACD and in the Treatment of AACD 

The considerations presented in the preceding sections strongly indicate that the activation of BACE1 and/or BACE2, more specifically of the intra-*i*Aβ cleaving capabilities of both enzymes, would be very effective in preventing AD and in both preventing and treating AACD. In all these circumstances (i.e., prevention of AD, prevention and treatment of AACD) activators of BACE1 and/or BACE2 are administered when AβPP-derived *i*Aβ has not yet crossed the T1 threshold, and neither the neuronal ISR has been elicited, or the AβPP-independent C100/C99 generation pathway activated. At this stage, *i*Aβ is derived solely in the AβPP proteolytic pathway and the efficiency of the activation of BACE1 in these circumstances has been proven and that of BACE2 strongly indicated. Therefore, it can be anticipated that the transient administration of BACE1- and/or BACE2-activating drugs would bring the levels of *i*Aβ to a low baseline. The de novo accumulation of *i*Aβ, still derived solely from AβPP proteolysis, would resume at this point and proceed at the pre-treatment rate. The T1 threshold would not be reached and, consequently, AD would not occur within the lifetime of the treated individuals. Precisely the same logic applies also to the prevention of AACD. As long as the BACE1- and/or BACE2-activating treatment is administered prior to the crossing of the T^0^ threshold by AβPP-derived *i*Aβ, its sufficient depletion would ensure that neither would its levels reach the T^0^ threshold nor would AACD occur within the lifetime of treated individuals. 

In this respect, the timing of the drug’s administration is of crucial importance. Fortunately, there is no need to define the values of both T^0^ and T1 thresholds in terms of *i*Aβ concentrations because the knowledge that the mean age of the onset of sporadic AD is at about 65 and that of AACD even higher [115,116] is sufficient. It could be stated, with a considerable degree of certainty, that if the *i*Aβ depletion treatment were successfully (success defined as the depletion of *i*Aβ to a sufficiently low baseline) administered at mid-life (at about 50), neither conventional AD nor AACD would occur within lifetimes of treated individuals.

Moreover, the BACE1 and/or BACE2 activation-mediated depletion of *i*Aβ, administered prior to the crossing of the T1 threshold, would be effective in treating and, actually, curing aging-associated cognitive decline. Indeed, AACD occurs in the range of AβPP-derived *i*Aβ concentrations between the T^0^ and T1 thresholds [4]. In this range, all affected neurons remain viable (no T2 threshold has yet been reached). Therefore, the depletion of AβPP-derived *i*Aβ to levels below the T^0^ threshold would arrest the progression of the AACD condition and enable the neurons to recover and reconnect. If the depletion is adequately deep, the resumed de novo accumulation of AβPP-derived *i*Aβ would be insufficient for it to reach the T^0^ threshold, and neither AACD would recur nor AD would occur within the remaining lifetime of the treated patient.

**Figure 15:**
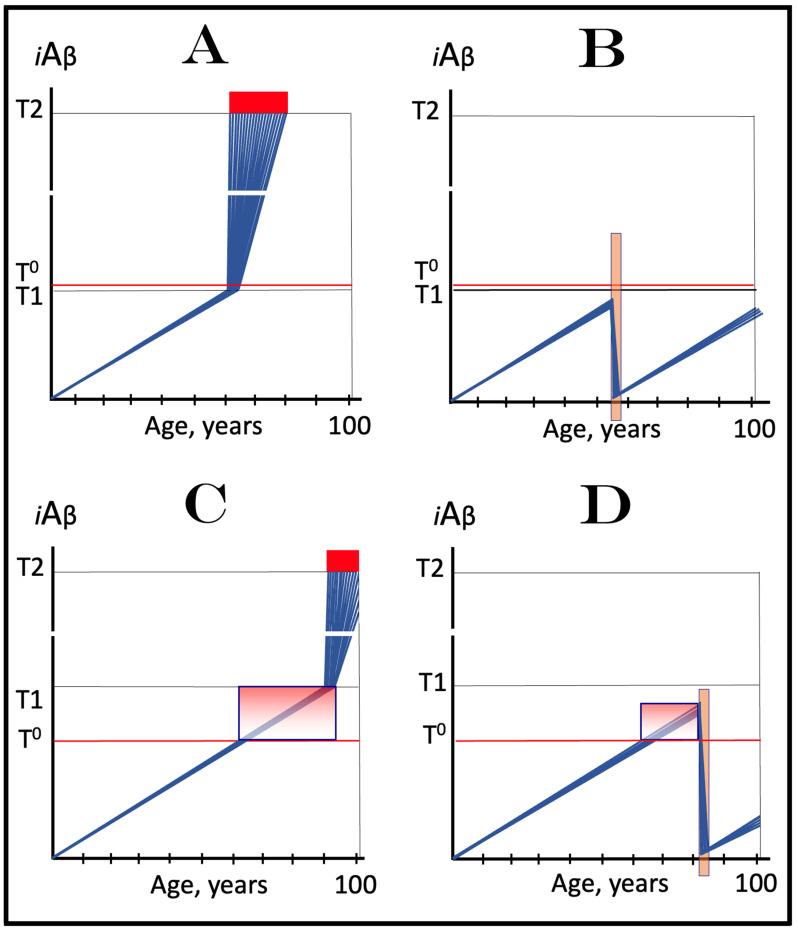
**The depletion of *i*Aβ would be very effective in the prevention of conventional AD and AACD and in the treatment of AACD. *i*Aβ: intraneuronal Aβ**. *Blue lines*: levels of *i*Aβ in individual neurons. ***T*^0^** *threshold*: the level of AβPP-derived *i*Aβ above which the neuronal damage manifesting as AACD commences. ***T*1** *threshold*: cellular concentration of *i*Aβ that triggers the elicitation of the neuronal integrated stress response. ***T*2** *threshold*: levels of *i*Aβ that trigger apoptosis or necroptosis of neuronal cells. *Red box*: apoptotic zone; within this zone, the neurons have either undergone apoptosis or are dead. *Orange boxes*: Duration of the depletion of AβPP-derived *i*Aβ. *Gradient-pink boxes*: The range of AβPP-derived *i*Aβ concentrations between the T^0^ and T1 thresholds (provided T^0^ < T1), a zone of the occurrence of AACD. (**A**,**B**) The extent of the T^0^ is greater than that of the T1 threshold; no AACD can occur. (**A**) Dynamics of the accumulation of *i*Aβ and progression of the disease in the untreated AD patient. AβPP-derived *i*Aβ reaches the T1 threshold and triggers the elicitation of the neuronal ISR and, consequently, the activation of the AβPP-independent C100/C99 generation pathway. *i*Aβ produced independently of AβPP rapidly accumulates and crosses the apoptosis-inducing T2 threshold. (**B**) The transient *i*Aβ depletion treatment is administered prior to the crossing of the T1 threshold and commencement of AD. AβPP-derived *i*Aβ is substantially depleted and its de novo accumulation resumes from a low baseline and proceeds at the pre-treatment rate. It does not reach the T1 threshold and AD does not occur within the lifetime of the treated individual. (**C**,**D**) The extent of the T1 is greater than that of the T^0^ threshold; AD is preceded by AACD. (**C**) Dynamics of the accumulation of *i*Aβ and progression of the disease in the untreated AACD/AD patient. AβPP-derived *i*Aβ reaches the T^0^ threshold and AACD commences. AβPP-derived *i*Aβ continues to accumulate and when it crosses the T1 threshold, the neuronal ISR is elicited, AβPP-independent C100/C99 generation pathway activated, AD commences, and AACD morphs into AD. *i*Aβ produced independently of AβPP rapidly accumulates, crosses into the apoptotic zone, and the disease enters the end stage. (**D**) The transient *i*Aβ depletion treatment is administered when the T^0^ threshold has already been crossed and AACD commenced but prior to the T1 crossing. AβPP-derived *i*Aβ is substantially depleted. The progression of AACD ceases and the condition is cured. AβPP-derived *i*Aβ resumes its de novo accumulation from a low baseline. It does not reach the T^0^ threshold within the remaining lifetime of the treated individual; neither AACD recurs nor AD occurs. Note that if the *i*Aβ depletion treatment were administered prior to the crossing of the T^0^ threshold, both AACD and conventional AD would be prevented.

The considerations of the present section are illustrated in Figure 15. Panels A and B of Figure 15 depict a scenario where the extent of the T^0^ threshold is greater than that of the T1 threshold; no AACD can occur in this scenario [4]. Panel A shows the kinetics of accumulation of *i*Aβ and the progression of AD in the untreated individual. Panel B shows the outcome of the transient *i*Aβ depletion treatment administered prior to the crossing of the T1 threshold. Following the treatment, de novo accumulating AβPP-derived *i*Aβ would not reach the T1 threshold within the lifetime of the individual; no AD would occur. Panels C and D present a scenario where the extent of the T^0^ threshold is smaller than that of the T1 threshold. In this scenario, AD would be preceded by AACD in the untreated individual (panel C). In panel D of Figure 15, the transient *i*Aβ depletion treatment is administered when the T^0^ threshold has already been crossed and AACD commenced but prior to the T1 crossing. Following the treatment, the progression of AACD would cease, the condition would be cured, and de novo accumulating AβPP-derived *i*Aβ would not reach the T^0^ threshold within the remaining lifetime of the treated individual; neither would AACD recur nor AD occur. If, on the other hand, the treatment were administered prior to the crossing of the T^0^ threshold, both conventional AD and AACD would be prevented for the lifetime of the treated individual.

## 42. BACE1 and/or BACE2 Activation Alone Would Be Inefficient in the Treatment of AD Due to the High Rate of Influx of *i*Aβ Produced Independently of AβPP

Conceptually, the therapeutic strategy described in the preceding section, namely the BACE1 and/or BACE2 activation-mediated depletion of *i*Aβ, could be administered to symptomatic AD patients. If *i*Aβ were sufficiently depleted, the neuronal ISR state would no longer be present, the operation of the AβPP-independent C100/C99 generation pathway would cease, and the situation would be akin to that discussed in the preceding section, i.e., *i*Aβ accumulating de novo from a low baseline would be unable to reach the T1 threshold, and AD would nor recur within the remaining lifetime of the treated individual. This scenario, however, is highly unlikely. The problem is that, unlike in the prevention of AD, in symptomatic AD patients, the AβPP-independent C100/C99 generation pathway is operational. As was argued in Section 22, the rate of the influx of *i*Aβ produced independently of AβPP is potentially so high that it cannot be matched, let alone exceeded, by its depletion via the activation of intra-*i*Aβ cleaving capabilities of BACE1 and/or BACE2. The most that plausibly can be expected in these circumstances, even under the long-term treatment, is the curtailment of the rate of accumulation of *i*Aβ produced independently of AβPP but certainly not its depletion. In such a case, the administration of BACE1- and/or BACE2-activating drugs would result only in slowing down the progression of the disease but not in its arrest.

**Figure 16:**
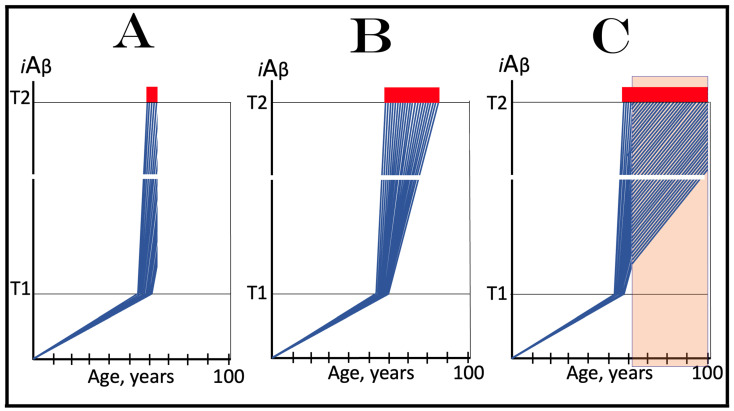
**The depletion of *i*Aβ would be inefficient in the treatment of AD. *i*Aβ: intraneuronal Aβ**. *Blue lines*: levels of *i*Aβ in individual neurons. ***T*1** *threshold*: cellular concentration of *i*Aβ that triggers the elicitation of the neuronal integrated stress response. ***T*2** *threshold*: levels of *i*Aβ that trigger apoptosis or necroptosis of neuronal cells. *Red box*: apoptotic zone; within this zone, the neurons have either undergone apoptosis or are dead. *Orange box*: duration of the depletion of AβPP-derived *i*Aβ. (**A**) Initial state of AβPP-derived *i*Aβ levels in individual neurons. AβPP-derived *i*Aβ has already crossed the T1 threshold in all affected neurons of the AD patient. The neuronal integrated stress response has been elicited, the AβPP-independent C100/C99 generation pathway has been activated, and *i*Aβ produced independently of AβPP has rapidly accumulated. The apoptosis-triggering T2 threshold has been crossed in a fraction of the neurons, but the bulk of the affected neurons remain sub-T2. (**B**) Evolution of the initial state in the untreated AD patient. The AβPP-independent C100/C99 generation pathway remains operational, the accumulation of *i*Aβ produced independently of AβPP continues uninterrupted, and more neurons cross the T2 threshold, thus leading to the end stage of the disease. (**C**) Evolution of the initial state in the presence of BACE1 and/or BACE2 activators. The rate of the influx of *i*Aβ produced in the AβPP-independent pathway outbalances that of the removal of *i*Aβ by intra-*i*Aβ cleaving activities of activated BACE1 and/or BACE2. Its accumulation as well as the progression of the disease persists, although at a decreased rate, for the duration of the treatment.

The above scenario is illustrated in Figure 16. Panel A presents the initial state of *i*Aβ levels in the affected neurons of the AD patient at the time of the administration of BACE1- and/or BACE2-activating drugs. At this stage, they already crossed the T1 threshold and triggered the elicitation of the neuronal ISR and the activation of the AβPP-independent C100/C99 generation pathway. A fraction of neurons has reached the T2 threshold and undergone apoptosis but the bulk of neuronal cells remain sub-T2. Panel B of Figure 16 depicts the evolution of the initial state in the absence of drugs. The disease progresses unimpeded. *i*Aβ rapidly accumulates, additional neurons reach the T2 threshold, the neuronal loss persists, and the disease enters the end stage. Panel C of Figure 16 presents the evolution of the initial state in the presence of BACE1- and/or BACE2-activating drugs (orange box). The rate of the efflux of *i*Aβ increases but that of its influx remains unchanged. Consequently, the rate of its accumulation is reduced but not reversed. The disease persists but the rate of its progression is reduced. 

It should be emphasized that the considerations of the present section are based on the presumption that the production of BACE enzymes is not suppressed under the neuronal integrated stress response conditions. It is possible, even plausible, that this is not the case. Scenarios where the production of both BACE1 and BACE2 is suppressed in AD-affected neurons under the neuronal ISR conditions are considered in the following two sections.

## 43. BACE1 and/or BACE2 Activation in the Treatment of AD Could Be Unfeasible Due to the Suppression of Their Production by the Neuronal ISR

As described in Section 41, the activation of intra-*i*Aβ cleaving capabilities of BACE1 and/or BACE2 is expected to be very efficient in the prevention of AD and AACD and in the treatment of AACD i.e., in the circumstances where AβPP-derived *i*Aβ has not yet reached the T1 threshold and the neuronal integrated stress response has not yet been elicited. The preceding section elaborates why this is not the case in the treatment of symptomatic AD: the rate of the accumulation of *i*Aβ produced independently of AβPP can be reduced but not reversed. There is, however, another reason why the activation of intra-*i*Aβ cleaving capabilities of BACE1 and/or BACE2 may be not feasible as a therapeutic strategy in the treatment of AD. To put it simply: neither BACE1 nor BACE2 may be present (or present at the meaningful levels) in the AD-affected neurons under the neuronal ISR conditions, a possibility that was brought up in Section 34 and Section 35. Under the integrated stress response conditions, the global cellular protein synthesis, i.e., translation of the decisive bulk of cellular proteins, is severely suppressed. There is no reason to presume that components of the AβPP proteolytic pathway, including AβPP, BACE enzymes, and constituents of the γ secretase complex are not included in the suppressed majority. To the opposite, the considerations presented above indicate that they are. This conclusion is consistent with the inability of AβPP-derived *i*Aβ to trigger the full symptomatic spectrum of AD in transgenic animal models as well as with the observed significant reduction in BACE levels in CSF of the advanced AD patients [198,199,200,201] and substantial elevation of C99 levels in AD, which correlates with the severity of the disease [177,178]. 

To summarize, there are two obstacles on the path to enacting the activation of BACE1 and/or BACE2 as a therapeutic strategy for the treatment of symptomatic AD: the overwhelming efficiency of the AβPP-independent C100/C99 generation pathway and the availability (or rather lack thereof) of both BACE enzymes. Fortunately, as described in the following section, both obstacles can be overridden by the inclusion of the inhibitors of the integrated stress response in the composite therapeutic strategy for the treatment of symptomatic AD.

## 44. Composite Therapeutic Strategy for Symptomatic AD: The Dual Duty of the Neuronal ISR Inhibitors Administered Concurrently with the Activators of BACE Enzymes—(1) Enabling the Continuous Production of BACE1 and BACE2 and (2) Ceasing the Influx of *i*Aβ Produced in the AβPP-Independent Pathway 

Both problems precluding the utilization of the activators of intra-*i*Aβ cleaving capabilities of BACE1 and/or BACE2 in the treatment of symptomatic AD have a common origin, namely the neuronal integrated stress response. Indeed, under the neuronal ISR conditions, elicited in conventional AD by AβPP-derived *i*Aβ at the over-T1 levels, the production of BACE1 and BACE2 is presumably suppressed, hence, problem one. The same neuronal ISR conditions induce the production of components essential for the operation of the AβPP-independent C100/C99 generation pathway. When this pathway is operational, it produces *i*Aβ (or unprocessed C99, see Section 52 below) at an extraordinary rate, hence problem two. Thus, “problem one” leads to “problem two”, but these problems are intertwined also in reverse: *i*Aβ (or C99) produced independently of AβPP sustains the activated state of the eIF2α kinases and thus propagates the neuronal ISR conditions and, consequently, perpetuates its own production. 

Breaking this “vicious” feedback loop would potentially abolish both problems. This is precisely what the composite therapeutic strategy for symptomatic AD is designed to do. The first element of this strategy is the inhibition of the neuronal integrated stress response. As we established above, the persistent systemic inhibition of the ISR is not feasible. On the other hand, the transient inhibition of the ISR is feasible, as exemplified by its successful application in the abrogation, without noticeable adverse effects, of the neuronal ISR-triggered cognitive impairment in transgenic animal model overexpressing (by AβPP proteolysis) Aβ [161,163]. With the suppression of the neuronal ISR, the production of BACE1 and BACE2, and thus their availability, is restored (solution of the “problem one”). Moreover, with the ISR inhibited, the supply of components essential for the operation of the AβPP-independent C100/C99 generation pathway ceases, the pathway is rendered inoperative, and the influx of its *i*Aβ (or C99) product is interrupted and abolished (solution of the “problem two”). 

Concurrently with the inhibition of the neuronal ISR, the second element of the composite therapeutic strategy for symptomatic AD is engaged: the activation of intra-*i*Aβ cleaving capabilities of BACE1 and/or BACE2. With no influx of *i*Aβ (or C99) produced independently of AβPP, the efficiency of activated BACE1 and/or BACE2 would be comparable to that exhibited prior to the crossing of the T1 threshold. Consequently, levels of *i*Aβ would be relatively rapidly depleted to a low baseline. Following the withdrawal of both, ISR inhibitors and BACE activators, the neuronal ISR is not re-elicited and the AβPP-independent C100/C99 generation pathway remains inoperative. In this composite strategy, the duration of BACE activation is defined as the time period needed for the sufficient depletion of *i*Aβ. The duration of the ISR inhibition (a potential bottleneck because the overexposure to ISR inhibitors may trigger adverse effects) is less restrictive: ISR inhibitors can be withdrawn when *i*Aβ levels are below the T1 threshold (ensuring that the ISR cannot be re-elicited and the AβPP-independent C100/C99 generation pathway reactivated). Upon conclusion of the treatment, the de novo accumulation of AβPP-derived *i*Aβ resumes at the pre-T1 crossing rate from a low baseline. Neither would it reach the T1 threshold, nor would AD resume within the remaining lifetime of the treated patient.

**Figure 17:**
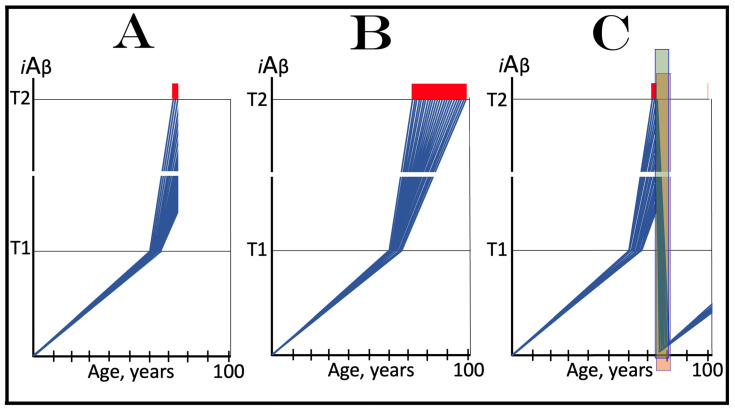
**Composite therapeutic strategy for conventional symptomatic AD: concurrent transient administration of ISR inhibitors and BACE1 and/or BACE2 activators**. *i*Aβ: intraneuronal Aβ. *Blue lines*: levels of *i*Aβ in individual neurons. *T*1 *threshold*: cellular concentration of *i*Aβ that triggers the elicitation of the neuronal integrated stress response. *T*2 *threshold*: levels of *i*Aβ that trigger apoptosis or necroptosis of neuronal cells. *Red box*: apoptotic zone; within this zone, the neurons have either undergone apoptosis or are dead. *Green box*: duration of the administration of ISR inhibitors. *Orange box*: duration of the depletion of AβPP-derived *i*Aβ: (**A**) Initial state of AβPP-derived *i*Aβ levels in individual neurons. AβPP-derived *i*Aβ has already crossed the T1 threshold in all affected neurons of the AD patient. The neuronal integrated stress response is elicited, the AβPP-independent C100/C99 generation pathway is activated, and *i*Aβ produced independently of AβPP rapidly accumulates. The apoptosis-triggering T2 threshold crosses in a fraction of the neurons, but the bulk of the affected neurons remain sub-T2. (**B**) Evolution of the initial state in the untreated individual. The AβPP-independent C100/C99 generation pathway remains operational, the accumulation of *i*Aβ produced independently of AβPP continues uninterrupted, and more neurons cross the T2 threshold, leading to the end stage of the disease. *i*Aβ produced independently of AβPP accumulates, reaches the T2 threshold, and the disease enters the end stage. (**C**) Evolution of the initial state in the treated AD patient. The transient ISR inhibition and BACE activation are implemented concurrently. The former enables the production of BACE1 and BACE2 and thus ensures their availability, and disables the AβPP-independent C100/C99 generation pathway, thus abolishing the influx of *i*Aβ produced independently of AβPP and ensuring its efficient depletion by the latter. Following the treatment, the neuronal ISR state is reversed to normal, the AβPP-independent C100/C99 generation pathway is left inoperative, and AβPP-derived *i*Aβ resumes its accumulation de novo from a low baseline. It does not reach the T1 threshold, and AD does not recur within the remaining lifetime of the treated patient.

To summarize, in the composite therapeutic strategy for symptomatic AD, the transient inhibition of the neuronal integrated stress response carries out a double duty: It enables the production of BACE1 and BACE2 and thus ensures their availability, and disables the AβPP-independent C100/C99 generation pathway, thus abolishing the influx of *i*Aβ (or C99) produced independently of AβPP and ensuring its efficient depletion. This strategy is illustrated in Figure 17. Panel A depicts the initial state of the levels of *i*Aβ in individual neurons of the symptomatic AD patient. Panel B shows the evolution of the initial state in the untreated patient: the disease progresses unimpeded and enters its end stage. Panel C of Figure 17 presents the implementation of the composite therapeutic strategy. Inhibitors of the ISR (green box) and activators of BACE1 and/or BACE2 (orange box) are administered concurrently and transiently. Following the treatment, the neuronal ISR state is reversed to normal, the AβPP-independent C100/C99 generation pathway is left inoperative, and AβPP-derived *i*Aβ resumes its accumulation de novo from a low baseline. It does not reach the T1 threshold, and AD does not recur within the remaining lifetime of the treated patient. 

## 45. Unconventional Alzheimer’s Disease: Sustained Activation of the Neuronal ISR by Stressors Other than AβPP-Derived *i*Aβ 

ACH2.0 posits that, conventionally, AD is triggered by the elicitation of the neuronal integrated stress response. The neuronal ISR provides components essential for and enables and sustains the operation of the AβPP-independent C100/C99 generation pathway, which drives AD. In other words, elicit the neuronal ISR in a sufficient fraction of the neuronal population and AD will inevitably ensue [8,9]. It follows that the sustainable elicitation of the neuronal ISR by ANY means would inevitably result in AD. Accordingly, the cases of Alzheimer’s disease can be separated into two major categories: conventional and unconventional [8,9]. In conventional AD, the neuronal ISR is elicited, and, consequently, the disease is triggered by AβPP-derived *i*Aβ accumulated over the T1 threshold via the activation of the PKR and/or HRI kinases and the resulting phosphorylation of eIF2α. In unconventional AD, stressors other than AβPP-derived *i*Aβ elicit the neuronal integrated stress response and trigger the disease. In fact, any stressor capable of the persistent activation of any one of the four eIF2α kinases can trigger unconventional AD. Such stressors are numerous and so are conditions capable of triggering unconventional AD. These conditions could be of mechanical origin, e.g., traumatic brain injury (TBI) [202,203,204] and chronic traumatic encephalopathy (CTE) [205]. They also encompass both viral and bacterial infections [206,207,208,209,210,211,212,213,214]. A person persistently infected with viral encephalitis, for instance, has more than thirty times greater probability of developing AD than an uninfected individual [206,207,208,209,210]. Similarly, various chronic bacterial infections increase the probability of the occurrence of AD by more than ten-fold [211,212,213,214]. Conditions strongly associated with AD also include systemic inflammation, neuroinflammation and even localized inflammations such as osteoarthritis and rheumatoid arthritis [215,216,217,218,219,220,221,222,223,224,225,226]. Cases of dementia linked to these conditions are usually clustered into two groups: AD-related dementia (ADRD) and AD-like dementia (ADLD). In terms of the ACH2.0, these groups are, unquestionably, constituents of the unconventional AD category [8,9].

**Figure 18:**
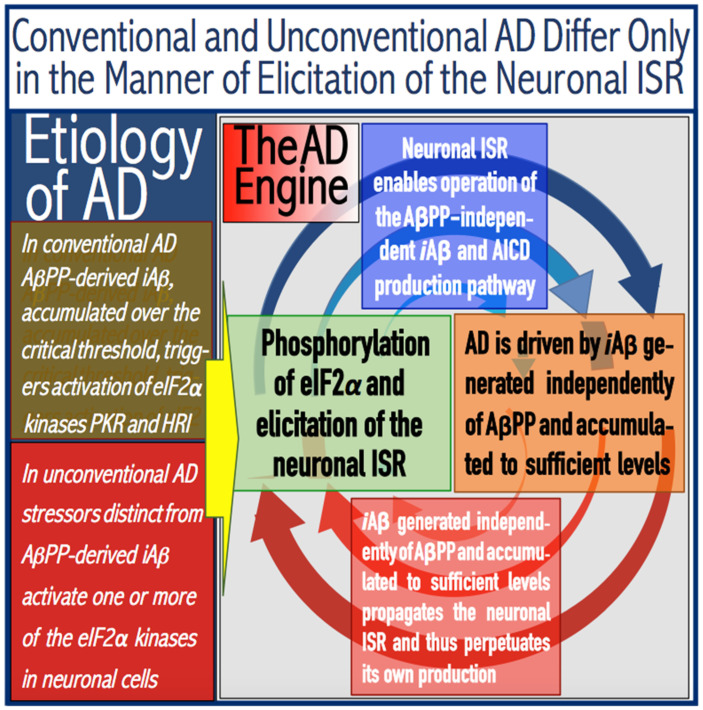
**Conventional and unconventional AD differ only in the manner of the elicitation of the neuronal ISR.** *i*Aβ: intraneuronal Aβ. *AβPP*: Aβ protein precursor. *eIF2α:* eukaryotic translation initiation factor 2*α*. *PKR* and *HRI:* kinases capable of phosphorylating eIF2α when activated. *TNFα*: tumor necrosis factor α, potentially capable of activating PKR. *PACT*: PKR activator. *OMA1:* mitochondrial distress-activated mitochondrial protease. *DELE1:* substrate of OMA1; its cleavage leads to the activation of HRI. *ISR:* neuronal integrated stress response; it is elicited by phosphorylation of eIF2α and enables the production of components essential for the operation of the AβPP-independent *i*Aβ generation pathway. *AICD:* AβPP intracellular domain; it results from the processing of C99. *AD Engine* (denoted by the arched blue and red arrows): repeated feedback cycles of the propagation of the ISR state by *i*Aβ generated independently of AβPP and the resulting stimulation of its own production. In conventional AD, the elicitation of the neuronal ISR is triggered via activation of PKR and/or HRI kinases and phosphorylation of eIF2α, by AβPP-derived *i*Aβ accumulated over the T1 threshold (designated “conventional stressor”). In unconventional AD, the elicitation of the neuronal ISR is triggered via activation of one or more eIF2α kinases, by stressors other than AβPP-derived *i*Aβ (designated “unconventional” stressors). In both forms of AD, the neuronal ISR enables, via the supply of essential components, the operation of the AβPP-independentC100/C99 generation pathway. The resulting *i*Aβ, produced independently of AβPP, drives AD pathology, propagates the neuronal ISR, and perpetuates its own production. In contrast to conventional AD, where the AβPP-independent C100/C99 generation pathway is self-sustainable from the instance of its activation (because *i*Aβ levels are already above the T1 threshold), in unconventional AD, the AβPP-independent C100/C99 generation pathway is activated at the levels of AβPP-derived *i*Aβ below the T1 threshold and becomes self-sustainable only when its *i*Aβ product crosses the latter. In this scenario, the accumulation of *i*Aβ produced in the AβPP-independent pathway is accompanied by a corresponding accumulation of AICD. AICD was demonstrated to be capable of interfering with various AD components but its potential contribution to the disease remains to be elucidated [237,238,239,240,241,242,243,244,245,246,247,248,249,250,251,252].

Specific signal transmission pathways utilized by the conditions described above to elicit the neuronal ISR remain, in most cases, to be elicited. On the other hand, they contain, apparently, two common elements. One is a compromised blood–brain barrier (BBB) [227] that enables the stressors to penetrate the brain and, subsequently, the neurons. Another common element is the diminution of cerebral blood flow (CBE) [228,229,230,231,232,233,234]. One of the ways CBE elicits the neuronal ISR is by triggering neuronal mitochondrial dysfunction [235,236] and, consequently, activating the HRI kinase, thus phosphorylating eIF2α and eliciting the neuronal integrated stress response. In addition, it was suggested that conventional AD can potentially morph into unconventional AD by depositing Aβ around cerebral blood vessels and thus compromising the BBB and diminishing the CBE [8]. Such a possibility may have significant practical implications since therapeutic strategies for unconventional AD differ from those for conventional AD (see below). It is important to emphasize that conventional and unconventional forms of AD differ only in the manner of their initiation, i.e., the elicitation of the neuronal integrated stress response; from this instance forward, both forms of the disease are mechanistically identical, as illustrated in Figure 18.

## 46. Dynamics of *i*Aβ in Unconventional AD Differs from That in the Conventional Form of the Disease

### 46.1. Effect of the Long-Duration Presence of Unconventional Stressors Capable of the Elicitation of the Neuronal ISR 

One principal difference between the dynamics of *i*Aβ in unconventional AD versus that in the conventional form of the disease is that, in the latter, the phase of slow accumulation of AβPP-derived *i*Aβ changes into the phase of fast accumulation of *i*Aβ produced independently of AβPP only with the crossing of the T1 threshold; only then can the neuronal ISR conventionally be elicited and the AβPP-independent C100/C99 generation pathway be activated. In contrast, in the former, the change from the phase of slow *i*Aβ accumulation to that of fast *i*Aβ accumulation occurs always below the T1 threshold. It depends only on the occurrence of unconventional stressors (i.e., stressors distinct from AβPP-derived *i*Aβ) and can potentially occur any time prior to the T1 crossing (if unconventional stressors were to occur only after the T1 crossing, the latter would conventionally activate the AβPP-independent C100/C99 generation pathway and, following this, the occurrence of unconventional stressors would be redundant). The occurrence of unconventional stressors triggers the elicitation of the neuronal ISR, which, in turn, enables the activation of the AβPP-independent C100/C99 production pathway. 

**Figure 19:**
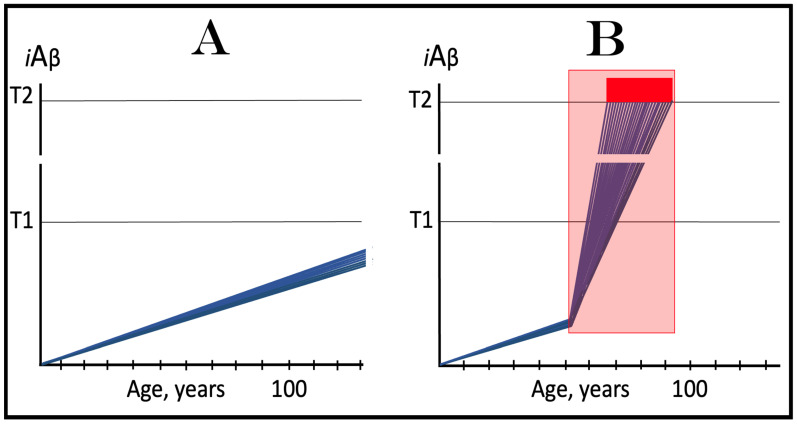
**Dynamics of iAb in unconventional AD: Effect of the long-duration occurrence of unconventional stressors capable of the elicitation of the neuronal ISR**. *i*Aβ: intraneuronal Aβ. *Blue lines*: levels of *i*Aβ in individual neurons. *T*1 *threshold*: cellular concentration of *i*Aβ that triggers the elicitation of the neuronal integrated stress response. *T*2 *threshold*: levels of *i*Aβ that trigger apoptosis or necroptosis of neuronal cells. *Red box*: apoptotic zone; within this zone, the neurons have either undergone apoptosis or are dead. *Pink box*: duration of the presence of unconventional stressors capable of triggering the elicitation of the neuronal ISR: (**A**) Dynamics of the accumulation of *i*Aβ in a healthy person. *i*Aβ is produced solely by the proteolysis of AβPP; its levels do not reach the T1 threshold and the unconventional stressors do not occur within the lifetime of the individual. (**B**) Unconventional stressors occur when levels of AβPP-derived *i*Aβ are below the T1 threshold and persist for the remaining lifetime. The neuronal ISR is unconventionally elicited, and the AβPP-independent C100/C99 production pathway is activated. When *i*Aβ produced in this pathway crosses the T1 threshold, the pathway becomes self-sustainable, and AD commences and progresses to its end stage.

Another principal difference between the dynamics of *i*Aβ in unconventional AD versus that in the conventional form of the disease is that, in the former, the newly activated AβPP-independent C100/C99 generation pathway is initially not self-sustainable, i.e., its product (presumably *i*Aβ but potentially C99) is insufficient to sustain its operation. Indeed, if unconventional stressors were withdrawn at this point, the operation of the AβPP-independent pathway would cease because, with *i*Aβ levels below the T1 threshold, the ISR conditions would reverse to normal. Only with the levels of *i*Aβ at or above the T1 threshold would the AβPP-independent C100/C99 production pathway become self-sustainable. The dynamics of *i*Aβ in unconventional AD is illustrated in Figure 19. For purposes of comparison, panel A in Figure 19 shows the dynamics of *i*Aβ in a healthy individual. *i*Aβ is derived solely from AβPP and its dynamics is single-phased; its levels do not reach the T1 threshold, and the unconventional stressors do not occur within the lifetime of the individual. As shown in panel B of Figure 19, unconventional stressors occur when levels of AβPP-derived *i*Aβ are below the T1 threshold and persist for the remaining lifetime (pink box). The neuronal ISR is unconventionally elicited, and the AβPP-independent C100/C99 production pathway is activated. When *i*Aβ produced in this pathway crosses the T1 threshold, the pathway becomes self-sustainable, and AD commences and progresses to its end stage.

### 46.2. Effect of the Transient Presence of Unconventional Stressors Capable of Eliciting the Neuronal ISR

**Figure 20:**
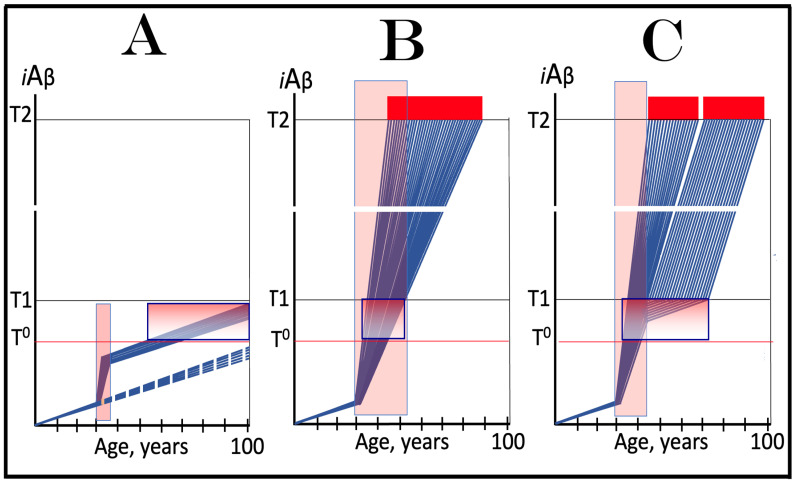
**Dynamics of *i*Aβ in unconventional AD: Effect of the transient occurrence of unconventional stressors capable of the elicitation of the neuronal ISR**. *i*Aβ: intraneuronal Aβ. *Blue lines*: levels of *i*Aβ in individual neurons. *Broken lines*: projected accumulation of AβPP-derived *i*Aβ in the absence of unconventional stressors. *T*^0^ *threshold*: the level of AβPP-derived *i*Aβ above which the neuronal damage manifesting as AACD commences. *T*1 *threshold*: cellular concentration of *i*Aβ that triggers the elicitation of the neuronal integrated stress response. *T*2 *threshold*: levels of *i*Aβ that trigger apoptosis or necroptosis of neuronal cells. *Red box*: apoptotic zone; within this zone, the neurons have either undergone apoptosis or are dead. *Pink box*: duration of the presence of unconventional stressors capable of triggering the elicitation of the neuronal ISR. *Gradient-pink boxes*: the range of AβPP-derived *i*Aβ concentrations between the T^0^ and T1 thresholds (provided T^0^ < T1), a zone of the occurrence of AACD. (**A**) The unconventionally activated AβPP-independent C100/C99 generation pathway is operational for only a short duration, insufficient for the T1 crossing by *i*Aβ. When unconventional stressors are withdrawn, *i*Aβ, produced solely by AβPP proteolysis, continues to accumulate at a slow rate. It does not reach the T1 threshold, and no AD occurs within the lifespan of the individual. It does, however, cross the T^0^ threshold; AACD commences and persists for the remaining lifetime of the individual. (**B**) The unconventionally activated AβPP-independent C100/C99 production pathway operates sufficiently long for its *i*Aβ product to cross both the T^0^ and T1 thresholds. Upon the T^0^ crossing AACD commences, and when the T1 is crossed, the AβPP-independent C100/C99 production pathway is rendered self-sustainable, and AACD morphs into AD. (**C**) The unconventionally activated AβPP-independent C100/C99 production pathway operates long enough for its *i*Aβ product to cross the T^0^ and T1 thresholds in only a portion of the affected neurons. Upon the T^0^ crossing, AACD commences and persists until the T1 crossing. When unconventional stressors are withdrawn, the operation of the AβPP-independent C100/C99 generation pathway in neurons that cross the T1 threshold continues uninterrupted and self-sustainably. In the rest of the affected neurons, the operation of the unconventionally activated AβPP-independent C100/C99 production pathway ceases. The accumulation of AβPP-derived *i*Aβ in these neurons continues at a slow rate; when it crosses the T1 threshold, the AβPP-independent C100/C99 generation pathway is conventionally activated, and the progression of AD pathology commences.

As discussed above, initially, until the levels of *i*Aβ cross the T1 threshold, the unconventionally activated AβPP-independent C100/C99 generation pathway is not self-sustainable. Therefore, the effects of the transient presence of unconventional stressors capable of eliciting the neuronal ISR (pink boxes in figures below) will largely depend on whether or not *i*Aβ has crossed the T1 threshold at the time of the withdrawal of stressors. Three possible outcomes of the transient presence of unconventional stressors are illustrated in Figure 20. In panel A of Figure 20, unconventional stressors are present, and the unconventionally activated AβPP-independent C100/C99 generation pathway is operational for only a short duration. Because the phase of the fast *i*Aβ accumulation is short, the extent of its accrual is also small. When unconventional stressors are withdrawn, *i*Aβ, produced now solely by AβPP proteolysis, continues to accumulate at a slow rate. It does not reach the T1 threshold, and no AD occurs within the lifespan of the individual. The only consequence in this scenario is the possible facilitation of the occurrence of AACD (see below). In panel B of Figure 20, unconventionally activated AβPP-independent C100/C99 production pathway operates sufficiently long for its *i*Aβ product to cross the T1 threshold. Following the T1 crossing, the AβPP-independent C100/C99 production pathway is rendered self-sustainable. The withdrawal of unconventional stressors at this point does not interrupt its continuous operation. If unconventional stressors are withdrawn when only a portion of the affected neurons cross the T1 threshold, as shown in panel C of Figure 20, the operation of the AβPP-independent C100/C99 generation pathway in these neurons continues uninterrupted because it gains self-sustainability. In the rest of the affected neurons, the operation of the AβPP-independent C100/C99 production pathway ceases. The accumulation of AβPP-derived *i*Aβ in these neurons would continue at a slow rate; when it crosses the T1 threshold, the AβPP-independent C100/C99 generation pathway is conventionally activated, and the progression of AD pathology commences.

### 46.3. Unconventional Elicitation of the Neuronal ISR and AACD: Unconventional AACD?

Figure 20 also addresses an interesting question: what happens when *i*Aβ produced in the unconventionally activated AβPP-independent pathway crosses the T^0^ threshold? In conventional cases described above (Section 14) and elsewhere [4], AACD was defined as the manifestation of the neuronal damage caused by AβPP-derived *i*Aβ at its range of cellular concentrations between the T^0^ and T1 thresholds. In conventional cases, there is no *i*Aβ produced independently of AβPP below the T1 threshold, but in unconventional cases, there is. Indeed, as described above, the unconventional elicitation of the neuronal ISR and, consequently, the unconventional activation of the AβPP-independent *i*Aβ production pathway (provided γ-secretase is operational and C100/C99 is processed into *i*Aβ) occur always below the T1 threshold. In such a case, it can be safely presumed that in the range of concentrations between the T^0^ and T1 threshold, iAβ generated independently of AβPP elicits the same cellular response as AβPP-derived *i*Aβ. In this respect, Figure 20 presents three possible scenarios. In panel A, without an unconventional event, AβPP-derived *i*Aβ would not cross the T^0^ threshold within the lifetime of the individual (broken lines). Following the transient activity of the AβPP-independent pathway, the accumulation of AβPP-derived *i*Aβ resumes from the elevated baseline, it crosses the T^0^ (but not T1) threshold and triggers the AACD condition (gradient-pink box), which persists for the remaining lifetime of the individual.

In panel B of Figure 20, unconventionally produced *i*Aβ reaches the T^0^ threshold and triggers the AACD condition, which morphs into AD when the T1 threshold is crossed. In panel C of Figure 20, the AACD condition is triggered as the result of the crossing of the T^0^ threshold by unconventionally produced *i*Aβ and persists until AβPP-derived *i*Aβ, accumulating from an elevated baseline following the termination of the transient unconventional event, completes the T1 crossing.

In the scenarios considered in the present subsection, the “AACD” condition may potentially occur at quite a young age. In such a case, it would no longer be “aging-associated cognitive decline” and should be designated differently. For convenience, we refer to it as “unconventional AACD”; it deserves, of course, a more suitable name, which eventually should be allocated to it.

### 46.4. Effect of the Recurrent Transient Occurrences of Unconventional Stressors Capable of Eliciting the Neuronal ISR 

**Figure 21:**
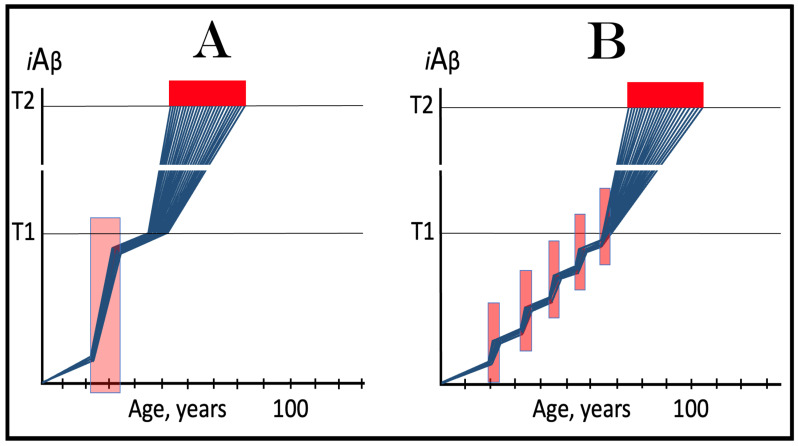
**Dynamics of iAβ in unconventional AD: Effect of the recurrent transient occurrence of unconventional stressors capable of the elicitation of the neuronal ISR**. *i*Aβ: intraneuronal Aβ. *Blue lines*: levels of *i*Aβ in individual neurons. *T*1 *threshold*: cellular concentration of *i*Aβ that triggers the elicitation of the neuronal integrated stress response. *T*2 *threshold*: levels of *i*Aβ that trigger apoptosis or necroptosis of neuronal cells. *Red box*: apoptotic zone; within this zone, the neurons have either undergone apoptosis or are dead. *Pink box*: duration of the presence of unconventional stressors capable of triggering the elicitation of the neuronal ISR: (**A**) Transient operation of the unconventionally activated AβPP-independent C100/C99 generation pathway accelerates the occurrence of conventional AD. The duration of the operation of the unconventionally activated AβPP-independent C100/C99 production pathway is such that the resulting accumulation of its *i*Aβ product is substantial, but its levels are still below the T1 threshold. When unconventional stressors are withdrawn, the operation of the AβPP-independent pathway ceases, but AβPP-derived *i*Aβ continues to accumulate at a slow rate but from a significantly elevated baseline. When it crosses the T1 threshold, the neuronal ISR is conventionally re-elicited, the AβPP-independent C100/C99 generation pathway is reactivated, and AD commences. (**B**) Effect of the recurrent transient occurrence of unconventional stressors capable of the elicitation of the neuronal ISR. The unconventional elicitation of the neuronal integrated stress response and the operation of the unconventionally activated AβPP-independent C100/C99 production pathway occurs for a short duration but recurrently. *i*Aβ accumulates in a stepwise fashion with incremental rounds of fast accumulation, resulting from the transient operation of unconventionally activated AβPP-independent C100/C99 production pathway, interspersed by the accumulation of AβPP-derived *i*Aβ occurring at a slow rate but from repeatedly elevated baselines. This accelerates the T1 crossing, and when it occurs, the self-sustainable AβPP-independent C100/C99 generation pathway is activated, and AD commences.

Two additional possible scenarios associated with the transient presence of unconventional stressors capable of eliciting the neuronal ISR are illustrated in Figure 21. In panel A of Figure 21, the duration of the operation of the unconventionally activated AβPP-independent C100/C99 production pathway is such that the resulting accumulation of its *i*Aβ product is substantial but its levels are still below the T1 threshold. When unconventional stressors are withdrawn, and the operation of the AβPP-independent pathway ceases, AβPP-derived *i*Aβ continues to accumulate at a slow rate but from a significantly elevated baseline. When it crosses the T1 threshold, the neuronal ISR is conventionally re-elicited, the AβPP-independent C100/C99 generation pathway is reactivated, and AD commences and progresses uninterrupted. In this scenario, the transient unconventional elicitation of the neuronal ISR greatly accelerates the conventional activation of the AβPP-independent C100/C99 production pathway and the occurrence of AD. This explains how a single traumatic brain injury results in Alzheimer’s disease a decade or more after the TBI event.

Panel B of Figure 21 simulates the outcome of CBE, chronic traumatic encephalopathy. In this panel, the unconventional elicitation of the neuronal integrated stress response and the operation of the unconventionally activated AβPP-independent C100/C99 production pathway occurs for a short duration but recurrently (e.g., repeatedly occurring brain injury). Consequently, *i*Aβ accumulates in a stepwise fashion with incremental rounds (each following a traumatic event) of fast accumulation (resulting from the transient operation of unconventionally activated AβPP-independent C100/C99 production pathway), interspersed by the accumulation of AβPP-derived *i*Aβ occurring at a slow rate but from repeatedly elevated baselines. This accelerates the T1 crossing, and when it occurs, the self-sustainable AβPP-independent C100/C99 generation pathway is activated, and AD commences.

## 47. Effect of Composite Therapeutic Strategy in the Prevention of Unconventional AD: Transient Activation of BACE1 and/or BACE2 Would Be Effective Only in the Presence of the ISR Inhibitors

The present and the next two sections consider therapeutic strategies for unconventional AD. The effect of inhibitors of the neuronal integrated stress response in unconventional AD is principally the same as in the conventional form of the disease, namely the long-duration treatment is beneficial but not feasible due to the anticipated adverse effects, and the transient administration of ISR inhibitors is feasible but of little benefit. As for the utilization of BACE1 and/or BACE2 activators on their own, even if BACE enzymes are not suppressed under the neuronal ISR conditions, the effect of their activation would be no greater than that shown in panel C of Figure 16 above. In its preventive application, this strategy would only delay but not prevent the disease (because the AβPP-independent C100/C99 production pathway is activated at levels of *i*Aβ below the T1 threshold), and in the treatment of AD, the progression of the disease would be slowed down but not arrested. And, if BACE enzymes are suppressed under the neuronal ISR conditions, their activation on its own is irrelevant as a therapeutic strategy.

In the present and following sections, we assume that the production of both BACE1 and BACE2 is suppressed under the neuronal integrated stress response conditions. Therefore, only composite therapeutic strategies where BACE activation is implemented concurrently with the ISR inhibition are considered henceforth. Moreover, for purposes of simplification of the present discussion, we assume that once unconventional stressors capable of triggering the elicitation of the neuronal ISR occur, they persist for the remaining lifetime.

**Figure 22:**
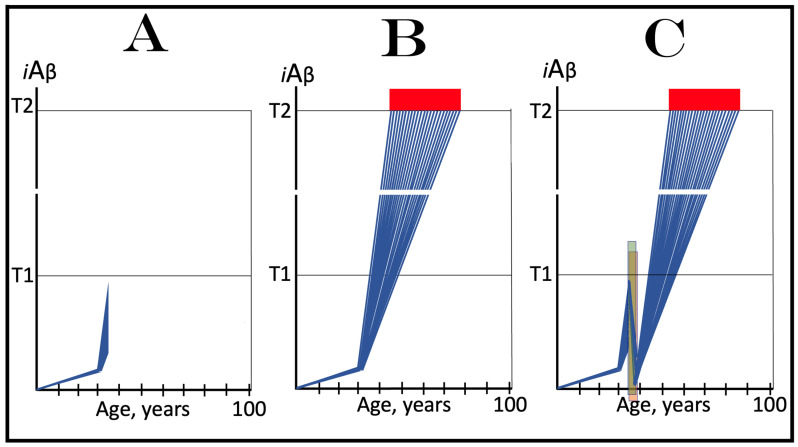
**Composite therapeutic strategy for the prevention of unconventional AD: concurrent transient administration of ISR inhibitors and BACE1 and/or BACE2 activators**. *i*Aβ: intraneuronal Aβ. *Blue lines*: levels of *i*Aβ in individual neurons. *T*1 *threshold*: cellular concentration of *i*Aβ that triggers the elicitation of the neuronal integrated stress response. *T*2 *threshold*: levels of *i*Aβ that trigger apoptosis or necroptosis of neuronal cells. *Red box*: apoptotic zone; within this zone, the neurons have either undergone apoptosis or are dead. *Green box*: duration of the administration of ISR inhibitors. *Orange box*: duration of the depletion of AβPP-derived *i*Aβ: (**A**) Initial state of AβPP-derived *i*Aβ levels in individual neurons. The neuronal integrated stress response is unconventionally elicited and, consequently, the AβPP-independent C100/C99 production pathway is activated. Levels of *i*Aβ produced independently of AβPP rapidly increase but have not yet crossed the T1 threshold. (**B**) Evolution of the initial state in the untreated individual. The AβPP-independent C100/C99 generation pathway remains operational, and the accumulation of *i*Aβ produced independently of AβPP continues uninterrupted; more neurons cross the T2 threshold, thus leading to the end stage of the disease. (**C**) Evolution of the initial state in the treated AD patient. The transient ISR inhibition and BACE activation are implemented concurrently. The former enables the production of BACE1 and BACE2 and thus ensures their availability, and disables the AβPP-independent C100/C99 generation pathway, thus abolishing the influx of *i*Aβ produced independently of AβPP and ensuring its efficient depletion by the latter. Following the treatment, the accumulation of *i*Aβ resumes from a low baseline. At this time, however, the neuronal integrated stress response is unconventionally elicited, due to the persistence of unconventional stressors, and the AβPP-independent C100/C99 generation pathway is unconventionally activated. *i*Aβ produced independently of AβPP rapidly accumulates and crosses the T1 threshold. The disease commences and proceeds unimpeded. The treatment delays the occurrence of AD but only transiently.

Figure 22 illustrates the effect of the composite therapeutic strategy in the prevention of unconventional AD. Panel A of Figure 22 shows the initial state of the levels of iAβ in the individual neurons at the time of the administration of the composite therapy. In this person, the neuronal integrated stress response is unconventionally elicited, and consequently, the AβPP-independent C100/C99 production pathway is activated. Levels of *i*Aβ produced independently of AβPP rapidly increase but have not yet crossed the T1 threshold. Panel B of Figure 22 depicts the evolution of the initial state in the untreated individual. Levels of *i*Aβ produced independently of AβPP continue increasing. When they cross the T1 threshold, the AβPP-independent C100/C99 generation pathway is rendered self-sustainable. AD commences and progresses unimpeded until it reaches the end stage. Panel C of Figure 22 presents the evolution of the initial state during and following the concurrent transient administration of both BACE1 and/or BACE2 activators and ISR inhibitors. In this composite treatment, ISR inhibitors fulfill two functions. First, they reverse the ISR state and thus enable the production of BACE enzymes, making them available for activation. Second, they disable the AβPP-independent C100/C99 generation pathway and thus abolish the influx of *i*Aβ produced independently of AβPP. This allows for the effective depletion of *i*Aβ by the activated BACE1 and/or BACE2. When the treatment is concluded, the accumulation of *i*Aβ resumes de novo. Due to the persistent presence of unconventional stressors, following the withdrawal of the drugs, the neuronal ISR is re-elicited, and consequently, the AβPP-independent C100/C99 generation pathway is reactivated. *i*Aβ produced independently of AβPP rapidly accumulates and crosses the T1 threshold. AD commences and progresses uninterrupted. The treatment certainly did not prevent the disease but, nevertheless, delayed its occurrence by a number of years.

## 48. Effect of Composite Therapeutic Strategy in the Treatment of Unconventional AD: Transient Activation of BACE1 and/or BACE2 Would Be Effective Only in the Presence of the ISR Inhibitors 

**Figure 23:**
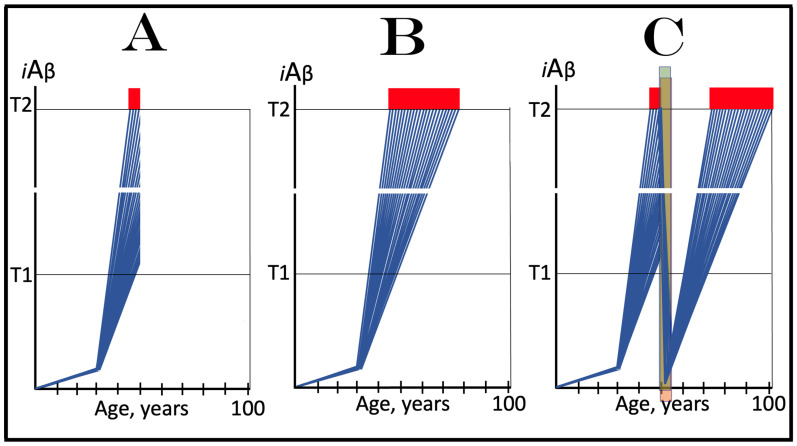
**Composite therapeutic strategy for the treatment of unconventional AD: concurrent transient administration of ISR inhibitors and BACE1 and/or BACE2 activators**. *i*Aβ: intraneuronal Aβ. *Blue lines*: levels of *i*Aβ in individual neurons. *T*1 *threshold*: cellular concentration of *i*Aβ that triggers the elicitation of the neuronal integrated stress response. *T*2 *threshold*: levels of *i*Aβ that trigger apoptosis or necroptosis of neuronal cells. *Red box*: apoptotic zone; within this zone, the neurons have either undergone apoptosis or are dead. *Green box*: duration of the administration of ISR inhibitors. *Orange box*: duration of the depletion of AβPP-derived *i*Aβ: (**A**) Initial state of AβPP-derived *i*Aβ levels in individual neurons. AβPP-derived *i*Aβ has already crossed the T1 threshold in all affected neurons of the AD patient. The neuronal integrated stress response is elicited, the AβPP-independent C100/C99 generation pathway is activated, and *i*Aβ produced independently of AβPP rapidly accumulates. The apoptosis-triggering T2 threshold is crossed in a fraction of the neurons, but the bulk of the affected neurons remain sub-T2. (**B**) Evolution of the initial state in the untreated individual. The AβPP-independent C100/C99 generation pathway remains operational, and the accumulation of *i*Aβ produced independently of AβPP continues uninterrupted; more neurons cross the T2 threshold, thus leading to the end stage of the disease. *i*Aβ produced independently of AβPP accumulates and reaches the T2 threshold, and the disease enters the end stage. (**C**) Evolution of the initial state in the treated AD patient. The transient ISR inhibition and BACE activation are implemented concurrently. The former enables the production of BACE1 and BACE2 and thus ensures their availability, and disables the AβPP-independent C100/C99 generation pathway, thus abolishing the influx of *i*Aβ produced independently of AβPP and ensuring its efficient depletion by the latter. Following the treatment, the progression of AD ceases, and the accumulation of *i*Aβ resumes from a low baseline. At this time, however, the neuronal integrated stress response is unconventionally elicited, due to the persistence of unconventional stressors, and the AβPP-independent C100/C99 generation pathway is unconventionally activated. *i*Aβ produced independently of AβPP rapidly accumulates and crosses the T1 threshold. The disease recurs and proceeds uninterrupted. The treatment provides the temporary reprieve of probably several disease-free years.

Figure 23 illustrates the effects of the composite therapy consisting of the simultaneous administration of ISR inhibitors and BACE activators in the treatment of unconventional AD. Panel A of Figure 22 depicts the initial state of *i*Aβ levels in the individual neurons at the time of the commencement of the composite therapy. In this scenario, unconventional stressors occur, and the neuronal integrated stress response is unconventionally activated when AβPP-derived *i*Aβ has not yet crossed the T1 threshold. Consequently, the AβPP-independent C100/C99 generation pathway is activated, and its *i*Aβ product rapidly accumulates. When it crosses the T1 threshold, the pathway is rendered self-sustainable, and AD commences. *i*Aβ produced independently of AβPP continues its rapid accumulation, crosses the T2 threshold in the fraction of the neurons, and AD symptoms manifest. Panel B of Figure 23 shows the evolution of the initial state in the untreated patient. The accumulation of *i*Aβ produced independently of AβPP in sub-T2 neurons continues unimpeded. When the T2 threshold is reached and crossed in a sufficient fraction of neurons, the disease enters its end stage. Panel C of Figure 23 presents the evolution of the initial state during and following the composite treatment with both ISR inhibitors and BACE1 and/or BACE2 activators. The presence of ISR inhibitors reverses the ISR state and makes BACE enzymes available. It also causes the operation of the AβPP-independent C100/C99 generation pathway to stop and abrogates the influx of *i*Aβ produced independently of AβPP. This enables the activated BACE1 and/or BACE2 to efficiently deplete *i*Aβ to a low baseline and arrest the progression of the disease. When drugs are withdrawn, the neuronal ISR is re-elicited due to the persistent presence of unconventional stressors, and the AβPP-independent C100/C99 generation pathway is unconventionally reactivated. *i*Aβ produced independently of AβPP rapidly accumulates and crosses the T1 threshold. The AβPP-independent C100/C99 generation pathway becomes self-sufficient, AD re-commences and progresses uninterrupted until it reaches the end stage. Thus, the disease eventually recurred but the composite treatment provided several disease-free years prior to its recurrence.

## 49. Effect of Composite Therapeutic Strategy in the Prevention and Treatment of Unconventional AD: Long-Term Activation of BACE1 and/or BACE2 Would Be Efficient Only for the Duration of the ISR Inhibition

As described in the preceding section, the transient composite therapy comprising concurrently administered ISR inhibitors and BACE activators is apparently capable of providing several years of reprieve from the occurrence of AD (when administered preventively) or from the recurrence of the disease (when administered curatively). Can this reprieve period be extended? Above, Section 44 describes and Figure 17 illustrates the implementation of the composite therapy in the treatment of conventional AD. In this therapy, both ISR inhibitors and BACE activators are administered transiently. For ISR inhibitors, this is mandatory in order to avert adverse effects. They should be present only until *i*Aβ, being depleted by activated BACE, reverse-crosses the T1 threshold. Following this reverse crossing, the withdrawal of ISR inhibitors would not trigger the re-elicitation of the ISR because levels of the conventional neuronal ISR-eliciting stressor (i.e., AβPP-derived *i*Aβ) are below the T1 threshold. BACE activators are also administered transiently because they are not needed after a sufficient depletion of *i*Aβ is accomplished, and AβPP-derived *i*Aβ is accumulating at the low, pre-T1 crossing rate, but potentially, their administration could continue for a long duration.

In this context, it could be tempting to extend the reprieve conferred by the composite AD therapy, discussed in the preceding two sections, by administering BACE activators for a long duration in concert with the transient administration of ISR inhibitors in the prevention and treatment of unconventional AD. This, however, is not possible. This is because, in these scenarios (the prevention or treatment of unconventional AD), unconventional stressors capable of eliciting the neuronal integrated stress response are persistently present. Whenever ISR inhibitors are withdrawn, even after the levels of *i*Aβ reverse-cross the T1 threshold, the neuronal ISR is re-elicited, and the AβPP-independent C100/C99 generation pathway is reactivated. More importantly, the production of BACE enzymes is suppressed as a consequence of the ISR state, and the continuous presence of BACE activators becomes irrelevant. Therefore, a long-term administration of BACE activators would be effective only for the duration of the ISR inhibition.

Thus, extending the duration of the administration of BACE activators does not confer additional reprieve in the prevention and treatment of unconventional AD by composite therapy. There is, however, an alternative approach, described in the following section, that can potentially accomplish this.

## 50. Abrogating the Impediment of the ISR-Mediated BACE Suppression in Long-Term Prevention and Treatment of Unconventional AD: Recurrent Transient Simultaneous Administration of BACE Activators and ISR Inhibitors 

As discussed above, the reprieve conferred in both prevention and treatment of unconventional Alzheimer’s disease by the composite therapy comprising concurrently and transiently administered ISR inhibitors and BACE activators is temporary but not insubstantial; it is measured, apparently, in years. A single composite treatment can apparently confer upon its recipient several disease-free years. Second, strategically timed implementation of the composite therapy could double this time period, and the following rounds of therapy could, potentially, triple and quadruple it. If the above-described composite therapy for the prevention and treatment of unconventional AD works in a single application, there are no reasons to anticipate that it would not do so in multiple applications. 

**Figure 24:**
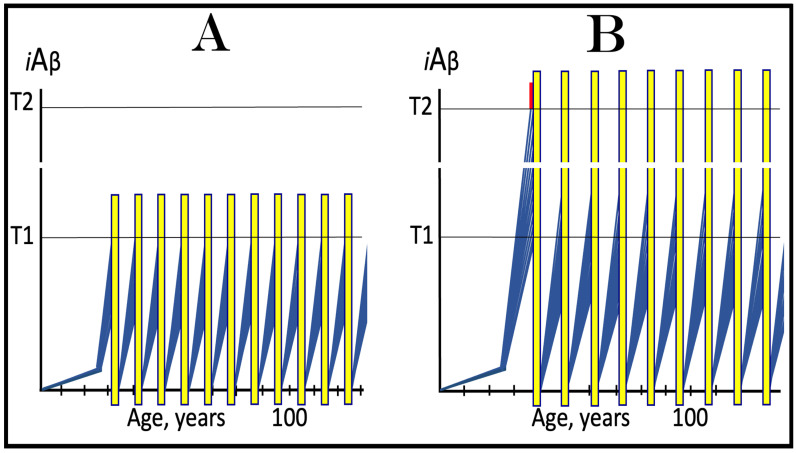
**Overcoming limitations of the composite therapy for the prevention and treatment of unconventional AD: recurrent transient simultaneous administration of BACE activators and ISR inhibitors.** *i*Aβ: intraneuronal Aβ. *Blue lines*: levels of *i*Aβ in individual neurons. *T*1 *threshold*: cellular concentration of *i*Aβ that triggers the elicitation of the neuronal integrated stress response. *T*2 *threshold*: levels of *i*Aβ that trigger apoptosis or necroptosis of neuronal cells. *Red box*: apoptotic zone; within this zone, the neurons have either undergone apoptosis or are dead. *Yellow boxes*: duration of the concurrent administration of ISR inhibitors and BACE activators: (**A**) Recurrent implementation of the composite therapy in the prevention of unconventional AD. At the time of the initial round of the composite therapy, unconventional stressors have already occurred, the neuronal ISR is unconventionally elicited, and the AβPP-independent C100/C99 generation pathway is unconventionally activated. *i*Aβ produced independently of AβPP rapidly accumulates, but its levels are still below the T1 threshold. The transient administration of ISR inhibitors reverses the ISR state, disables the AβPP-independent C100/C99 generation, abolishes the influx of *i*Aβ produced independently of AβPP, restores the production of BACE enzymes, and reinstitutes their availability. The concurrent administration of BACE activators results in the efficient depletion of *i*Aβ. When both ISR inhibitors and BACE activators are withdrawn, the neuronal ISR is re-elicited due to the persistence of unconventional stressors, and the AβPP-independent C100/C99 generation pathway is unconventionally activated. The accumulation of *i*Aβ produced independently from AβPP resumes from a low baseline. Before it reaches the T1 threshold, the following rounds of the composite therapy are implemented repeatedly as needed. The T1 threshold is not crossed, and the disease does not occur as long as the composite therapy continues to be implemented. (**B**) Recurrent implementation of the composite therapy in the treatment of unconventional AD. The initial round of the composite therapy is administered to a symptomatic AD patient. By this time, the neuronal ISR has been unconventionally elicited and the AβPP-independent C100/C99 generation pathway unconventionally activated. *i*Aβ produced independently of AβPP rapidly accumulates and crosses the T1 threshold; the T2 threshold is reached in a fraction of neurons, and AD symptoms manifest. Transiently administered ISR inhibitors reverse the ISR state, restore the availability of BACE enzymes, and abrogate the influx of *i*Aβ produced independently of AβPP. Under these conditions, the concurrently administered BACE1 and/or BACE2 activators efficiently deplete *i*Aβ. Upon the conclusion of the treatment, the neuronal ISR is unconventionally re-elicited, and the AβPP-independent C100/C99 generation pathway is reactivated. The accumulation of *i*Aβ produced independently of AβPP resumes from a low baseline. Before it reaches the AD pathology-causing range, the following rounds of the composite therapy are administered repeatedly as required. The AD pathology-causing range of *i*Aβ concentrations is not reached, and AD symptoms do not recur for the duration of the treatment.

Effects of the recurrent administration of the composite therapy for the prevention and treatment of unconventional AD are illustrated in Figure 24. In panel A of Figure 24, at the time of the initial round of the composite therapy (shown as a single yellow box due to space constraints in the figure), unconventional stressors have already occurred in the affected neurons, the neuronal integrated stress response has been unconventionally elicited and the AβPP-independent C100/C99 generation pathway unconventionally activated. *i*Aβ produced independently of AβPP rapidly accumulates, but its levels are still below the T1 threshold; AD has not yet commenced at this point. The transient administration of ISR inhibitors reverses the ISR state. The production of components essential for the activity of the AβPP-independentC100/C99 generation pathway stops, its operation ceases, and the influx of *i*Aβ produced independently of AβPP is abolished. The reversal of the ISR state also restores the production of BACE enzymes and reinstitutes their availability. The concurrent administration of BACE activators enhances the intra-*i*Aβ-cleaving capabilities of BACE1 and/or BACE2 and results in the efficient depletion of *i*Aβ. When both ISR inhibitors and BACE activators are withdrawn, the neuronal ISR is re-elicited due to the persistent presence of unconventional stressors, and the AβPP-independent C100/C99 generation pathway is unconventionally activated. The accumulation of *i*Aβ produced independently from AβPP resumes from a low baseline and proceeds for a considerable duration. Before it reaches the T1 threshold (which would result in the commencement of AD), the second round of the composite therapy is implemented. The timing of its implementation is determined by the assaying for the appropriate biomarkers. Rounds of the composite therapy are administered repeatedly as needed. The T1 threshold would not be crossed and the disease would not occur as long as the composite therapy continues to be implemented.

In panel B of Figure 24, the initial round of the composite therapy is administered to a symptomatic AD patient. By this time, unconventional stressors appear, the neuronal integrated stress response is unconventionally elicited, and the AβPP-independent C100/C99 generation pathway is unconventionally activated. *i*Aβ produced independently of AβPP rapidly accumulates and crosses the T1 threshold. The AβPP-independent C100/C99 generation pathway is rendered self-sustainable, and AD commences. *i*Aβ produced independently of AβPP accumulates further, reaches the T2 threshold in a fraction of neurons, and AD symptoms manifest. ISR inhibitors administered at this point reverse the ISR state, restore the availability of BACE enzymes, and abrogate the influx of *i*Aβ produced independently of AβPP. Under these conditions, the concurrently administered BACE1 and/or BACE2 activators efficiently deplete *i*Aβ. Upon the conclusion of the treatment and the withdrawal of both BACE activators and ISR inhibitors, the neuronal integrated stress response is unconventionally re-elicited, and the AβPP-independent C100/C99 generation pathway is reactivated. The accumulation of *i*Aβ produced independently of AβPP resumes from a low baseline and proceeds for a considerable duration. Before it reaches the range of concentrations that cause AD pathology, at a stage determined by appropriate biomarkers, the second round of the composite therapy is implemented and the treatment is administered repeatedly, as required. As long as the composite therapy is being readministered as described, the AD pathology-causing range of *i*Aβ concentrations is not reached, and AD symptoms do not recur. 

It should be emphasized that when analyzing the effects of AD therapies in unconventional AD, we considered only the “worst case” scenario, namely that unconventional stressors capable of triggering the elicitation of the neuronal integrated stress response are present continuously from the moment of their occurrence for the remaining lifetime. This may be not the case. If these stressors are no longer present (i.e., either disappear physiologically or are removed therapeutically) by the time of the completion of the transient composite therapy (either a single round or multiple rounds), the effect would be identical to that described for the treatment of conventional AD. Thus, addressing the sources of unconventional stressors could be of significant complementary therapeutic value in the treatment of unconventional AD.

## 51. C99 Generated Independently of AβPP as the Driver of Alzheimer’s Disease: A Valid Option Within the Framework of the ACH2.0

Therapeutic strategies for AD discussed above were based on the presumption that the driver of the disease is *i*Aβ generated in the AβPP-independent pathway. However, as discussed in Section 34 above, there is a distinct possibility that not only BACE enzymes but also the γ-secretase complex is suppressed in AD. The general picture that emerges in this respect is as follows: In conventional AD, the disease is ushered in by AβPP-derived *i*Aβ accumulated over the neuronal ISR-eliciting T1 threshold. In unconventional AD, the disease is initiated by the neuronal integrated stress response elicited by any stressor distinct from AβPP-derived *i*Aβ (i.e., “unconventional stressor”). In both conventional and unconventional forms of AD, the neuronal ISR conditions cause a severe and global reduction in cellular protein synthesis. Components of the AβPP proteolytic pathway are not exempted. The production of AβPP, BACE enzymes, constituents of the γ secretase complex, and consequently, Aβ is severely diminished. At the same time, the neuronal ISR provides components essential for the activity of the powerful AβPP-independent C100/C99 generation pathway and thus enables its operation. The primary product of this pathway is C100 (i.e., N-terminal Met-C99). As discussed above, it is presumably converted into C99 by one of the numerous aminopeptidases with broad specificity; this is the reason for the designation “C100/C99 generation pathway”. The neuronal ISR-associated suppression of the production of BACE enzymes is not relevant to C99 produced independently of AβPP, but that of γ-secretase complex is. Due to the deficiency of γ-secretase, C99 produced independently of AβPP (or at least the bulk of it) is not processed into *i*Aβ but retains its integrity; the designation “C100/C99 generation pathway” emphasizes that C99 is the final product rather than an intermediate. As discussed above (Section 34), this molecule satisfies two principal requirements for a potential driver of AD: it propagates the neuronal ISR state and thus perpetuates its own production, and it is sufficiently toxic to propel AD pathology. We posit, therefore, that C99 generated independently of AβPP is the driver of Alzheimer’s disease in its both conventional and unconventional forms. It is conceivable that the neuronal ISR-mediated suppression of γ-secretase is incomplete and that its degree may vary. In such a case, both C99 and *i*Aβ could contribute to the progression of the disease, and the C99/*i*Aβ ratio could be one of the parameters defining the dynamics of AD.

**Figure 25:**
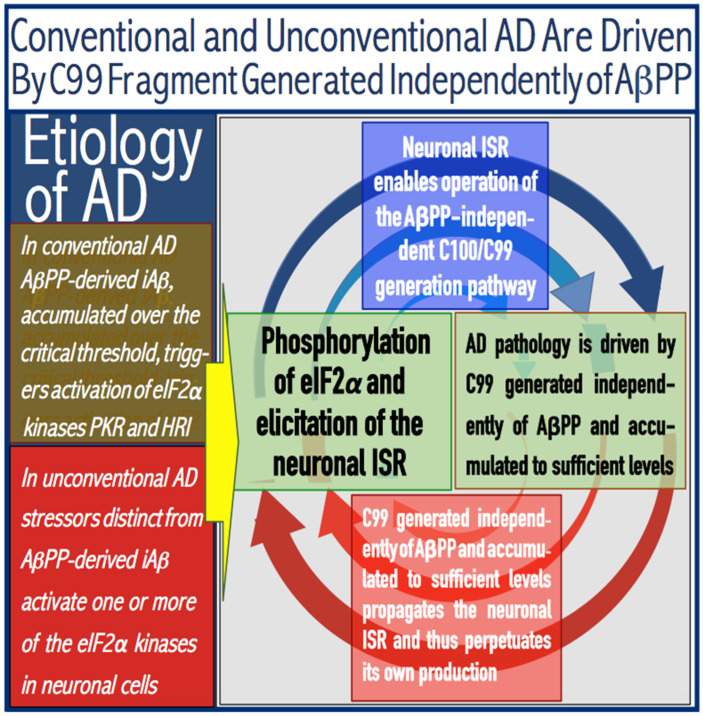
**C99 generated independently of AβPP as the driver of Alzheimer’s disease in its both conventional and unconventional forms. *i*Aβ: intraneuronal Aβ.** *AβPP*: Aβ protein precursor. *C99*: C-terminal fragment of AβPP produced either by the proteolysis of AβPP or independently of AβPP. *eIF2α:* eukaryotic translation initiation factor 2*α*. *PKR and HRI:* kinases capable of phosphorylating eIF2α when activated. *TNFα*: tumor necrosis factor α, potentially capable of activating PKR. *PACT*: PKR activator. *OMA1:* mitochondrial distress-activated mitochondrial protease. *DELE1:* substrate of OMA1; its cleavage leads to the activation of HRI. *ISR:* neuronal integrated stress response; it is elicited by phosphorylation of eIF2α and enables the production of components essential for the operation of the AβPP-independent *i*Aβ generation pathway. In conventional AD, the elicitation of the neuronal ISR is triggered via the activation of PKR and/or HRI kinases and phosphorylation of eIF2α, by AβPP-derived *i*Aβ accumulated over the T1 threshold. In unconventional AD, the elicitation of the neuronal ISR is triggered via the activation of one or more eIF2α kinases, by stressors other than AβPP-derived *i*Aβ. In both forms of AD, the neuronal ISR suppresses, in the context of the inhibition of the global cellular cap-dependent protein synthesis, the production of the components of the AβPP proteolytic pathway in general and of γ-secretase in particular. Simultaneously, it also enables, via the supply of essential components, the operation of the AβPP-independent C100/C99 generation pathway. The resulting C99, produced independently of AβPP, drives AD pathology, propagates the neuronal ISR, and perpetuates its own production.

In its initial formulation, the ACH2.0 postulated that AD is driven by *i*Aβ generated independently from AβPP (i.e., generated by γ-cleavage of C99 produced independently of AβPP). A shift to the determination that AD is driven mainly, if not exclusively, by C99 generated in the AβPP-independent pathway represents the evolution of the ACH2.0. It is fully consistent with the logic of this theory of AD, constitutes its valid option, and is reflected in Figure 25. 

## 52. Effect of the Neuronal ISR-Mediated Suppression of γ-Secretase on the Proposed Therapeutic Strategies for AD

For the reasons given in Section 35 above, the analysis of potential therapeutic strategies was based on the presumption that the C100 fragment generated in that AβPP-independent pathway is processed further first into C99 and then into iAβ. It is important, therefore, to understand the implications of our determination that C99 produced independently of AβPP, at least the bulk of it, is not converted into *i*Aβ but retains its integrity. The present study considers three types of therapeutic strategies for AD. First is the inhibition of the neuronal ISR. The determination that the disease is driven by C99 generated independently of AβPP has no effect whatsoever on ISR inhibitor-based strategy; the production of C99 in the AβPP-independent pathway is affected to precisely the same degree as the AβPP-independent production of *i*Aβ. The second type of therapeutic strategy is the transient activation of BACE1 and/or BACE2 for the prevention of conventional AD and AACD and treatment of AACD. The determination that AD is driven by C99 produced independently of AβPP has no relevance in these cases either. This is because this strategy is implemented only prior to the crossing of the T1 threshold by AβPP-derived *i*Aβ. At this stage, the neuronal ISR is not yet elicited, and the AβPP-independent C100/C99 generation pathway remains inoperative. The third type is the composite ISR inhibition/BACE activation strategy, which is implemented only when the AβPP-independent C100/C99 generation pathway is operational (following the T1 crossing in conventional AD cases and in all cases of unconventional AD). These are the only cases where the determination that C99 rather than *i*Aβ drives the disease could make a difference. However, it apparently does not, and if it does, then very little. Consider the following: Aβ is an integral part of C99. Moreover, the toxicity of C99 apparently emanates from its Aβ portion. When the Aβ is cleaved off C99, the remaining portion (known as AβPP intracellular domain, AICD) has little toxicity [237,238,239,240,241,242,243,244,245,246,247,248,249,250,251,252], and when only sixteen N-terminal amino acid residues (part of its Aβ segment) are cleaved off C99, the toxicity of the remaining C83 substantially diminishes. It could be expected, therefore, that cleavages within the Aβ segment of C99 by intra-*i*Aβ cleaving activities of BACE1 and BACE2 would abrogate the toxicity of the remaining portion of C99. Moreover, during the composite therapy, the administration of ISR inhibitors reverses the ISR state and enables the production of the components of the AβPP proteolytic pathway, including γ-secretase, which would cleave C99 generated independently of AβPP to produce *i*Aβ, which would, in turn, be cleaved by the activated BACE1 and/or BACE2.

## 53. Conclusions: Evolution of the Theory

The ACH2.0 initially defined AD as a disease triggered by AβPP-derived *i*Aβ accumulated over the critical threshold and driven by *i*Aβ generated in the AβPP-independent pathway. The ACH2.0 described in the present Perspective retains its general structure and, more importantly, its logic. However, it evolved considerably. First came the realization that Alzheimer’s disease is not the disorder of *i*Aβ, certainly not of *i*Aβ derived in the AβPP proteolytic pathway. AD is rather the disease of the neuronal integrated stress response. The neuronal ISR, sustainably elicited in ANY way, is the actual trigger of AD. In this perspective, *i*Aβ accumulated to sufficient levels is only one of the numerous stressors capable of eliciting the neuronal integrated stress response and thus triggering the disease. This realization necessitated the separation of the AD cases into two categories: conventional and unconventional. In the former, the neuronal ISR is elicited, and the disease is initiated by AβPP-derived *i*Aβ accumulated to sufficient levels, whereas in the latter category, numerous stressors distinct from AβPP-derived *i*Aβ elicit the neuronal integrated stress response. This separation appears asymmetrical, and the two categories seem grossly uneven: only one causative stressor (AβPP-derived *i*Aβ) in one category and a multitude of potential stressors in another. The reason for such classification is conceptual and qualitative rather than quantitative or arbitrary: only in the conventional AD category is the AβPP-independent C100/C99 generation pathway (presumably yielding *i*Aβ) self-sustainable from the instance of its activation, whereas in the unconventional AD category, this pathway may be activated transiently many times over the lifetime of an individual with no immediate effect if the T1 threshold is not crossed; in such a case, its operation would cease with the withdrawal of the stressor.

The present study takes the evolution of the ACH2.0 even further. It concludes that in AD-affected neurons, the production of Aβ in the AβPP proteolytic pathway is suppressed. This conclusion appears heretical and even moronic in terms of the ACH but is perfectly trivial in the ACH2.0 perspective. This is because AβPP-derived Aβ is the driving force of the disease in the former but redundant, after it triggers the elicitation of the neuronal ISR, in the latter. In fact, it is the neuronal integrated stress response, which suppresses the production of its own activator in this case (after being sustainably elicited). The suppression of the production of AβPP-derived Aβ is, apparently, a proverbial “tip of the iceberg” because other components of the AβPP proteolytic pathway, including, importantly, BACE enzymes and constituents of the γ-secretase complex, are not exempted from the global suppression of cellular protein synthesis under the neuronal ISR conditions in both conventional and unconventional AD. The neuronal ISR-mediated suppression of BACE enzymes is of little consequence in the principal terms of the ACH2.0. In the disease, C99 is produced independently of AβPP, and the β-site cleavage is not required for its generation. This suppression, however, is of great importance in terms of the potential therapeutic strategies for AD. In terms of the ACH2.0, the most effective therapeutic strategy for AD, in its either conventional or unconventional forms and both preventive and curative, is the depletion of *i*Aβ. And the best way, in fact, the only way available to us currently, is the activation of BACE1 and/or BACE2. Under the neuronal ISR conditions, this is not feasible. Therefore, the implementation of this strategy requires that in cases when the neuronal ISR has been elicited, BACE activators be administered concurrently with ISR inhibitors. In such a composite therapy, the ISR inhibition accomplishes two purposes: it enables the production of BACE enzymes and thus restores their availability and simultaneously disables the AβPP-independent production of *i*Aβ (or only C99) and thus abolishes its influx, allowing for the efficient depletion of *i*Aβ and/or the *i*Aβ portion of C99. It should be emphasized that, in this paradigm, the neuronal ISR-mediated suppression of conventional cellular translation, including that of AβPP, would result in AβPP dyshomeostasis, which has emerged recently as a potentially significant contributor to AD pathology [253]. Importantly, the therapeutic strategies proposed above and elsewhere [9] would not only abrogate the AβPP-independent production of C99 but also reverse the neuronal ISR and restore the AβPP homeostasis.

In contrast (in fact, in reverse) to consequences of the neuronal ISR-mediated suppression of BACE enzymes, the suppression of γ-secretase in AD-affected neurons is of considerable consequence in the principal terms of the ACH2.0 but of little importance in terms of its effects on the proposed therapeutic strategies for AD. In the principal terms of the theory, it replaces one of its major attributes, namely the nature and identity of the driver of the disease. It is apparently not *i*Aβ produced independently of AβPP but rather C99 generated in the AβPP-independent pathway that drives AD pathology. Although this is a significant change, it is consistent with the logic of the theory and fits organically into its framework. On the other hand, in terms of its impact on the proposed therapeutic strategies for AD, it is apparently insignificant. This is because the toxicity of C99 is conferred on it mainly by its N-terminal Aβ segment and is apparently abrogated by BACE1 and/or BACE2 cleavages.

It is important to emphasize that the analysis presented in the present Perspective, as well as in preceding studies [1,2,3,4,5,6,7,8,9], applies to the selected sub-populations of neurons referred to as “affected” neurons. This is because not all neurons of a single brain are affected simultaneously in AD [4,254,255,256] but commit to the disease rather sequentially. What is the basis for such a sequential order? In Section 14 above, we concluded that sub-populations of individuals susceptible to AD are not random but defined by lower than average extent of the T1 threshold and higher than average rate of accumulation of AβPP-derived *i*Aβ. The same logic may apply to individual neurons in a single brain, which could be affected by conventional AD in the order of the crossing of their T1 thresholds by AβPP-derived *i*Aβ. It appears, therefore, that the order of the entry of individual neurons into Alzheimer’s disease could be decided by the cumulative effect of the extent of their T1 thresholds and the rates of accumulation of AβPP-derived *i*Aβ, the principal factors determining the timing of the elicitation of the neuronal ISR and, consequently, of the activation of the AβPP-independent C100/C99 generation pathway. In an alternative interpretation [4], all neurons cross the T1 threshold within the narrow time window but have different efficiencies of the AβPP-independent C100/C99 generation pathway (due, possibly, to their differential utilization of TSSs of the AβPP gene; see Section 19), or the extents of their T2 thresholds are different in different neuronal sub-populations [4]. Importantly, whatever interpretation is correct, the therapeutic strategies described above would be equally effective.

In conclusion, the ACH2.0 theory of AD has evolved considerably since its inception. It has been highly productive in yielding potentially effective therapeutic strategies for AD. Its evolution will, undoubtedly, continue; this is the way of science.

What next?
